# Agreement Between Self‐Reported Antirheumatic Medications and Pharmaceutical Claims in an Australian Inflammatory Arthritis Cohort

**DOI:** 10.1002/acr2.70105

**Published:** 2025-10-17

**Authors:** Tom Lynch, Claire Barrett, Rachel Black, Rachelle Buchbinder, Graeme Carroll, Vibhasha Chand, Catherine L. Hill, Marissa Lassere, Sue Lester, Oscar Russell, Premarani Sinnathurai, Lyn March

**Affiliations:** ^1^ Sydney Musculoskeletal Health, Kolling Institute, Faculty of Medicine and Health, The University of Sydney and the Northern Sydney Local Health District and Florance and Cope Professorial Department of Rheumatology, Royal North Shore Hospital Sydney New South Wales Australia; ^2^ Department of Rheumatology, Redcliffe Hospital, Redcliffe, Queensland, Australia, and Department of Medicine, Faculty of Medicine The University of Queensland Brisbane Queensland Australia; ^3^ Rheumatology Unit, The Queen Elizabeth Hospital and Discipline of Medicine, Faculty of Health and Medical Sciences, The University of Adelaide, Adelaide, South Australia, Australia, and Rheumatology Research Group, Basil Hetzel Institute for Translational Health Research The Queen Elizabeth Hospital Woodville South South Australia Australia; ^4^ Musculoskeletal Health and Wiser Health Care Units, School of Public Health and Preventive Medicine Monash University Melbourne Victoria Australia; ^5^ ArthroCare Perth Western Australia Australia; ^6^ Department of Rheumatology, St George Hospital and Department of Medicine, University of New South Wales School of Population Health Sydney New South Wales Australia

## Abstract

**Objective:**

To assess agreement between prescription claims data and self‐reported medication use via longitudinal questionnaires in the Australian Rheumatology Association Database inflammatory arthritis cohort and to identify predictors of discordant self‐reports.

**Methods:**

Agreement was determined between longitudinal questionnaire self‐reports (2012–2023) of disease‐modifying antirheumatic drug (DMARD), glucocorticoid, anti‐inflammatory, and analgesic use and Australian reference standard prescription medication dispensing data (Pharmaceutical Benefits Scheme) using Cohen's kappa, sensitivity, positive predictive value (PPV), and negative predictive value (NPV). Analyses were repeated using four look‐back windows of dispensing data (1, 3, 6, and 12 months) before each questionnaire to characterize variations in agreement metrics at the individual medication level. Predictors of discordant self‐reports were explored using multivariable logistic regression.

**Results:**

In our study population (N = 3,407, 67% female, 93.7% White, 64.4% with rheumatoid arthritis, 18.5% with psoriatic arthritis, 14.6% with ankylosing spondylitis, 2.6% with juvenile idiopathic arthritis), agreement with prescription claims data was substantial to high for DMARDs (κ 0.67–0.95, sensitivity 0.69–0.96, PPV 0.64–0.96, NPV 0.9–1), substantial for prescription‐only nonopioid analgesics and oral prednisolone/prednisone (κ 0.66–0.80, sensitivity 0.65–0.88, PPV 0.68–0.77, NPV 0.93–1), and moderate to substantial for prescription‐only opioid analgesics (κ 0.48–0.7, sensitivity 0.57–0.74, PPV 0.36–0.69, NPV 0.94–1). A 3‐month look‐back window optimized agreement for most medications, whereas 6‐ and 12‐month windows further improved agreement for specific drugs. No consistent predictors of discordant self‐reports were identified, though greater self‐rated disability severity and poorer overall health showed the most consistent associations with discordance.

**Conclusion:**

Agreement between self‐reported and pharmaceutical claims data was moderate to high. Poorer overall health and disability may impact accuracy of medication self‐report.

## INTRODUCTION

Collection of self‐reported medicines use is important in several research domains, from pharmacoepidemiology to health economics and precision medicine; however, verifying use can be challenging due to limitations of source data.^1^, ^2^, ^3^ In the field of rheumatology, there is great interest in artificial intelligence–powered big data analytics heralding a new era of predictive decision support tools for optimizing therapeutic management.^4^ These advances may leverage self‐reported data from large well‐characterized patient registries and biobank cohorts. Understanding the validity and accuracy of such data is crucial for associated analytics, predictive modeling, and the potential for clinical translation.^5^

Self‐reports, collected via questionnaire or interview, are generally assumed to reflect actual intake of medication. However, they depend on individuals’ abilities to volunteer such information and can be prone to underreporting^6^ and human error related to recall bias,^7^ poor health status,^8^ and polypharmacy.^9^, ^10^ Accuracy can be improved using objective biochemical measurements or by counting unused medication (“brown bag” method),^8^, ^11^ but limited study resources and logistical challenges can hinder use of these labor‐intensive methods.^12^

A 2021 systematic review identified wide variation in methodologic approaches to validating self‐reports and a paucity of evidence regarding patient characteristics predictive of valid self‐report.^13^ Most studies have focused on self‐reports of current use; few have attempted to verify past or ever use, duration of use, or dosages. Medication lists from medical or pharmacy records can correlate strongly with use^14^ but may be incomplete, out of date, and limited to single providers and may not reflect actual patient adherence and persistence.^3^ Prescription claims databases are the most often used reference standard for medicines dispensed but do not capture information on actual use of medicines by patients.^13^ In Australia, the federal government administers the Pharmaceutical Benefits Scheme (PBS), a national program providing residents with access to subsidized prescription medicines.^15^ Like other prescription claims databases, PBS‐subsidized prescription data have inherent limitations as a reference standard; although the data capture PBS‐subsidized prescriptions when they are filled, they don't include private and off‐label prescriptions, over‐the‐counter (OTC) medicines, complementary medicines, illicit drugs, and medications provided to most public hospital in‐patients, with some exceptions.^16^, ^17^, ^18^ Given these limitations, neither self‐report nor prescription claims data can be considered a true gold standard for assessing medicine use. In this study, PBS claims data are used as a de facto reference to enable comparison with self‐report using standard methods.

People with cancer appear to have been the most frequently examined disease group for agreement of self‐reported medication intake with other data sources.^13^ Only a small number of studies have specifically examined medications related to rheumatic diseases.^16^, ^19^ Only one study focused on validating antirheumatic medication self‐report from people with inflammatory arthritis^20^; limitations included use of a single academic center, small sample size, and lack of comparative pharmaceutical claims data.

In this study, we sought to (1) investigate convergent validity of current medication self‐report by participants in an Australian inflammatory arthritis registry using pharmaceutical claims (dispensing) data as the reference standard and (2) explore sociodemographic and health‐related predictors of discordant self‐reporting in this cohort.

## PATIENTS AND METHODS

### Study population

We used longitudinal data from the Australian Rheumatology Association Database (ARAD), a national registry of 6,567 patients with rheumatoid arthritis (RA), psoriatic arthritis (PsA), ankylosing spondylitis (AS; radiographic axial spondyloarthritis), and juvenile idiopathic arthritis (JIA).^21^ Patients were enrolled by self‐referral or by their rheumatologists (n = 308) across multiple public and private rheumatology clinic sites nationwide. Ethics approval for ARAD has been obtained from the Cabrini Human Research Ethics Committee (initial: 12–23–04–01), the Northern Sydney Local Health District Human Research Ethics Committee (ongoing: 2019/ETH10386), and several other committees and organizations across Australia. All participants provided written informed consent. This study complies with the Declaration of Helsinki and was approved by the University of Sydney Human Research Ethics Committee, Sydney, Australia (2021/135). Participants completed self‐administered online or paper questionnaires every six months for the first two years of their participation. Then to minimize longitudinal burden, survey frequency was reduced to annually, continuing for as long as participants remained enrolled. Questionnaires captured detailed information on demographics, medication use, quality of life, and health status. Sixty‐eight percent of participants consented to linkage with administrative health data, including PBS claims. The data and analysis scripts that support the findings of this study are available from the ARAD, but restrictions apply to the availability of these data, which were used under ethical approval for the present study and so are not publicly available. Data are, however, available from the authors upon reasonable request and with permission from the ARAD Access Committee.

### Inclusion and exclusion criteria for agreement analysis

**Figure 1:**
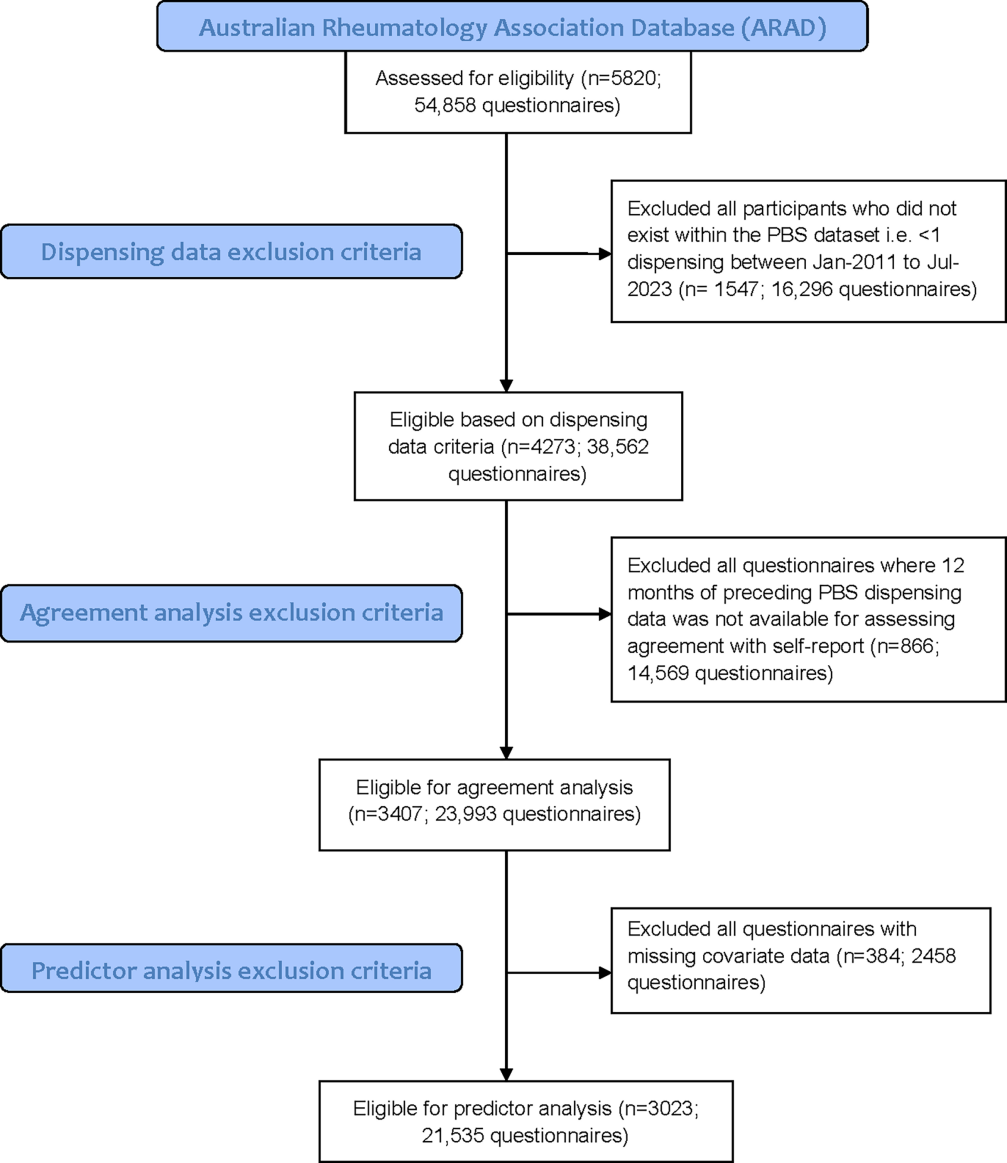
Flow diagram of inclusion and exclusion criteria used. PBS, Pharmaceutical Benefits Scheme.

Although ARAD has enrolled participants^22^ since 2001, linked prescription claims dispensing data were available only from January 2011 to June 2023 in the subset of participants who provided data linkage consent. To assess agreement with a “current” self‐report status, a maximum look‐back window of 12 months of preceding claims data was selected (see Defining medication exposure and validity); we therefore included only the 3,407 participants who completed any questionnaires between January 2012 and June 2023, with PBS dispensing data available for comparison (Figure 1).

### Self‐reported medication data format

Over the 11‐year study period, eligible participants provided self‐reported medicines information via 23,993 longitudinal online (69%) and paper (31%) self‐administered questionnaires for current use of five groups of medications: (1) biologic and targeted synthetic disease‐modifying antirheumatic drugs (b/tsDMARDs), (2) conventional synthetic DMARDs (csDMARDs), (3) glucocorticoids, (4) nonopioid analgesics (including nonsteroidal anti‐inflammatory drugs and paracetamol), and (5) opioid analgesics (Supplementary Table S1). For DMARDs and glucocorticoids, medication status was self‐reported with four options: “never taken,” “currently taking,” “stopped taking,” or “don't know” (Supplementary Figure S1a and b). For other anti‐inflammatories and analgesics, current use was indicated with a simple checkbox response (Supplementary Figure S1c). In the 6‐ and 12‐month follow‐up questionnaires, previous responses for DMARDs and glucocorticoids were prepopulated to facilitate longitudinal status updates (paper forms used TeleForm data merging).

To account for possible misinterpretations, five “other medication” free‐text fields (Supplementary Figure S1c) were also analyzed and manually recoded where mismatches with medication status fields were apparent (Supplementary Table S2). A total of 858 recodes across 29 medicines were performed, representing 0.1% of overall responses analyzed; the majority were for paracetamol (n = 365, 1.5% of responses), prednisolone/prednisone (n = 52, 0.21%), etanercept (n = 50, 0.21%), ibuprofen (n = 48, 0.20%), and morphine (n = 45, 0.19%).

### Prescription medication claims data

Deterministically matched PBS prescription medication claims data were sourced from Services Australia following approval of the ethics‐approved consent materials.^23^ Dispensations included date of supply, PBS item code, and Anatomical Therapeutic Chemical (ATC) code.^24^ ATC codes are more stable than PBS item codes, which vary by listing status and strength variations.^25^, ^26^ Although PBS data are used in this study as the de facto reference for evaluating self‐report, it is acknowledged that PBS claims reflect medication dispensing rather than actual consumption and do not capture all sources of medication use, including private prescriptions and OTC medications. Of note is that high‐cost prescription medications, such as the b/tsDMARDs commonly used to treat inflammatory arthritis, are rarely dispensed privately due to their expense.

### Mapping self‐reported medications to PBS pharmaceutical claims

Thirty‐nine medications in the ARAD questionnaire were mapped to historical prescription items in the PBS Item Code to Drug Mapping File to gather relevant ATC and PBS item codes for validation (Supplementary Table S1).^27^ Listing dates were extracted from PBS schedule records in the Publications Archive (available from 2003) and cross referenced with the *Australian Prescriber* journal (New Drug listings).^28^ Three medicines (anakinra, apremilast, and dextropropoxyphene) were excluded from this analysis because they were not PBS listed over most of the study date range (Supplementary Table S1).

### Defining medication exposure and validity

Convergent validity was examined using seven‐digit ATC or six‐digit PBS item code matching by defining a self‐report as a true positive when a “current” status was entered in a questionnaire (Supplementary Figure S1) and at least one corresponding medication dispensation was found in the PBS dataset within a defined look‐back window before the questionnaire submission date. Seven‐digit ATC codes were used where questionnaire items were completely covered by their ATC grouping, for example, all etanercept use is captured using ATC code L04AB01. PBS item code matching was used when additional differentiation beyond seven‐digit ATC codes was required due to drug form specifications in the question (eg, oral methotrexate vs injectable methotrexate).

A true negative current self‐report was defined when “never taken” or “stopped taking'” was selected in a nonbinary question (four‐item radio button or dropdown) or when a checkbox was left unchecked in a binary question, with no corresponding dispensing in the look‐back window. An additional assumption was made for “stopped taking” responses where a related dispensing occurred within the look‐back window: if a corresponding “date stopped” was self‐reported, these were classified as a true negative when the pharmacy supply date occurred before the stop date but as a false negative when the supply date occurred after the stop date. Responses of “don't know” in nonbinary questions were excluded from agreement analysis. We did not attempt to validate a status of “never taken” given the lack of sufficient retrospective claims data to accurately confirm a medication had never been dispensed.

Given varied prescribing, dosing, and dispensing patterns of included medications, the analysis was repeated using four look‐back windows of claims data preceding the questionnaire completion date (1 month [30 days], 3 months [90 days], 6 months [182 days], and 12 months [365 days]) to ascertain the optimal look‐back window per medication. The optimal look‐back window was defined as the window with the highest agreement (kappa) with self‐report, which varied by medication. Where two windows had the same kappa, the window with the highest sensitivity was selected as the optimal window.

### Missing data

Quality control for ARAD electronic questionnaires included use of mandatory field settings to limit missing self‐report data. Postal paper questionnaires were received and reviewed by the data manager before data entry; some missing self‐report data were possible where medication status questions were left blank by the participant. For simple checkbox medication status questions, our analysis assumes an unchecked box implied the participant was not currently taking the medication, acknowledging this interpretation may overestimate negative self‐reports for participants who did not know.

### Statistical analysis

Data manipulation and statistical analysis were performed in RStudio^29^ (R v4.4.2),^30^ primarily using the tidyverse^31^ (v2.0.0), epiR^32^ (v2.0.80), and base stats packages. Lookup tables were created by merging the linked ARAD and PBS datasets. Each medication self‐report was classified with a validity score to generate 2 × 2 contingency tables per look‐back window (self‐report [yes or no], PBS claim [yes or no]). We reported level of agreement using four metrics, (1) unadjusted Cohen's kappa method of rating interrater reliability (primary agreement measure),^33^, ^34^, ^35^ (2) sensitivity (proportion of PBS medication use identified correctly by self‐reporting), (3) positive predictive value (PPV; proportion of positive self‐reports confirmed by claims data), and (4) negative predictive value (NPV; proportion of negative self‐reports confirmed by absent claims data). κ scores of ≤0.20 were considered low/poor agreement; 0.21–0.40, fair; 0.41–0.60, moderate; 0.61–0.80, substantial; and >0.80, high agreement.^36^ Ninety‐five percent confidence intervals (CIs) were calculated.

### Predictors of discordant self‐report

A discordant self‐report was defined as either a false positive (self‐report [yes], PBS claim [no]) or false negative (self‐report [no], PBS claim [yes]) classification using the optimal look‐back window identified for each medication. To explore predictors of discordant self‐report using the questionnaire as our unit of analysis, we first conducted univariate logistic regressions for prescription‐only medications using the following categories as independent variables: age (years; continuous), sex (female, male), education status (tertiary qualified, not tertiary qualified), marital status (married, not married), smoking status (current smoker, non–current smoker), disease duration (years; continuous), socioeconomic status (SES; Index of Relative Socio‐economic Advantage and Disadvantage [IRSAD] Socio‐Economic Indexes for Areas (SEIFA) by Statistical Area Level 1 percentile; continuous), mental health status (EuroQol 5‐dimension 3‐level [EQ‐5D‐3L] moderate‐extreme anxiety or depression, none), pain (EQ‐5D‐3L moderate‐extreme pain or discomfort, none), self‐rated health state (EQ‐5D visual analog scale [VAS]; continuous), functional status (Health Assessment Questionnaire disability index [HAQ‐DI] score; continuous), and questionnaire modality (online, paper). SES was determined by the IRSAD (SEIFA 2011), a score reflecting economic and social conditions of people and households within an Australian area.^37^

Due to some missing covariate data, the sample size was reduced from 23,993 questionnaires to 21,535 for complete case analysis (Figure 1). Medications were excluded from the predictor analysis where a minimum of 10 cases with the least frequent outcome for each dichotomous independent variable was not available (Supplementary Table S3). Subsequently, multivariable regression was performed using the significant variables from the univariate analysis, adjusted for multiple comparisons using the Benjamini‐Hochberg method to control the false discovery rate (α = 0.05).^38^ Odds ratios (ORs) with 95% CIs were reported. OR effect sizes (ES) were estimated for dichotomous categories using the rules of thumb by Chen et al.^39^ ORs of <1.68 were considered very small; 1.68 to <3.47, small; 3.47 to <6.71, medium; and ≥6.71, large.

## RESULTS

A total of 3,407 ARAD participants met eligibility criteria for the validation analysis (Table 1). The mean age at the first questionnaire within the study period was 55.1 (SD 14.4) years. The median number of questionnaires completed per participant during the study period was 7 (range 1–11; interquartile range 6). Participants were mostly female (67.1%), White (93.7%), Australia born (75.8%), and English speaking (97.5%). Half (50%) were tertiary educated. Primary diagnoses were RA (64.4%), PsA (18.5%), AS (14.6%), and JIA (2.6%). The mean disease duration was 15.9 (SD 11.2) years.

**Table 1:** Characteristics of study participants*

Characteristic and subgroup	Study population (N = 3,407)
Age, mean (SD), y^a^	55.1 (14.4)
Female, % (n)	67.1 (2,286)
Diagnosis, % (n)	
Rheumatoid arthritis	64.4 (2,193)
Psoriatic arthritis	18.5 (629)
Ankylosing spondylitis	14.6 (498)
Juvenile idiopathic arthritis	2.6 (87)
Disease duration, mean (SD), y	15.9 (11.2)
Ethnicity/ancestry, % (n)	
White	93.7 (3,193)
Asian	2.1 (72)
Aboriginal or Torres Strait Islander	0.6 (22)
Other	3.5 (120)
Australia born, % (n)	75.8 (2,583)
Main language spoken at home English, % (n)	97.5 (3,323)
Highest educational qualification, % (n)^b^	
Never attended school	0.1 (4)
Primary school	2.8 (95)
Some high school	22 (750)
Completed high school	24.6 (838)
University, TAFE, CAE, or other tertiary institution	50 (1702)
Unknown	0.5 (18)
Ever a regular smoker, % (n)	48.8 (1,662)
Self‐rated disability severity, % (n)^c^	
Mild to moderate	63.1 (2,150)
Moderate to severe	29.6 (1,009)
Severe to very severe	5.5 (187)
Unknown	1.8 (61)
Questionnaire mode, % (n)	
Online	69 (16,547)
Paper	31 (7,446)

* CAE, Centre for Adult Education (Victoria, Australia); HAQ, Health Assessment Questionnaire; TAFE, Technical and Further Education (Australia).

^a^ At study baseline.

^b^ During study period.

^c^ Mean HAQ score for study period.

### Agreement and convergent validity

**Figure 2:**
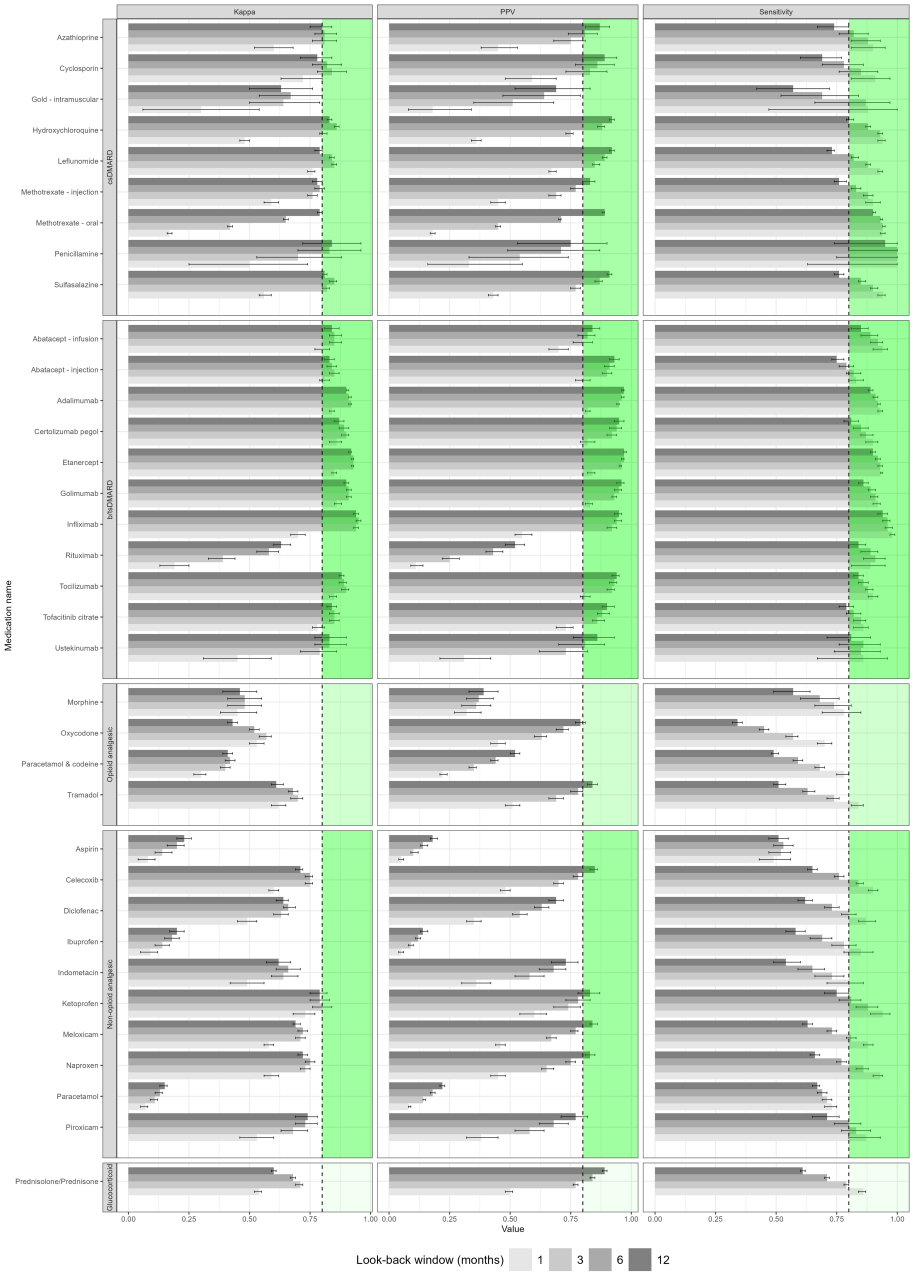
Faceted grouped bar chart showing comparative agreement (kappa), PPV, and sensitivity of medication self‐report, compared with PBS dispensing data as the reference standard, across 1‐, 3‐, 6‐, and 12‐month look‐back windows, with 95% confidence intervals. Vertical shaded area represents values >0.8. b/tsDMARD, biologic and targeted synthetic disease‐modifying antirheumatic drug; csDMARD, conventional synthetic disease‐modifying antirheumatic drug; PBS, Pharmaceutical Benefits Scheme; PPV, positive predictive value.

Table 2 shows the frequency, agreement (unadjusted kappa), sensitivity, PPV, and NPV for self‐reports compared with reference standard PBS prescription pharmaceutical claims data for the optimal look‐back window before the questionnaire submission date. Supplementary Tables S4 to S7 provide the underlying data tables demonstrating the comparative agreement metrics across 1‐, 3‐, 6‐, and 12‐month look‐back windows. The look‐back period of claims data that showed the highest agreement varied across medications: a 3‐month period showed the highest agreement for 17 medications, a 6‐month period showed the highest agreement for 11 medications, and a 12‐month period showed the highest agreement for 7 medications, whereas a 1‐month period did not yield the highest agreement for any medications. Figure 2 demonstrates the comparative agreement, PPV, and sensitivity across 1‐, 3‐, 6‐, and 12‐month look‐back windows.

**Table 2:** Frequency, agreement, sensitivity, PPV, and NPV of rheumatology‐related medication self‐reports compared with PBS prescription pharmaceutical claims data (Australian reference standard) for the optimal exposure window before the questionnaire submission date for the Australian Rheumatology Association Database cohort (questionnaire period: 2012–2023; PBS supply period: 2011–2023)*

Medication	Prescription only	TN	FN	FP	TP	N/A	κ (95% CI)	Sensitivity (95% CI)	PPV (95% CI)	NPV (95% CI)	Optimal window, mo^a^
Class: csDMARD											
Azathioprine	Yes	23,560	18	43	129	243	0.81 (0.76–0.86)	0.88 (0.81–0.93)	0.75 (0.68–0.81)	1 (1–1)	3
Cyclosporin	Yes	23,651	13	15	73	241	0.84 (0.78–0.9)	0.85 (0.76–0.92)	0.83 (0.73–0.9)	1 (1–1)	3
Gold (intramuscular)	Yes	23,802	11	14	25	141	0.67 (0.54–0.8)	0.69 (0.52–0.84)	0.64 (0.47–0.79)	1 (1–1)	6
Hydroxychloroquine	Yes	20,097	385	412	2,888	211	0.86 (0.85–0.87)	0.88 (0.87–0.89)	0.88 (0.86–0.89)	0.98 (0.98–0.98)	6
Leflunomide	Yes	20,734	311	402	2,330	216	0.85 (0.84–0.86)	0.88 (0.87–0.89)	0.85 (0.84–0.87)	0.99 (0.98–0.99)	3
Methotrexate (injection)	Yes	22,600	166	241	815	171	0.79 (0.77–0.81)	0.83 (0.81–0.85)	0.77 (0.75–0.8)	0.99 (0.99–0.99)	6
Methotrexate (oral)	Yes	10,615	1,174	1,343	10,759	102	0.79 (0.78–0.8)	0.9 (0.9–0.91)	0.89 (0.88–0.89)	0.9 (0.89–0.91)	12
Penicillamine	Yes	23,680	1	6	18	288	0.84 (0.72–0.96)	0.95 (0.74–1)	0.75 (0.53–0.9)	1 (1–1)	12
Sulfasalazine	Yes	21,233	328	292	1,906	234	0.85 (0.83–0.86)	0.85 (0.84–0.87)	0.87 (0.85–0.88)	0.98 (0.98–0.99)	6
Class: b/tsDMARD											
Abatacept (infusion)	Yes	23,472	33	91	367	30	0.85 (0.83–0.88)	0.92 (0.89–0.94)	0.8 (0.76–0.84)	1 (1–1)	3
Abatacept (injection)	Yes	23,052	154	79	706	2	0.85 (0.83–0.87)	0.82 (0.79–0.85)	0.9 (0.88–0.92)	0.99 (0.99–0.99)	3
Adalimumab	Yes	18,100	423	277	5,119	74	0.92 (0.91–0.92)	0.92 (0.92–0.93)	0.95 (0.94–0.95)	0.98 (0.97–0.98)	3
Certolizumab pegol	Yes	23,322	78	45	541	7	0.9 (0.88–0.91)	0.87 (0.85–0.9)	0.92 (0.9–0.94)	1 (1–1)	3
Etanercept	Yes	18,482	365	221	4,890	35	0.93 (0.92–0.93)	0.93 (0.92–0.94)	0.96 (0.95–0.96)	0.98 (0.98–0.98)	3
Golimumab	Yes	22,342	145	105	1,397	4	0.91 (0.9–0.92)	0.91 (0.89–0.92)	0.93 (0.92–0.94)	0.99 (0.99–0.99)	3
Infliximab	Yes	23,112	31	41	700	109	0.95 (0.94–0.96)	0.96 (0.94–0.97)	0.94 (0.93–0.96)	1 (1–1)	6
Rituximab	Yes	23,172	68	327	354	72	0.63 (0.6–0.67)	0.84 (0.8–0.87)	0.52 (0.48–0.56)	1 (1–1)	12
Tocilizumab	Yes	22,544	153	106	1,175	15	0.9 (0.88–0.91)	0.88 (0.87–0.9)	0.92 (0.9–0.93)	0.99 (0.99–0.99)	3
Tofacitinib citrate	Yes	23,232	103	88	562	8	0.85 (0.83–0.87)	0.85 (0.82–0.87)	0.86 (0.84–0.89)	1 (0.99–1)	3
Ustekinumab	Yes	23,900	10	15	63	5	0.83 (0.77–0.9)	0.86 (0.76–0.93)	0.81 (0.7–0.89)	1 (1–1)	6
Class: opioid analgesic											
Morphine	Yes	23,684	34	177	98	0	0.48 (0.41–0.55)	0.74 (0.66–0.81)	0.36 (0.3–0.42)	1 (1–1)	3
Oxycodone	Yes	21,987	650	507	849	0	0.57 (0.54–0.59)	0.57 (0.54–0.59)	0.63 (0.6–0.65)	0.97 (0.97–0.97)	3
Paracetamol and codeine	No	19,049	1,159	2,134	1,651	0	0.42 (0.4–0.44)	0.59 (0.57–0.61)	0.44 (0.42–0.45)	0.94 (0.94–0.95)	6
Tramadol	Yes	22,517	293	368	815	0	0.7 (0.67–0.72)	0.74 (0.71–0.76)	0.69 (0.66–0.72)	0.99 (0.99–0.99)	3
Class: nonopioid analgesic											
Aspirin	No	21,350	392	1,843	408	0	0.23 (0.2–0.26)	0.51 (0.47–0.55)	0.18 (0.17–0.2)	0.98 (0.98–0.98)	12
Celecoxib	Yes	21,520	285	647	1,541	0	0.75 (0.73–0.76)	0.84 (0.83–0.86)	0.7 (0.68–0.72)	0.99 (0.99–0.99)	3
Diclofenac	No	22,819	220	354	600	0	0.66 (0.64–0.69)	0.73 (0.7–0.76)	0.63 (0.6–0.66)	0.99 (0.99–0.99)	6
Ibuprofen	No	21,259	254	2,123	357	0	0.2 (0.17–0.23)	0.58 (0.54–0.62)	0.14 (0.13–0.16)	0.99 (0.99–0.99)	12
Indometacin	Yes	23,617	101	88	187	0	0.66 (0.61–0.71)	0.65 (0.59–0.7)	0.68 (0.62–0.73)	1 (0.99–1)	6
Ketoprofen	Yes	23,684	29	74	206	0	0.8 (0.76–0.84)	0.88 (0.83–0.92)	0.74 (0.68–0.79)	1 (1–1)	3
Meloxicam	Yes	21,186	628	507	1,672	0	0.72 (0.7–0.74)	0.73 (0.71–0.75)	0.77 (0.75–0.78)	0.97 (0.97–0.97)	6
Naproxen	No	22,312	309	340	1,032	0	0.75 (0.73–0.77)	0.77 (0.75–0.79)	0.75 (0.73–0.77)	0.99 (0.98–0.99)	6
Paracetamol	No	12,382	1,144	8,178	2,289	0	0.15 (0.13–0.16)	0.67 (0.65–0.68)	0.22 (0.21–0.23)	0.92 (0.91–0.92)	12
Piroxicam	Yes	23,665	78	58	192	0	0.74 (0.69–0.78)	0.71 (0.65–0.76)	0.77 (0.71–0.82)	1 (1–1)	12
Class: glucocorticoid											
Prednisolone/prednisone	Yes	16,288	1,265	1,445	4,856	139	0.71 (0.69–0.72)	0.79 (0.78–0.8)	0.77 (0.76–0.78)	0.93 (0.92–0.93)	3

* b/tsDMARD, biologic or targeted synthetic disease‐modifying antirheumatic drug; CI, confidence interval; csDMARD, conventional synthetic disease‐modifying antirheumatic drug; FN, false negative; FP, false positive; N/A, “Don't know” response in nonbinary question; NPV, negative predictive value; PBS, Pharmaceutical Benefits Scheme; PPV, positive predictive value; TN, true negative; TP, true positive.

^a^ Window of maximal agreement (kappa). Where two windows had the same kappa, the window with the highest sensitivity was selected.

Using the optimal look‐back window for each medication, convergent validity, measured by the agreement of prescription claims data with self‐report, was high for b/tsDMARDs (κ 0.83–0.95, with the exception of rituximab, which was substantial [κ 0.63, 95% CI 0.6–0.67]), substantial to high for csDMARDs (κ 0.67–0.86), substantial for prescription‐only nonopioid analgesics (κ 0.66–0.80), substantial for oral prednisolone/prednisone (κ 0.71, 95% CI 0.69–0.72), and moderate to substantial for prescription‐only opioid analgesics (κ 0.48–0.7). NPVs were very high across all drug classes (≥0.92). Self‐reports of typically OTC medicines showed poorer agreement with prescription claims (κ 0.15–0.75).

### Effect of look‐back window length on agreement parameters

Figure 2 demonstrates that using retrospective claims data look‐back windows of increasing length generally increases PPV toward a plateau, at the expense of reducing sensitivity, by creating a less accurate but more sensitive reference standard. Sensitivity also varied according to drug class, appearing generally lower for OTC and irregularly dispensed medicines (ie, analgesics and prednisolone/prednisone). The look‐back window of maximal agreement (kappa) can be approximated for each medication, acknowledging that CIs overlap for several comparisons. Rituximab, oral methotrexate, piroxicam, ibuprofen, aspirin, and paracetamol were exceptions, with no obvious point of maximal agreement within the scope of this analysis.

### Predictors of discordant self‐report

Unadjusted univariate analyses highlighted several variables of potential significance as predictors of discordant self‐report (Supplementary Table S8). When combined into multivariable logistic regression models, no broadly consistent predictive factors were found across all prescription medications; however, several statistically significant unidirectional and bidirectional associations were noted across multiple medications (Table 3).

**Table 3:** Multivariate ORs (95% CIs) for patient questionnaire factors associated with discordant self‐reported prescription medication use (false positive or false negative) using Australian PBS prescription claims data as the reference standard*

Medication	Questionnaire variable											
	Married^a^	Age^b^	Higher education^c^	Disease duration^d^	Current smoker^e^	Socioeconomic status^f^	Female sex^g^	Online questionnaire^h^	Currently depressed or anxious^i^	Current pain^j^	Self‐rated health^k^	HAQ score^l^
Class: csDMARD												
Hydroxychloroquine	0.98 (0.83–1.16)	1 (1–1.01)	0.94 (0.8–1.11)	0.98 (0.97–0.99)^m^	1.03 (0.76–1.36)	1 (1–1)	1.62 (1.35–1.96)^m^	1.44 (1.2–1.74)^m^	1.18 (1.01–1.39)	1.45 (1.1–1.96)^m^	0.99 (0.99–1)^m^	1.2 (1.05–1.36)^m^
Leflunomide	0.75 (0.63–0.88)^m^	1.01 (1–1.01)	0.94 (0.79–1.13)	0.98 (0.97–0.99)^m^	1.59 (1.22–2.04)^m^	1 (1–1.01)	1.01 (0.85–1.21)	0.88 (0.73–1.05)	1.28 (1.08–1.52)^m^	0.93 (0.71–1.24)	0.99 (0.99–0.99)^m^	1.23 (1.08–1.41)^m^
Methotrexate (injection)	0.8 (0.64–1.02)	1 (0.99–1.01)	1.13 (0.89–1.44)	0.97 (0.96–0.99)^m^	0.73 (0.44–1.14)	1 (1–1.01)	0.83 (0.65–1.05)	1.68 (1.28–2.24)^m^	1.51 (1.19–1.9)^m^	0.83 (0.58–1.21)	0.99 (0.99–1)^m^	1.53 (1.27–1.84)^m^
Methotrexate (oral)	0.83 (0.76–0.91)^m^	1 (1–1.01)	0.98 (0.89–1.07)	0.99 (0.99–0.99)^m^	1.14 (0.96–1.34)	1 (1–1)	1.29 (1.16–1.42)^m^	1.11 (1–1.24)	1.08 (0.99–1.19)	1.03 (0.9–1.19)	1 (0.99–1)^m^	1.1 (1.02–1.19)^m^
Sulfasalazine	1.31 (1.08–1.6)^m^	1 (0.99–1)	0.94 (0.78–1.14)	0.98 (0.97–0.99)^m^	0.95 (0.67–1.3)	1 (1–1)	0.87 (0.72–1.05)	1.04 (0.86–1.28)	1.13 (0.94–1.36)	1.15 (0.86–1.57)	1 (0.99–1)	1.41 (1.21–1.63)^m^
Class: b/tsDMARD												
Abatacept (injection)	0.89 (0.66–1.21)	1.02 (1.01–1.04)^m^	1.03 (0.76–1.39)	0.99 (0.98–1.01)	0.81 (0.42–1.4)	0.99 (0.99–1)^m^	1.91 (1.35–2.76)^m^	1.63 (1.16–2.29)^m^	0.99 (0.74–1.33)	1.52 (0.87–2.87)	0.98 (0.97–0.99)^m^	1.06 (0.84–1.33)
Adalimumab	1.02 (0.84–1.23)	0.99 (0.98–0.99)^m^	0.94 (0.78–1.13)	1 (0.99–1.01)	1.72 (1.3–2.25)^m^	1 (1–1.01)	0.76 (0.64–0.91)^m^	2.04 (1.63–2.59)^m^	0.91 (0.75–1.09)	1.26 (0.97–1.64)	0.99 (0.99–1)^m^	0.88 (0.75–1.03)
Etanercept	0.91 (0.75–1.12)	1 (0.99–1.01)	1.01 (0.83–1.22)	0.97 (0.96–0.98)^m^	1.34 (0.96–1.81)	1 (1–1)	1.12 (0.92–1.37)	1.89 (1.5–2.41)^m^	0.8 (0.65–0.97)	1.01 (0.77–1.33)	1 (1–1.01)	1.23 (1.05–1.45)^m^
Golimumab	1.03 (0.76–1.41)	0.99 (0.98–1)	0.81 (0.6–1.09)	1 (0.99–1.01)	0.89 (0.48–1.51)	1 (0.99–1)	0.73 (0.55–0.99)	2.66 (1.81–4.01)^m^	0.99 (0.73–1.34)	0.92 (0.61–1.42)	0.99 (0.99–1)	1.11 (0.86–1.42)
Rituximab	0.91 (0.73–1.15)	1 (0.99–1.01)	0.74 (0.59–0.94)^m^	1 (0.99–1.01)	1.12 (0.75–1.62)	1 (0.99–1)	1.35 (1.06–1.74)	1.12 (0.88–1.43)	1.18 (0.94–1.47)	1.23 (0.85–1.82)	1.01 (1–1.01)	1.43 (1.2–1.71)^m^
Tocilizumab	0.95 (0.72–1.28)	1 (0.99–1.01)	1.25 (0.93–1.68)	0.98 (0.97–0.99)^m^	2.34 (1.58–3.38)^m^	1 (0.99–1)	1.66 (1.19–2.37)^m^	2.29 (1.61–3.32)^m^	0.94 (0.71–1.26)	1.74 (0.98–3.37)	0.98 (0.98–0.99)^m^	1.52 (1.22–1.9)^m^
Class: opioid analgesic												
Oxycodone	1.01 (0.88–1.16)	0.99 (0.99–1)^m^	1.01 (0.87–1.16)	1 (1–1.01)	1.05 (0.82–1.33)	1 (1–1)	0.92 (0.8–1.06)	0.88 (0.76–1.01)	1.05 (0.92–1.2)	1.92 (1.41–2.68)^m^	0.99 (0.99–0.99)^m^	2.08 (1.87–2.31)^m^
Tramadol	0.96 (0.8–1.15)	0.99 (0.98–1)^m^	1.1 (0.91–1.32)	1 (0.99–1.01)	1.17 (0.86–1.57)	1 (0.99–1)	1.39 (1.14–1.71)^m^	0.86 (0.71–1.04)	0.88 (0.74–1.05)	3.95 (2.42–6.99)^m^	0.99 (0.98–0.99)^m^	1.62 (1.42–1.86)^m^
Celecoxib	1.07 (0.92–1.26)	1 (0.99–1.01)	1.17 (1–1.36)	0.98 (0.98–0.99)^m^	1.19 (0.91–1.53)	1 (1–1.01)^m^	1.16 (0.99–1.36)	0.98 (0.83–1.16)	1.08 (0.93–1.25)	1.74 (1.36–2.25)^m^	1 (0.99–1)	1.03 (0.91–1.16)
Indometacin	1.19 (0.84–1.71)	0.99 (0.98–1)	1.11 (0.78–1.57)	1.02 (1–1.03)^m^	1.04 (0.54–1.82)	1 (1–1.01)	0.71 (0.51–1)	0.66 (0.47–0.95)	1.54 (1.1–2.14)^m^	2.07 (1.15–4.06)	1.01 (1–1.02)	1.25 (0.95–1.63)
Meloxicam	1 (0.87–1.16)	1 (0.99–1.01)	0.83 (0.72–0.96)^m^	0.99 (0.98–1)^m^	1.02 (0.79–1.29)	1 (1–1)	1.44 (1.24–1.67)^m^	1.05 (0.9–1.22)	1.23 (1.07–1.41)^m^	1.37 (1.1–1.73)^m^	1 (0.99–1)^m^	0.91 (0.81–1.02)
Class: glucocorticoid												
Prednisolone/prednisone	1.08 (0.98–1.19)	1 (0.99–1)	1.05 (0.95–1.15)	0.99 (0.99–1)^m^	1.28 (1.1–1.5)^m^	1 (1–1)	1.15 (1.05–1.27)^m^	0.95 (0.86–1.04)	0.99 (0.91–1.09)	1.52 (1.3–1.79)^m^	0.99 (0.99–1)^m^	1.24 (1.15–1.33)^m^

* b/tsDMARD, biologic or targeted synthetic disease‐modifying antirheumatic drug; CI, confidence interval; csDMARD, conventional synthetic disease‐modifying antirheumatic drug; OR, odds ratio; PBS, Pharmaceutical Benefits Scheme.

^a^ Married vs not married (control).

^b^ Age (years, continuous).

^c^ Tertiary qualified vs no tertiary qualification (control).

^d^ Disease duration (years, continuous).

^e^ Current smoker vs nonsmoker (control).

^f^ Index of Relative Socio‐economic Advantage and Disadvantage Socio‐Economic Indexes for Areas (SEIFA) by Statistical Area Level 1 2011 percentile (continuous).

^g^ Female vs male sex (control).

^h^ Questionnaire completed online vs on paper (control).

^i^ Current depression or anxiety (EuroQol 5‐dimension 3‐level [EQ‐5D‐3L] level 2 or 3) vs none (EQ‐5D‐3L level 1; control).

^j^ Current pain (EQ‐5D‐3L level 2 or 3) vs none (EQ‐5D‐3L level 1; control).

^k^ Self‐rated health (EQ‐5D‐3L visual analog scale health state [0, worst imaginable health state, to 100, best imaginable health state]; continuous).

^l^ HAQ disability index score (0, mild disability, to 3, very severe disability; continuous).

^m^ False discovery rate–adjusted *P* < 0.05.

Unidirectional OR associations with discordant self‐report were (1) self‐reported disability severity (HAQ score out of 3) for 11 medications (OR range 1.1–2.08), (2) self‐rated health (EQ‐5D VAS out of 100) for 11 medications (OR range 0.98–1), (3) online questionnaire modality for 7 medications (OR range 1.44–2.66; ES very small to small), (4) current moderate‐severe pain for 6 medications (OR range 1.37–3.95; ES very small to medium), (5) current smoking status for 4 medications (OR range 1.28–2.34; ES very small to small), (6) current moderate‐severe depression or anxiety for 4 medications (OR range 1.23–1.54; ES very small), and (7) higher education for meloxicam and rituximab (OR range 0.74–0.83; ES very small).

Bidirectional OR associations were (1) shorter disease duration (years) for 10 medications (OR range 0.97–0.99), with the exception of indometacin (OR 1.02, 95% CI 1–1.03); (2) female sex for 7 medications (OR range 1.15–1.91; ES very small to small), with the exception of adalimumab (OR 0.76, 95% CI 0.64–0.91; ES very small); (3) being married for leflunomide and methotrexate (oral) (OR range 0.75–0.83; ES very small), with the exception of sulfasalazine (OR 1.31, 95% CI 1.08–1.6; ES very small); (4) younger age (years) for adalimumab, oxycodone, and tramadol (OR 0.99), with the exception of abatacept injection (OR 1.02, 95% CI 1.01–1.04); and (5) lower SES (IRSAD percentile) for abatacept injection (OR 0.99, 95% CI 0.99–1), with the exception of celecoxib (OR 1, 95% CI 1–1.01).

## DISCUSSION

Our findings demonstrate moderate to high agreement of prescription‐only self‐report with PBS dispensations in this ARAD cohort. No consistent predictors of discordant self‐report were identified across all medications; however, there were several associations of significance. Our data suggest poorer physical function and worse overall health may influence reliability of self‐reported medication data to some extent.

A conventional three‐month look‐back period of claims data was optimal for the majority of medications; however, those typified by longer dosing intervals (eg, rituximab, ustekinumab, infliximab), intermittent use (eg, analgesics, anti‐inflammatories), or potentially larger dispensing quantities relative to dose requirements (eg, oral methotrexate, hydroxychloroquine, sulfasalazine) typically required wider retrospective look‐back windows of claims data (6–12 months) to optimize agreement. Although not done in this analysis, a logical next step to establishing the optimal look‐back window for each medication would be to attempt to anchor each medication look‐back period to its typical dosing frequency. This approach would most likely lead to a higher level of optimization compared with the four time windows we applied across all the medications analyzed.

The very high kappa and sensitivity values for the majority of the b/tsDMARD medication class (up to κ = 0.95 and sensitivity = 0.96 for infliximab) are perhaps the best indications of the true validity of ARAD participants’ self‐reports; the strong financial incentive to access these high‐cost drugs heavily subsidized through the PBS meant claims data were likely to accurately reflect almost all b/tsDMARD dispensing in this cohort. An outlier in this class was rituximab; its lower kappa value (0.63, 95% CI 0.6–0.67) likely reflected its long dosing intervals (typically every six months or longer in RA) and higher rates of in‐hospital administration funded by state public health services (non‐PBS).

Conversely, many analgesics, anti‐inflammatories, and glucocorticoids were priced below the PBS copayment threshold during the study period and were available in generic forms priced equivalently or cheaper, when accessed by private prescriptions, than the discounted or concession PBS price. Therefore, there was likely to be a substantially larger private market not captured in the claims data for cheaper medicines, contributing to expectedly lower concordance measures unrelated to participants’ adherence and accuracy with self‐reporting. Alternative methods of validation are better suited for these medication classes, such as a patient diary/interviews and/or the “brown bag” method or drug assays on biological samples.^16^

Although source data and methodologies for self‐report medication validation vary,^13^ our results are consistent with other studies, suggesting medications taken on a long‐term basis or for serious health conditions (eg, DMARDs) show better agreement with other sources than intermittent or general‐use medications (eg, glucocorticoids, anti‐inflammatories, and analgesics).^11^, ^40^, ^41^, ^42^ Solomon et al reported moderate to excellent validity of current self‐report of RA medications from 91 participants in the Brigham Rheumatoid Arthritis Sequential Study questionnaire, which used medical record data as the reference standard.^20^ Compared with our claims (PBS) data analysis, they showed higher agreement for self‐report of hydroxychloroquine, methotrexate, leflunomide, and etanercept use but lower agreement for sulfasalazine and infliximab, with wider CIs.

Although we did not identify studies of predictors of discordant self‐reported medications in similar cohorts, our findings are congruent with a 2021 systematic review of cancer‐related studies that found no consistent associations with age, education, or marital status.^13^ The increased odds of discordance with poorer self‐reported health status is also consistent with previous studies in older adults (brown bag method self‐reports vs pharmacy claims) and women with breast cancer (interview‐derived self‐reports vs medical records).^8^, ^43^

This is the first Australian study to compare accuracy of self‐reported rheumatology medication use by adults and children with inflammatory arthritis to pharmaceutical claims data across a range of medications. It adds to the existing literature with a nuanced focus on antirheumatic medication classes in a disease‐specific cohort, where medications were self‐reported via longitudinal self‐administered paper and electronic questionnaires over an 11‐year period. Although the large sample size led to good statistical precision, the small number of JIA cases (n = 96) may impact applicability of our findings to this subgroup.

A key strength of this study is the identification of optimal look‐back windows of claims data required for validating self‐reported use of multiple rheumatology‐related medications in an Australian context. Previous studies have most commonly used a three‐month window of claims data to validate self‐report.^11^, ^42^, ^44^, ^45^, ^46^ Our analysis suggests this approach may underestimate agreement for select rheumatology medicines.

Question labeling and structure can affect recall of self‐reported drug use, and drug name recognition is an important factor in accurate patient self‐report.^7^, ^47^ We minimized effects of underreporting related to missing drug trade name prompts by including a free‐text field analysis to identify mismatched medication status reports with free‐text current medication responses.

Health literacy has been shown to be an influential factor for understanding medication regimens and self‐reporting them accurately.^48^ Our understanding of health literacy across the full ARAD cohort was limited; however, data from a subset (n = 994)^49^ that used the Single‐Item Literacy Screener^50^ demonstrated high health literacy, with only 2% “always/often” needing assistance reading medical information.

As others have described, using dispensing data as a reference standard is imperfect because dispensed medications may not reflect actual use.^17^, ^35^, ^44^, ^51^ Additionally, our exclusion criteria had to be carefully designed to ensure self‐reports were not inappropriately classified as false positives due to historical changes and updates to medication listings on the PBS. In Australia, most PBS medicines are supplied with enough quantity for approximately one to two months of treatment; however, rare circumstances arise in which larger amounts may be dispensed, such as extended overseas travel or for those living in remote locations. These and other factors, such as children being listed under more than one Medicare number, missing data for veterans’ medicines dispensed under the Repatriation Pharmaceutical Benefits Scheme, documentation errors by pharmacists or prescribers, and temporal variation in PBS data capture, could also have elevated the false positive rate and led to lower‐than‐expected agreement for pertinent medications.

This study had several limitations. First, our analytical approach treated each questionnaire as an independent unit of analysis rather than clustering at the patient level. Although this allowed us to maximize the use of available data and capture the changing real‐world circumstances reflected in each response, it also introduced certain limitations. Intraindividual variation in recall ability is likely to be substantially lower than interindividual variation and may correlate with other factors influencing discordance, such as medication adherence or follow‐up duration. Consequently, patients who submitted more questionnaires during the analysis period may have exerted a disproportionately large influence on the overall estimates, potentially skewing validation metrics and regression analyses, for example, by falsely increasing precision.^52^ Moreover, because most studies of self‐reported medication use adopt a patient‐level analytical framework, our questionnaire‐level approach may limit the generalizability of our findings to other settings. Nevertheless, we believe this method provides internally valid estimates and highlights important dynamics that would be obscured in a purely patient‐level analysis. Future work could build on this by modeling intraindividual trajectories of agreement over time.

Second, only 68% of ARAD participants provided the required consent for linkage to administrative health datasets, which may have introduced selection bias. Third, some bias may have arisen from prefilling previous responses in participants’ longitudinal questionnaires, potentially reducing generalizability. Fourth, we limited our predictor analysis to prescription‐only medications that met a minimum usage threshold in our cohort, thereby reducing the number of responses included in the analysis. Fifth, csDMARD, glucocorticoid, and analgesic medication questions did not include a “date stopped” field, making it impossible for participants to report when these medications were ceased between questionnaire intervals. This likely contributed somewhat to the generally lower agreement observed for these medication classes when compared with the b/tsDMARDs.

Investigators seeking to validate medication self‐report with prescription claims data should be mindful of individual medication prescribing, dispensing, and use profiles when defining validation parameters. Wider retrospective look‐back windows of claims data may help to optimize agreement with self‐report for prescription medications characterized by longer dosing intervals, intermittent use, and larger quantity dispensations.

Despite challenges in data collection, self‐reporting remains valuable for ensuring medication use data completeness in cohort studies, given limitations of current reference sources. Participants with significant disability and/or poor health status may need additional support to maximize self‐report accuracy. Future efforts should focus on how to efficiently combine these data sources to optimize accuracy and reduce self‐report burden, for example, by linking claims data records with electronic questionnaire platforms to assist recall while gathering crucial subjective experience data.

Acknowledging PBS prescription claims data as an imperfect reference standard, our findings suggest ARAD participants accurately report current antirheumatic medication use via longitudinal self‐reported questionnaires. Although no consistent predictors of discordant self‐ report were found to generalize across all medications, worse self‐rated measures of disability severity and overall health status were most consistently associated with discordance.

## AUTHOR CONTRIBUTIONS

All authors contributed to at least one of the following manuscript preparation roles: conceptualization AND/OR methodology, software, investigation, formal analysis, data curation, visualization, and validation AND drafting or reviewing/editing the final draft. As corresponding author, Dr Lynch confirms that all authors have provided the final approval of the version to be published, and takes responsibility for the affirmations regarding article submission (eg, not under consideration by another journal), the integrity of the data presented, and the statements regarding compliance with institutional review board/Declaration of Helsinki requirements.

## Supporting information


**Disclosure form**.

**Appendix S1:** Supplementary Information
