# Supplementary material for: Agreement Between Self‐Reported Antirheumatic Medications and Pharmaceutical Claims in an Australian Inflammatory Arthritis Cohort
Source: ACR Open Rheumatol. 2025 Oct 17;7(10):e70105. doi: 10.1002/acr2.70105 (PMC12532489; doi:10.1002/acr2.70105)
Supplement: Supplementary file 2 — Appendix S1: Supplementary Information [file ACR2-7-e70105-s001.docx]

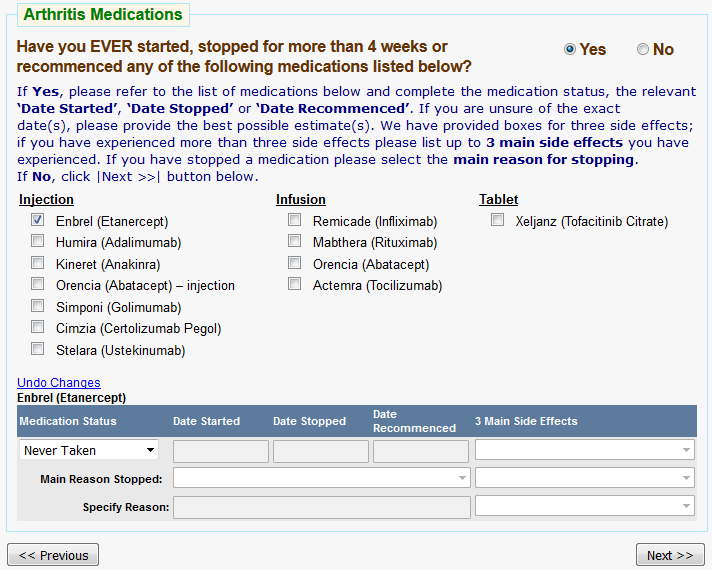


# Supplementary Figure S1a. Question formatting used for biologic and targeted synthetic disease modifying drug (b/tsDMARD) self-reports in the baseline online ARAD questionnaires.


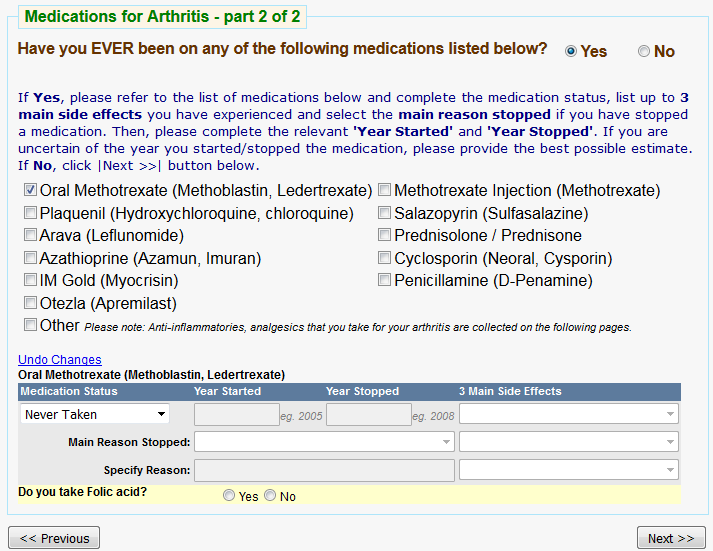


# Supplementary Figure S1b. Question formatting used for conventional synthetic disease modifying drug (csDMARD) and oral glucocorticoid self-reports in the baseline online ARAD questionnaires.


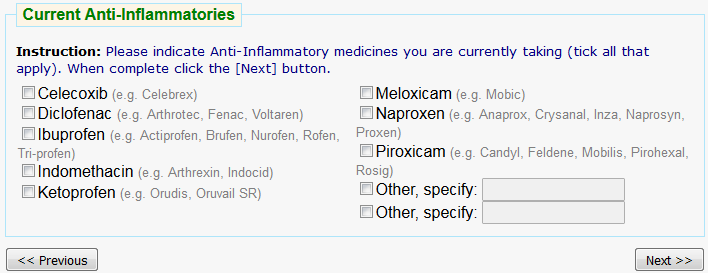


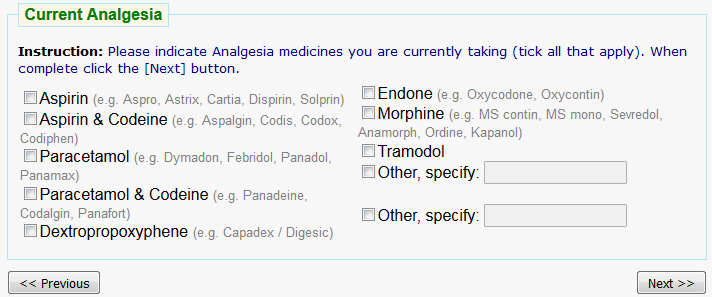


# Supplementary Figure S1c. Question formatting used for anti-inflammatories and analgesic self-reports in the baseline online ARAD questionnaires.

# Supplementary Table S1. ARAD self-reported medications and analysis considerations

| **Medication name** | **Trade name/s^*^** | **Full class** | **Prescription-only** | **Form** | **Question format** | **Typical dosing frequency** | **PBS listing dates^†^** | **ATC codes** | **PBS item codes** | **Included in validation analysis** | **Included in predictor analysis** |
| --- | --- | --- | --- | --- | --- | --- | --- | --- | --- | --- | --- |
| Abatacept - infusion | Abatacept | Immunosuppressant - b/tsDMARD - T-cell co-stimulation modulator | Yes | IV | dropdown | Induction (0, 2 and 4 weeks) then maintenance every 4 weeks | Available since December 2007 for RA | L 04 A A 24 | 05605B, 09621J, 12738K, 12756J | Yes | No |
| Abatacept - injection | Abatacept | Immunosuppressant - b/tsDMARD - T-cell co-stimulation modulator | Yes | SC | dropdown | Once a week | Available since April 2012 for RA | L 04 A A 24 | 01220F, 01221G, 11068M, 11092T, 11684Y, 11693K, 12680J, 12758L, 13034B, 13037E | Yes | No |
| Adalimumab | Humira | Immunosuppressant - b/tsDMARD - TNF inhibitor | Yes | SC | dropdown | Every 1 or 2 weeks | Available since May 2004 for RA | L 04 A B 04 |  | Yes | Yes |
| Anakinra | Kineret | Immunosuppressant - b/tsDMARD - Interleukin 1 receptor antagonist | Yes | SC | dropdown | Daily | Listed December 2004 for RA, Discontinued on PBS for RA and JIA in December 2010 | L 04 A C 03 |  | No | No |
| Apremilast | Apremilast | Immunosuppressant - tsDMARD | Yes | PO | dropdown | Daily | Available since January 2021 for psoriasis | L 04 A A 32 |  | No | No |
| Aspirin | Aspro, Astrix, Cartia, Dispirin, Solprin | Analgesic / antiplatelet - Non-opioid | No | PO | checkbox | PRN | Available since at least 2003 | B 01 A C 06, B 01 A C 30, N 02 B A 01 |  | Yes | No |
| Aspirin & codeine | Aspalgin, Codis, Codox, Codiphen | Analgesic - Opioid | No | PO | checkbox | PRN | Available since at least 2003 | N 02 A A 59, N 02 A J 07, N 02 B A 51 | 01222H, 03315L, 04061R, 04062T, 04286N, 06031K | Yes | No |
| Azathioprine | Azamun, Imuran | Immunosuppressant - csDMARD | Yes | PO | dropdown | Daily | Available since at least 2003 | L 04 A X 01 |  | Yes | No |
| Celecoxib | Celebrex | Anti-inflammatory - NSAID | Yes | PO | checkbox | PRN | Available since at least 2003 | M 01 A H 01 |  | Yes | Yes |
| Certolizumab pegol | Cimzia | Immunosuppressant - b/tsDMARD - TNF inhibitor | Yes | SC | dropdown | Every 2 or 4 weeks | Available since August 2010 for RA | L 04 A B 05 |  | Yes | Yes |
| Cyclosporin | Neoral, Cysporin | Immunosuppressant - csDMARD | Yes | PO | dropdown | Daily | Available since at least 2003 for RA | L 04 A D 01, L 04 A A 01, S 01 X A 18 |  | Yes | No |
| Dextropropoxyphene | Capadex, Digesic | Analgesic - Opioid | Yes | PO | checkbox | PRN | Listed from 2003; discontinued on PBS April 2012 | N 02 A C 04 |  | No | No |
| Diclofenac | Arthrotec, Fenac, Voltaren | Anti-inflammatory - NSAID | No | PO, TOP | checkbox | PRN | Available since at least 2003 | M 01 A B 05, D 11 A X 18, S 01 B C 03 |  | Yes | No |
| Etanercept | Enbrel | Immunosuppressant - b/tsDMARD - TNF inhibitor | Yes | SC | dropdown | Once or twice a week | Available since February 2002 for RA | L 04 A B 01 |  | Yes | Yes |
| Gold - intramuscular | Myocrisin | Anti-inflammatory / Immunosuppressant - csDMARD | Yes | IM | dropdown | IM injection 1 - 4 weekly | Available since at least 2003; deleted Oct 2019 – Nov 2020 | M 01 C B 01, M 01 C B 04 |  | Yes | No |
| Golimumab | Simponi | Immunosuppressant - b/tsDMARD - TNF inhibitor | Yes | SC | dropdown | Once every four weeks | Available since August 2010 for RA, PsA and AS | L 04 A B 06 |  | Yes | Yes |
| Hydroxychloroquine | Plaquenil | Anti-inflammatory / Immunosuppressant - csDMARD | Yes | PO | dropdown | Daily | Available since at least 2003 for RA | M 01 C A |  | Yes | Yes |
| Ibuprofen | Actiprofen, Brufen, Nurofen, Rofen, Tri-profen | Anti-inflammatory - NSAID | No | PO | checkbox | PRN | Available since at least 2003 | M 01 A E 01 |  | Yes | No |
| Indometacin | Arthrexin, Indocid | Anti-inflammatory - NSAID | Yes | PO | checkbox | PRN | Available since at least 2003 | M 01 A B 01, S 01 B C 01 |  | Yes | Yes |
| Infliximab | Remicade | Immunosuppressant - b/tsDMARD - TNF inhibitor | Yes | IV | dropdown | Induction (0, 2 and 6 weeks), then maintenance every 8 weeks | Available since at least April 2003 for RA | L 04 A B 02, L 04 A A 12 |  | Yes | No |
| Ketoprofen | Orudis, Oruvail SR | Anti-inflammatory - NSAID | Yes | PO | checkbox | PRN | Available since at least 2003 | M 01 A E 03 |  | Yes | No |
| Leflunomide | Arava | Immunosuppressant - csDMARD | Yes | PO | dropdown | Daily | Available since at least 2003 for RA | L 04 A A 13 |  | Yes | Yes |
| Meloxicam | Mobic | Anti-inflammatory - NSAID | Yes | PO | checkbox | PRN | Available since at least 2003 | M 01 A C 06 |  | Yes | Yes |
| Methotrexate - injection | Methotrexate | Immunosuppressant - csDMARD | Yes | SC, IM | dropdown | once weekly | Available since April 2018 | L 01 B A 01, L 04 A X 03 | 01818Q, 02395C, 02396D, 11268C, 11275K, 11283W, 11288D, 11295L, 11544N | Yes | Yes |
| Methotrexate - oral | Methoblastin, Ledertrexate | Immunosuppressant - csDMARD | Yes | PO | dropdown | once weekly | Available since 1980s for RA | L 01 B A 01 | 01622J, 01623K, 02272N | Yes | Yes |
| Morphine | MS Contin, MS Mono, Sevredol, Anamorph, Ordine, Kapanol | Analgesic - Opioid | Yes | PO | checkbox | PRN | Available since at least 2003 | N 02 A A 01 |  | Yes | Yes |
| Naproxen | Anaprox, Crysanal, Inza, Naprosyn, Proxen | Anti-inflammatory - NSAID | No | PO | checkbox | PRN | Available since at least 2003 | M 01 A E 02 |  | Yes | No |
| Oxycodone | Endone, Oxycontin | Analgesic - Opioid | Yes | PO | checkbox | PRN | Available since at least 2003 | N 02 A A 05, N 02 A A 55 |  | Yes | Yes |
| Paracetamol | Dymadon, Febridol, Panadol, Panamax | Analgesic - Non-opioid | No | PO | checkbox | PRN | Available since at least 2003 | N 02 B E 01 |  | Yes | No |
| Paracetamol & codeine | Panadeine, Codalgin, Panafort | Analgesic - Opioid | No | PO | checkbox | PRN | Available since at least 2003 | N 02 A J 06, N 02 A A 59 | 01215Y, 03316M, 04170L, 04171M, 04275B, 08785J, 10186D, 12022R, 12066C, 06032L | Yes | No |
| Penicillamine | D-Penamine | Immunosuppressant - csDMARD | Yes | PO | dropdown | Daily | Available since at least 2003 | M 01 C C 01 |  | Yes | No |
| Piroxicam | Candyl, Feldene, Mobilis, Pirohexal, Rosig | Anti-inflammatory - NSAID | Yes | PO | checkbox | PRN | Available since at least 2003 | M 01 A C 01 |  | Yes | No |
| Prednisolone/Prednisone |  | Immunosuppressant - Glucocorticoid | Yes | PO | dropdown | Daily | Available since at least 2003 | H 02 A B 07, H 02 A B 06 |  | Yes | Yes |
| Rituximab | Mabthera | Immunosuppressants - b/tsDMARD - CD20+ peripheral B-cell depletion | Yes | IV | dropdown | Two doses two weeks apart; repeat at 6 - 12+ months | Available since August 2007 for RA | L 01 X C 02, L 01 F A 01 |  | Yes | Yes |
| Sulfasalazine | Salazopyrin | Anti-inflammatory / Immunosuppressant - csDMARD | Yes | PO | dropdown | Daily | Available since at least 2003 for RA | A 07 E C 01 |  | Yes | Yes |
| Tocilizumab | Actemra | Immunosuppressants - b/tsDMARD - IL-6 inhibitor | Yes | SC, IV | dropdown | Once a week | Available since August 2010 for RA | L 04 A C 07 |  | Yes | Yes |
| Tofacitinib citrate | Xeljanz | Immunosuppressants - tsDMARD - JAK inhibitor | Yes | PO | dropdown | Twice daily | Available since October 2015 for RA | L 04 A A 29 |  | Yes | No |
| Tramadol |  | Analgesic - Opioid | Yes | PO | checkbox | PRN | Available since at least 2003 | N 02 A X 02 |  | Yes | Yes |
| Ustekinumab | Stelara | Immunosuppressants - b/tsDMARD - IL12/-23 inhibitor | Yes | SC | dropdown | Induction (0 and 4 weeks), then maintenance every 12 weeks | Available since March 2010 for psoriasis | L 04 A C 05 |  | Yes | No |
| ^*^As listed in ARAD questionnaire v7.0 20/11/2015; ^†^Publication records available from 2003 onwards; | | | | | | | | | | | |
| Abbreviations: ARAD: Australian Rheumatology Association Database; ATC: Anatomical Therapeutic Chemical Classification; IL: Interleukin; IM: Intramuscular ; IV: Intravenous ; JAK: Janus Kinase; JIA: Juvenile Idiopathic Arthritis; NSAID: Non-Steroidal Anti-Inflammatory Drug ; PBS: Pharmaceutical Benefits Scheme; PO: Per Os (Oral); PRN: Pro Re Nata (As Needed); PsA: Psoriatic Arthritis; RA: Rheumatoid Arthritis; SC: Subcutaneous; TNF: Tumour Necrosis Factor; TOP: Topical | | | | | | | | | | | |

# Supplementary Table S2. Results of free text analysis of self-reported medication fields with recoding

| **Medication name** | **String match criteria** | **Matching free text responses** | **Number of recoded mismatches** |
| --- | --- | --- | --- |
| Tocilizumab | (?i)actemra\|toci | actemra\|actemra\|actemra\|actemra\|actemra\|Actemra\|Actemra\|Actemra\|Tocilizumab Humira Trapentadol 50mg - only when back pain very bad\|Actemra\|Actemra\|Actemra Panadol osteo Paradise forte\|ACTEMRA INJECTION\|ACTEMRA INJECTION\|actemra\|actemra\|actemra\|actemra\|Actemra | 19 |
| Leflunomide | (?i)arav\|leflun | Arava\|arava\|Arava\|leflunomide\|Leflunomide lyrica\|Arava\|arava, prednisone, enbrel\|Prednisone Methotrexate and Arava\|arava\|arava lefluonimide restarted in 2022 voltaren cream\|taltz Ixekizumab Leflunomide\|ARAVA\|Arava\|Arava\|Leflunomide\|Arava\|Leflunomide\|Leflunomide 20mg\|Enbrel Arava\|Leflunomide\|Arava | 7 |
| Certolizumab pegol | (?i)cimzia | HUMIRA cimzia\|HUMIRA cimzia\|cimzia\|Cimzia\|Cimzia\|Cimzia\|Cimzia\|CIMZIA | 3 |
| Etanercept | (?i)brenz\|enb\|embrel\|Etanercept\|embral\|benepali | enbrel\|BRENZYS CONSENTYX\|brenzys\|Enbrel\|Enbrel\|brenzys\|brenzys\|brenzys\|Enbrel\|brenzys\|brenzys\|brenzys\|brenzys\|brenzys\|enbrel injection\|Enbrel 50 mg weekly\|enbrel inj\|benepali\|BRENZYS\|brenzys patch 20\|brenzys morph patches\|Embrel Methotraxtate\|Enbrel injections\|Enbrel\|Enbrel injections\|Enbrel\|BRENZYS\|arava, prednisone, enbrel\|Enberal none\|Enbrel\|Enbrel Targin\|Enbrel Targin\|Enbrel\|Enbrel\|Prednisolone during Enbrel trial Somac due to Celebrex side effects\|Enbrel\|embrel\|Etanercept Sulphasalazine\|Etanercept\|enbrel\|Enbrel\|oxycontin-nortriptyline-lyrica-prednisone-enbrel injections-methotrexate-folic acid- prednisone enbrel\|oxycontin-nortriptyline-lyrica-prednisone-enbrel injections-methotrexate-folic acid-\|oxycontin-nortriptyline-lyrica-prednisone-enbrel injections-methotrexate-folic acid-\|oxycontin-nortriptyline-lyrica-prednisone-enbrel injections-methotrexate-folic acid-\|brenzys\|brenzys\|brenzys\|brenzys\|embral targin\|Enbrel Arava\|Enbrel\|Brenzy\|brenzys Prednisolone when needed\|brenzys\|brenzys\|brenzys\|brenzys\|Enbrel\|Enbrel\|BRENZYS\|BRENZYS\|Enbrel\|Enbrel\|Enbrel\|Enbrel\|Enbrel\|brenzys\|Brenzys\|Brenzys\|Brenzys\|Brenzys\|Brenzys\|Sulfasalazine embrel Panadeine forte\|Hydroxychloroquine/sulfasalazine/embrel\|brenzys\|brenzys\|brenzys\|brenzys | 50 |
| Gold - intramuscular | (?i)gold 50 injection | gold 50 injection norspan patch\|gold 50 injection\|gold 50 injection | 3 |
| Adalimumab | (?i)hum | HUMIRA\|HUMIRA cosentyx 150mg\|HUMIRA cimzia\|HUMIRA\|HUMIRA cimzia\|HUMIRA\|HUMIRA\|Humira\|humira\|humira\|humira\|humira\|Humira\|Humira\|Humira\|humira\|Humira\|Humira\|Humira\|Humira 40mg self injection\|Humira 40mg/14 days\|humira\|humira\|HUMIRA\|Tocilizumab Humira Trapentadol 50mg - only when back pain very bad\|Humira\|humira simponi (2016) cosentyx (2020)\|Humira\|Humira\|Humira\|Humira\|Humira\|Humira\|Humira\|Humira\|Humira\|humira\|Humira Palexia Sr Palexia IR\|Humira\|Humira\|Humira injection\|Humira\|Humira\|Humira\|Humira | 25 |
| Methotrexate - injection | (?i)methotrexate inj | methotrexate inj | 0 |
| Methotrexate - oral | (?i)methotrex\|methotrax\|mtx\|Methyltrexate | methotrexate\|Methotrexate Plaquenil Panadol Oster\|Oral Methotrexate\|Oral Methotrexate\|Oral Methotrexate\|Oral Methotrexate\|mtx\|mtx\|mtx pro cid\|Methotrexate\|Methotrexate\|Methotrexate\|Embrel Methotraxtate\|Methotraxae\|methotrexate inj\|Prednisome Methyltrexate Targin\|Paracetemol Methotrexate\|Prednisone Methotrexate Panadol Osteo\|Prednisone Methotrexate and Arava\|Prednisone Methotrexate Targin Panadol osteo\|oxycontin-nortriptyline-lyrica-prednisone-enbrel injections-methotrexate-folic acid- prednisone enbrel\|oxycontin-nortriptyline-lyrica-prednisone-enbrel injections-methotrexate-folic acid-\|oxycontin-nortriptyline-lyrica-prednisone-enbrel injections-methotrexate-folic acid-\|oxycontin-nortriptyline-lyrica-prednisone-enbrel injections-methotrexate-folic acid-\|Mobic Methotrexate Salazopyrin\|Methotrexate Hydroxychloroquin\|Methotrexate tablets Prednisone Panadol Osteo | 9 |
| Apremilast | (?i)aprem\|otez\|Ozetel\|Otzela\|aprimast | APREMILAST (TRIAL)\|APREMILAST (TRIAL)\|APREMILAST (TRIAL)\|APREMILAST (TRIAL)\|APREMILAST (TRIAL)\|APREMALIST PANADOL.OSTEO\|apremalist\|otezla\|aprimast\|otezia otezia\|apremilast\|Otzela\|Ozetel\|4 monthly steriod injections Otezla (Apremilast) | 10 |
| Hydroxychloroquine | (?i)plaq\|Hydroxychloroquine | Methotrexate Plaquenil Panadol Oster\|Plaquenil nurofen\|Sulfasalazine Hydroxychloroquine\|hydroxychloroquine\|Plaquinil\|Plaquenil\|Panadol osteo Pregabalin Targin - on occasions Plaquenil\|Hydroxychloroquine\|plaquenil tofacitinib\|Plaquenill Neurofen, Advil\|Plaquenill\|Prednisolone plaquenil\|Plaquenil\|Hydroxychloroquine/sulfasalazine/embrel | 8 |
| Prednisolone/Prednisone | (?i)pred\|Panafcortelone\|prendis\|perdnisone\|panfortcortelone\|prdinisolone | prednisolone\|prednisolone\|Prednisone\|prednisonol\|cosentyx Prednislone\|Prednisone\|Prednisilone Norspan Pain Patch Lyrica & Endep\|Prednisolone\|Sulfasalazine Prednisone\|Prednisolone\|prednisoloe\|Prednisolone\|Prednisolone Tumeric Powder\|Prednisolone Tumeric Powder Panadol-Osteo\|predisioone\|Prednisolone\|Prednisolone\|PANAFCORTELONE\|Olumiant - Baricitinib Prednislone osteo panadol\|prednisolone Clindamycin\|Prednisone\|prendisone targin\|prendisone targin\|prednisolne\|prednisone\|Methylprednisolone\|Methylprednisolone\|Methylprednisolone\|Methylprednisolone\|Methylprednisolone\|Methylprednisolone\|Prednisolone\|Prednisone 20 mg\|Prednisolone\|Prednisolone\|Prednisolone Tapentadol\|Prednisolone Steroid injection into spine Tapentadol prn\|Prednisone\|Prednisone injections Internal Morfin Injction\|Prednisone\|Prednisone\|Prednisone\|prednisone\|Prednisone 5mg\|Prednisome Methyltrexate Targin\|Prednisolone\|Prednisolone Osteo-Paracetamol\|arava, prednisone, enbrel\|Prednisone\|prednisone aspirin\|Prednisilone Nurofen Plus\|IV Pulse Dose methylprednisolone infusion\|prdinisolone\|prednisolone\|Prednisolone during Enbrel trial Somac due to Celebrex side effects\|panfortcortelone\|Cortisone injection prednisone\|Prednisolone Celecoxib\|Prednisolone\|prednisone\|prednisone\|Prednisone Methotrexate Panadol Osteo\|Prednisone Methotrexate and Arava\|Prednisone Methotrexate Targin Panadol osteo\|prednisone\|Panafcortelone\|Panafcortelone\|Panafcortelone Simponi\|Prednisolone\|Prednisone\|norspan patches & prednisolone Lyrica\|Prednisone for rash Ibilex for rash\|prednisolone\|Prednisone Salofalk Endep\|Prednisone\|cosenyx prednisolone - as needed panadol osteo\|Prednisone\|perdnisone\|Prednisone\|Prednisone for 4 months Panadol Osteo\|prednilsone\|prednilisone started again\|prednilisone started again\|prednilisone started again\|prednilisone started again\|prednilisone started again\|prednilisone started again apo duloxitine\|Prednisolone\|panafcortelone\|PREDNISOLONE--PRN PANADEINE FORTE\|Prednisolone PRN for flare ups\|Prednisolone Buprenorphine patch\|PREDNISOLONE PRN\|prednisilone\|prednisone,oxycontin,lyrica,etc\|oxycontin prednisone oxycontin lyrica\|oxycontin-nortriptyline-lyrica-prednisone-enbrel injections-methotrexate-folic acid- prednisone enbrel\|oxycontin-nortriptyline-lyrica-prednisone-enbrel injections-methotrexate-folic acid-\|oxycontin-nortriptyline-lyrica-prednisone-enbrel injections-methotrexate-folic acid-\|oxycontin-nortriptyline-lyrica-prednisone-enbrel injections-methotrexate-folic acid-\|Short course prednisone now complete\|Norspan Pyralin, Preds, Methablastin Targin\|prednisolone\|prednisolone\|Prednisolone plaquenil\|Panafcortelone 25mg\|brenzys Prednisolone when needed\|Prednisolone Panadol osteo\|Prednisone\|Prednisolone\|Prednisolone\|Prednisolone 2 1/2 mg\|Prednisolone 2mgm\|prednisolone Panadeine forte\|Prednisolone 5MG Cortisone Injec.Left hand\|prednisolone\|Methotrexate tablets Prednisone Panadol Osteo | 52 |
| Infliximab | (?i)inflectra\|infliximab | infliximab\|Infliximab\|inflectra\|inflectra\|inflectra PARACETAMOL 665mg | 1 |
| Sulfasalazine | (?i)Salaz\|Pyral\|piral\|Sulfasal\|sulph\|slaza | PIRALIN ENEC\|Sulfasalazine Prednisone\|Salazopyrin\|Sulfasalazine Hydroxychloroquine\|sulphasalazine\|Salazapyrn\|Salazopyrin\|Salazopyrin\|Salazopyrin (Sulfasalazine)\|Salazopyrin Mersyndol\|salazopyrin panadol osteo\|Pyralin EN\|PYRALIN\|SULFASALAZINE MESALAZINE Durogesic/FENTANYL\|Pyralin\|salazopyrin, methoblastin\|sulfasalazine\|Salazopyrin\|salazopyrin\|Salazopyrin En 500mg\|Sulfasalazine\|Etanercept Sulphasalazine\|slazapyronel\|sulfasalazine pantoprazole\|pyralin lefllunomide\|Sulfasalazine\|Sulfasalazine\|Sulfasalazine\|Sulfasalazine\|Norspan Pyralin, Preds, Methablastin Targin\|Mobic Methotrexate Salazopyrin\|200 Megafol 5mg 200 Pyralin En 500mg\|Sulfasalazine embrel Panadeine forte\|Hydroxychloroquine/sulfasalazine/embrel | 17 |
| Golimumab | (?i)simp\|sampani | Simponi\|Simponi\|simponi norgestic\|simponi gabapentin norgesic\|Simponi\|simpni\|sampani\|Panafcortelone Simponi\|simponi\|humira simponi (2016) cosentyx (2020)\|Simponi Secukinumab\|Simponi\|Simponi\|Simponi\|Simponi\|Simponi\|Simponi | 13 |
| Ustekinumab | (?i)stel | Stelara UInjection\|Stellara\|Stellara\|Stellara\|Stellara\|Stellara | 5 |
| Tofacitinib citrate | (?i)xel\|tofa\|Xljaanz | Tofacitinib Methodone\|Tofacitinib Methodone\|xeljanz\|xeljanz\|xeljanz\|Xeljanz\|Xeljanz\|Xeljanz\|Xeljanz\|Xeljanz\|Xeljanz\|XELJANZ TOFACITINIB\|Tofacitinib\|XeljNz\|Xeljanz Lyrica\|Tofacitinib citrate\|Xeljanz\|plaquenil tofacitinib\|Xeljanz\|Xeljanz\|Xeljanz. 5mg\|xeljanz\|Tofacitinib\|Tofacitinib\|Tofacitinib\|Tofacitinib\|Tofacitinib\|Tofacitinib\|Tofacitinib\|Xljaanz\|Xeljanz\|Xeljanz\|Xeljanz\|Xeljanz\|Xeljanz\|Xeljanz | 21 |
| Aspirin | (?i)aspirin\|asprin\|aspro | COPIDOGRIL / 1/2 ASPRIN TARGIN\|Low dose aspirin\|Low dose aspirin\|aspro clear\|asprin.atorvastatin,bisoprolol,ticagrelor,ramipril\|asprin.atorvastatin,bisoprolol,ticagrelor,ramipril\|asprin.atorvastatin,bisoprolol,ticagrelor,ramipril\|asprin.atorvastatin,bisoprolol,ticagrelor,ramipril\|Aspirin\|prednisone aspirin\|voltaren gel on joints when needed asprin for headaches not joint pain\|dopridamole /asprin 200/25\|Aspirin\|asprin 400 daily | 6 |
| Celecoxib | (?i)celebrex\|celecoxib | Celebrex\|Celebrex\|Celebrex\|Celebrex\|celebrex 200mg\|celebrex capsules only if I get a flare that I cant control\|celebrex capsules\|celebrex capsules\|celebrex capsules celebrex when I have swollen ankles\|celebrex capsules bacroban,cortic,eleuphrat\|celebrex capsules\|celebrex capsules\|Celebrex\|Prednisolone during Enbrel trial Somac due to Celebrex side effects\|Prednisolone Celecoxib\|Celebrex\|Celebrex\|Celebrex\|Celebrex\|Celecoxib\|Celecoxib\|Celecoxib\|Celecoxib\|Celecoxib\|Celecoxib\|Celecoxib\|Celecoxib Panadol Osteo\|Celebrex 200mg - but only occasionally (when my back goes into spasm)\|Celebrex\|Celebrex\|Celebrex\|Celebrex\|Celebrex | 7 |
| Dextropropoxyphene | (?i)paradex\|capadex\|doloxene | Paradex\|Paradex\|Paradex\|panadol osteo doloxene\|PARADEX | 5 |
| Diclofenac | (?i)dicl\|volt\|vult\|valtaren | Diclofenac rarely taken\|Voltaren Gel\|Voltarin\|Voltaren tabs (rarely take)\|Voltaren\|over the counter valtaren\|Cosentyx x2 Voltaren Gel\|Fish Oil Voltaren gel\|paracetamol voltran 25 mg (occasinally)\|Voltaren 50mg\|Voltaren 25\|Voltarin 12 hourly Gel Panadol Osteo\|voltaren gel on joints when needed asprin for headaches not joint pain\|Voltarin gel\|Voltaren cream\|topical Voltaren emugel\|Voltaren cream on joints\|arava lefluonimide restarted in 2022 voltaren cream\|Diclofenac\|paracetamol voltaren gel\|diclofenic\|diclofenic\|diclofenic\|diclofenic\|diclofenic None\|Vultaren\|voltaren\|voltaren\|voltaren\|voltaren\|Voltaren | 27 |
| Oxycodone | (?i)endone\|oxynorm\|oxi norm | oxi norm norspan patch.10\|endone\|Ibuprofen is for hernia operation Endone & Paracetemol are for hernia operation\|endone & oxycontin\|OXYNORM. TARGIN\|TARGIN OXYNORM\|oxynorm\|oxycontin oxynorm\|None Oxynorm 5mg\|Lyrica, Endep, Only take endone on rare occasions\|Targin 20/10mg Oxynorm 10mg\|Targin 30/15mg Oxynorm 10mg\|Targin Oxynorm\|Targin (oxycodone-nalaxone) Oxynorm (oxycodone)\|"Targin" and "Oxynorm" amitriptyline and duloxetine | 10 |
| Ibuprofen | (?i)ibuprof\|Neurop\|nurofen\|advil\|neurofen\|Neurofin\|Nurophen\|Brufen\|Nurafen | Plaquenil nurofen\|nurofen & codeine nurofen\|nurofen\|Nurofen\|nurofen\|Lyrica Neurofin\|nurofen\|Nurofen\|Ibuprofen is for hernia operation Endone & Paracetemol are for hernia operation\|Turmeric Ibuprofen\|Nurophen Plus\|Nurophen plus\|Nurofen Plus nurofen plus\|Nurophen plus\|Nurofen plus Nurofen plus\|Ibuprofen & codeine\|Nurofen Plus\|Nurofen plus Paracetamol\|Ibuprofen and codeine\|ADVIL\|ADVIL\|ADVIL\|ibuprofen 1 nocte\|Nurofen\|NUROFEN PLUS\|Neurophen\|NUROFEN PLUS\|Ibuprofen\|IBUPROFEN\|Nurafen\|nurofen zavance\|nurofen\|neurofen plus\|advil\|Prednisilone Nurofen Plus\|Ibuprofin\|Advil\|Advil\|Advil\|Advil\|Advil\|Advil\|Advil\|Advil\|Advil\|Advil\|Neurofen Plus\|neurofen\|Neurofen\|nurofen\|Brufen - only as required for inflammation management Panadol - only as required for pain management Codiene - only as required for pain management\|panamax neurofen plus panamax neurofen plus\|nurofen\|Nurofen - ibuprofen\|Nurofen\|Nurofen Zavance\|Neurofin\|Plaquenill Neurofen, Advil\|nurofen\|neurofen\|advil\|Advil\|Advil panadol\|Advil | 48 |
| Indometacin | (?i)indom\|indocid | indocid soppositories\|indocid suppository 100mg when needed\|indocid suppository 100mg when needed\|indocid suppository 100mg when needed\|indocid suppository 100mg when needed\|indocid suppository 100mg when needed\|indocid suppository 100mg when needed\|indocid suppository 100mg when needed\|indocid suppository 100mg when needed\|Omdp Indocid suppositories\|Indocid suppositories\|Indocid Suppositories\|indocid suppositories\|indocid suppositories\|Indocid suppos\|indomethocine | 15 |
| Meloxicam | (?i)Mobic | Mobic\|Mobic only in short bursts Targin 10/5\|MOBIC\|rinvoq Mobic Mersyndol\|mobic\|mobic\|Mobic\|Mobic\|mobic\|mobic\|Mobic\|Mobic\|Mobic\|Mobic Secukinumab 2 pens monthly\|Mobic Secukinumab\|Mobic Consentyx 300 mg\|Mobic Consentyx injection\|Mobic Consentyx\|panadol osteo mobic- rarely\|Mobic\|Mobic\|Mobic\|mobic\|Mobic Endep Lyrica\|Mobic\|Mobic\|Mobic\|Mobic\|Mobic Palexa\|Mobic Palexia Palexia\|Mobic Methotrexate Salazopyrin\|Mobic\|Mobic | 25 |
| Morphine | (?i)oxycont\|physep\|methadone\|Methodone\|physopt\|oxy contin\|morphin | methadone 30mg bd\|methadone\|Tofacitinib Methodone\|Tofacitinib Methodone\|lyrica methadone\|PHYSOPTONE\|PHYSEPTONE\|PHYSEPTONE\|PHYSEPTONE\|Methadone\|Methadone\|Methadone\|METHADONE\|COSENTYX METHODONE PALEXIA\|endone & oxycontin\|Oxycontin\|morphine patch\|morphine\|Physeptone\|Physeptone\|Physeptone\|Physeptone\|Physeptone\|Physeptone\|Physeptone\|Physeptone\|Secukinumab Physeptone\|Secukinumab Physeptone\|Guselkumab Physeptone\|Guselkumab Physeptone\|oxycontin\|Oxycontin CR-Tablet 10 mg\|oxycontin oxynorm\|Oxycontin pregabalin\|oxycontin\|olumiant OxyContin 10 mg twice daily\|oxy contin\|MORPHIN PATCH\|oxycontin/ targin lyrica\|prednisone,oxycontin,lyrica,etc\|oxycontin prednisone oxycontin lyrica\|oxycontin-nortriptyline-lyrica-prednisone-enbrel injections-methotrexate-folic acid- prednisone enbrel\|oxycontin-nortriptyline-lyrica-prednisone-enbrel injections-methotrexate-folic acid-\|oxycontin-nortriptyline-lyrica-prednisone-enbrel injections-methotrexate-folic acid-\|oxycontin-nortriptyline-lyrica-prednisone-enbrel injections-methotrexate-folic acid-\|Methadone\|Physeptone\|Cosentyx Methadone | 45 |
| Naproxen | (?i)napr\|proxen | Naproxen\|Naproxen\|Naproxen\|Naproxen\|Naproxen\|NAPROXEN 750 MG\|Naproxen\|Naproxen\|Naproxen\|ProxenSR\|ProxenSR\|ProxenSR\|ProxenSR\|Naproxen occasionally Osteo Panadol occasionally\|naproxen when needed\|Naprosyn 20mg\|Naproxen\|Naproxen\|Naprosyn 550/\|Naprosyn 550/\|Naprosyn 550/\|Naprosyn 550/ Metoprolol Sandoz Cavstat\|Naprosyn 550/ Panadol ostia\|Naprosyn 550/\|Naprosyn 550/ | 7 |
| Paracetamol | (?i)paracet\|panado\|panod\|panamax\|acetamin\|tylen\|parasol osteo\|Panasonic Osteo\|Panedol Osteo\|panaol osteo\|Panafon osteo\|Pan idol osteo\|ostiomol\|osteomol\|ostemol | Panafon osteo\|Panadol osteo\|endep tab 10mg endep osto paracetamol\|ostio panadol\|panadol osteo\|Panadol Osteo\|Panadol Osteo\|panadol osteo doloxene\|Methotrexate Plaquenil Panadol Oster\|Panadol Osteo\|Panadol Osteo\|Panadol Osteo\|Panadol\|Panadol\|panadol osteo as needed\|Panadol osteo\|Panasonic osteo\|Panadol osteo\|Panadol osteo\|Panadol osteo Targin\|Panadol Osteo\|rinvoq panadol osteo\|Nexium, Lyrica Panadol osteo\|panadol osteo\|panadol osteo\|panadol osteo\|Panadol Osteo\|Panadol Osteo\|Panadol Osteo\|panadol osteo\|Panadol osteo, Panadine forte\|panadol osteo\|panadol osteo\|Panadol Osteo 665mg\|panadol osteo\|panadol osteo\|panadol osteo\|panadol osteo\|panadol osteo\|Panadol Osteo\|Panadol osteo\|rivoq Panadol osteo Panadol osteo\|rinvoq Panadol osteo\|rinvoq Panadol osteo\|Osteomol 665\|Panadol Osteo\|Panadol Osteo\|panadol osteo\|Panadol Osteo\|Osteo Panadol 2 Three times a day\|Osteo Panadol\|Panadol Osteo\|Osteo Panadol Osteo Panadol, Lyrica and Endep\|GOLD TABLETS panadol osteo\|panamax PRN\|panadol\|Panadol Osteo\|Panadol OSTEO\|oesto panadol\|OSTEO PANADOL\|Pan idol osteo\|panadol osteo panadol osteo\|panadol osteo\|panadol\|OESTO PANADOL\|OSTEO PANADOL\|OESTO PANADOL NORSPAN PATCH\|targan 5mg slow release prn osteo panadol tabs 2 tds\|Panadol Osteo PRN\|Panadol Osteo\|Panadol Osteo\|Panadol Osteo\|Panadol Osteo\|Panadol Osteo\|Endep 10 - Amitriptyline - for Nerve Pain in feet Panadol Osteo\|Endep 10 - Amitriptyline - for Nerve Pain in feet Panadol Oesto\|Endep 10 - Amitriptyline - for Nerve Pain in feet Panadol Osteo\|Prednisolone Tumeric Powder Panadol-Osteo\|Panadol Osteo\|Panadol Osteo\|Panadol Osteo\|osteo panadol\|Panadol forte\|Panadol Osteo\|Panadol osteo\|panadol osteo\|panadol osteo\|panadol osteo\|panadol osteo\|Panadol Osteo\|Panadol Osteo\|Panadol Osteo\|Panadol Osteo Targin 2.5mg\|Panadol osteo\|Panado/Osteo\|Panadol Osteo\|Panadol osteoporosis\|panadoloesteo panadoloesteo\|Panadoll Osteo\|Tylenol\|Panadol osteo\|Panadol Osteo\|Panadol Osteo\|consentyx panadol osteo\|Panadol Osteo\|Panadol Osteo\|Panadol Osteo\|Panadol Osteo\|Panadol Osteo Panadol Osteo\|panamax\|Olumiant - Baricitinib Prednislone osteo panadol\|Panadol\|Panadol\|Panadol Panadeine Forte\|Panasonic Osteo\|OSTIOMOL\|Panadol\|Osteo panadol\|Panadol Osteo\|osteo panadol\|Osteo Panadol\|Osteo Panadol\|Osteo Panadol\|Panadol\|osteo panadol none osteo panadol\|panadol osteo\|panadol ostio\|panadol ostio\|panadol ostio panadol ostio\|panadok ostio\|mersyndol panadol ostio\|panadol osteo\|Panadol osteo\|osteo panadol\|Oesteopanadol\|Oestopanadol\|HERON PARACETAMOL\|panadol osteo\|Panadol Osteo\|Panadol osteo\|Panadol Osteo\|Panadol Osteo Panadol osteo\|Ibuprofen is for hernia operation Endone & Paracetemol are for hernia operation\|panadol osteo\|Atorvastatin Paracetamol\|Panadol Osteo\|panadol osteo\|panadol osteo\|Panadol Oesteo\|Panadol Oesteo\|Panadol Osteo\|OSTEOMOL\|Panadol Osteo\|Panadol Osteo\|Panadol Osteo\|panadol osteo\|panadol osteo\|salazopyrin panadol osteo\|Panadol Osteo\|Panadol Osteo\|Panadol Oseto\|Cortisone Panadol osteo\|Osteo Panadol\|Lyrica Osteopanadol\|panadol osteo\|Panadol Osteo\|Osteo Panadol\|Panadol Osteo\|Panadol Osteo\|Osteo Panadol\|Panadol Osteo 650mg\|osteo panadol\|olumiant Osteo Panadol\|olumiant Panadol Osteo\|Panadol osteo Panadine Forte\|Panadol Osteo\|panadol osteo\|Fungilin Panadol Osteo\|Panadol Osteo\|Panadol Osteo\|APREMALIST PANADOL.OSTEO\|panamax\|Occasional Panadol Osteo\|Panadol osteo\|Panadol Osteo\|Nurofen plus Paracetamol\|panadol osteo\|Panadol Osteo\|Panadol Osteo\|Panadol Osteo\|Panadol Osteo\|Panadol Osteo\|Panadol Osteo\|paracetamol voltran 25 mg (occasinally)\|Paracetamol\|paracetamol\|paracetamol\|Panadol Oesto\|Panadol Oesto\|panadol osteo\|Panadol Osteo\|Panadol Osteo\|Panamax\|Panadol Osteo\|Panadol Osteo\|panadol osteo\|Panadol Osteo\|Panadol Osteo\|panadol osteo\|Panadol Osteo Panadol Osteo\|PANADOL OSTEO\|Panadol\|panadol osteo\|panadol\|panadol osteo\|panamax\|panadol osteo\|panadol osteo\|panadol osteo\|Panadol Osteo Targin\|Panadol osteo Pregabalin Targin - on occasions Plaquenil\|Panadol Oesteo\|Zaldair - Tramadol/Paracetamol\|Zaldair - Tramadol/Paracetamol\|Zaldiar- Tramadol/Paracetamol\|OSTEOMOL\|Panadol Osteo Panadol\|panadol osteo\|panadol ostio\|panadol osteo\|Panadol Osteo\|Paracetamol\|osteo paracetamol\|PANADOL OESTEO\|Cosentyx panadol oesteo\|Cosentyx Panadol oesteo\|panadol osteo\|panadol osteo\|Panadol osteo\|Panadol Osteo\|Panadol Osteo\|Panadol Osteo\|Panadol Osteo\|Osteo Panadol\|Panadol Osteo\|panadol osteo\|panadol osteo\|panaol osteo\|panaol osteo\|panaol osteo\|Panadol Oesto\|Targin Panadol oesto\|Panadol Oesto Targin\|Panadol Osteo\|Panadol Osteo\|Osteo Panadol\|Panadol Osteo\|Panadol Osteo\|paracetamol\|paracetamol osto\|Panadol osteo\|Panadol Osteo\|panadol osteo\|Prednisolone Osteo-Paracetamol\|Voltarin 12 hourly Gel Panadol Osteo\|Cartia Panadol\|Paracetemol Methotrexate\|upadacitinib Panadol Osteo\|Panadol Oste\|Pariet (rabeprazole sodium) panadol osteo\|panadol\|PANADOL\|Panadol Osteo\|Panadol\|Austopanadol\|osteo panadol\|osteo panadol\|osteo panadol\|Olumiant 4 g Osteo Panadol\|panadol ostio panadol forte\|panadol ostio\|panadol ostio\|Paracetamol\|Panadol osteo\|Panadol Osteo\|panadol osteo\|panadol osteo\|Panadol osteo\|Panadol Osteo Durogesic 25 Patch\|Panadol Osteo\|Panadol Osteo\|Occasionally Ostio Panadol\|osteo Panadol Osteo Panadol\|Osteo Panadol\|Naproxen occasionally Osteo Panadol occasionally\|panadol osteo\|panadol osteo\|panadol osteo\|panadol osteo\|panadol osteo\|Panadol Osteo\|Targin panadol osteo\|Panadol\|Panadol Osteo\|Panadol Osteo\|Panadol Osteo\|panadol Oesty Panadol Oesty\|acetaminophen\|panadol ostoe\|panadol osteo\|Panedol Osteo\|Oesto panadol\|Panadol osteo\|panadol osteo panadol osteo allosig\|Allosig PanadolOsteo\|Panadol-Osteo\|Panadol Osteo\|Panadol Osteo\|panadoloesto\|Panadol-osteo\|Panadol Osteo\|Panadol Oseto\|Panadol Osteo\|rinvoq Osteomol 665\|Panadol\|Panadol Osteo\|Panadol Osteo\|panadol osteo\|Prednisone Methotrexate Panadol Osteo\|Panadol Osteo\|Prednisone Methotrexate Targin Panadol osteo\|Allopurinol 500 mg Panadol Osteo Targin\|Panadol Osteo Targin\|panadol osteo\|Panasonic osteo\|PANADOL OSTEO\|panadol osteo\|panadol osteo\|panadol osteo\|Brufen - only as required for inflammation management Panadol - only as required for pain management Codiene - only as required for pain management\|Panadol - only as required for pain management\|Panadol Osteo\|Panadol Osteo\|panadol osteo\|panadol osteo\|panadol osteo\|panadol osteo\|Nil Panadol osteo\|Panadol Osteo\|Panadol Forte\|panamax neurofen plus panamax neurofen plus\|panamax\|Panadol Osteo Gabapentin\|panadol osteo\|Panadol Osteo Tramadol SR\|Panadol osteo\|Palexia Osteo Panadol\|Panadol Osteo prn\|Panadol Osteo\|Panadol Osteo\|panadol osteo\|panadol osteo\|Panadolosteo Norspan patch 5mcg/hr\|Parasol osteo\|Panadol osteo Panadeine forte\|Actemra Panadol osteo Paradise forte\|panodol osteo\|panadol osteo\|Panadol Osteo Palexia\|panadol\|Panadol osteo\|panadol osteo\|panadol osteo\|panadol osteo\|panadol osteo\|panadol osteo\|Panadol osteo Pregabalin\|Panadol Osteo\|Panadol Osteo\|Panadol Osteo\|PANADOL OSTEO\|Panadol Osteo Lyrica\|Panadol Osteo\|Panadol Osteo & Palexia IR\|Curcumin Panadol Osteo\|Paracetamol\|strong pain relief, panadol\|Panadol\|Panasonic osteo\|panadol osteo\|panadol osteo\|panadol osteo - which is rarely needed now\|cosenyx prednisolone - as needed panadol osteo\|Panadol\|Panadol Osteo\|Panadol Osteoporosis\|paracetamol voltaren gel\|panadol osteo mobic- rarely\|PANADOL OSTEO\|Have not taken for months panadol osteo\|PANADOL OSTEO\|Panadol Osteo\|Panadol Osteo\|Panadol Forte\|Paracetamol\|Panadol Osteo when needed\|Panadol Osteo\|Panadol Osteo\|Panadol Osteo\|Panadol Osteo\|Panadol Osteo\|Panadol osteo\|panadol osteo\|Panadol Osteo\|Panadol osteo\|Panadol Osteo Cymbalta\|Panadol osteo Zolpidem Tartrate\|Panadol Osteo\|cosentyx Panadol osteo\|cosentyx Osteo panadol\|Panadol Curcumin\|Panadol Curcumin curcumin\|Prednisone for 4 months Panadol Osteo\|Panadol Osteo\|Panadol\|zaldiar (paracetamol and tramadol combined)\|zaldiar (paracetamol and tramadol combined)\|zaldiar (paracetamol and tramadol combined)\|Zaldiar (Tramadol and paracetamol)\|Zaldiar (Tramadol and paracetamol)\|Clonazepam Zaldiar (Tramadol and paracetamol)\|Clonazepam Zaldiar (Tramadol/Paracetamol combination)\|Clonazepam Zaldiar (Tramadol/Paracetamol combination)\|inflectra PARACETAMOL 665mg\|panadol\|cosentyx Panadol Osteo\|panadol osteo panadol osteo\|panadol osteo panadol osteo\|Panadol osteo\|osteomol paracetamol\|panadol osteo\|Panadolosteo\|Panadol, Trymol\|Panadol Tramdol\|Panadol Osteo\|Panadol Osteo\|Prednisolone Panadol osteo\|Duloxetine telmisarten osteo Panadol\|Osteo Panadol\|Panadol Osteo\|Panadol Osteo\|Panadol Osteo\|OSTEO PANADOL\|Panadol osteo Panadol osteo\|Baricitinib Osteomol\|PANAMAX\|Panodol Osteo\|Panadol Osteo\|Panadol Oesteo\|Cosentyx )secukinumab Panadol Oesteo\|Cosentyx )secukinumab Panadol Oesteo\|Naprosyn 550/ Panadol ostia\|panadol ostio\|Traumel Curcurmin Osteo Panadol\|parasol osteo\|SECUKINUMAB PANADOL OESTO\|SECUKINUMAB Panadol Oesto\|Panadol Osteo\|panadol osteo\|Hequinal osteopanadol\|Hequinal Panadol osteo\|panadol osteo\|Celecoxib Panadol Osteo\|panadol osteo\|panadol osteo\|panadol osteo\|Panadol osteo\|Panadol Osteo\|Ostemol Magnesium Forte\|Paracetamol\|Osteo panadol\|Panadol osteo\|Panadol osteo\|Panadol osteo\|Panadol osteo\|Panadol Osteomol Panadol Osteo\|665 paracetamol\|Panadol osteo\|Methotrexate tablets Prednisone Panadol Osteo\|Panadol osteo\|Osteo Paracetamol 665mg\|Advil panadol\|Paracetamol (allergic to NSAIDS) Palexia SR 50mg Epilim 300mg BD for nerve pain\|Paracetamol (allergic to NSAIDS) Epilim for nerve pain\|Osteo panadol 665\|Panadol Osteo\|Panasonic osteo\|Panasonic osteo\|Panadol Osteo\|Panadol Osteo\|Targen Panadol osteo\|Taltz (Ixekizumab) Panadol osteo\|Taltz (Ixekizumab) Targin 7.5/15 Panadol osteo\|Taltz (Ixekizumab) Targin Panadol osteo\|Osteo Panadol\|Osteo Panadol Osteo Panadol\|panadol ostio | 365 |
| Paracetamol & codeine | (?i)panade\|panadi\|codapan\|prodeine\|Panadol Forte\|Codalgin\|Panedeine | panadeine\|Panadol osteo, Panadine forte\|panadeine forte Norspan patch\|Panadeine Forte\|Panedeine\|Panadiene Forte\|Panadol forte\|PANADINE OSTEO\|Panadol Panadeine Forte\|Panadeine Forte\|Panadine Forte\|Panadine Forte\|Panadol osteo Panadine Forte\|panadeine forte\|Panadeine Forte\|PRODEINE FORTE\|Panadene Osteo\|ZEN CODAPANE FORTE\|panadein forte\|Panadine Forte\|panadeine forte\|panadeane forte\|panadol ostio panadol forte\|Panadean fort\|Codalgin Forte\|panadeine forte\|olumiant panadeine forte\|olumiant Panadeine forte\|Panadol Forte\|Panadol osteo Panadeine forte\|Panadene Forte\|panadeine forte when required allegron when required\|Panadol Forte\|PREDNISOLONE--PRN PANADEINE FORTE\|herbal panadene forte\|panadeine forte\|Panadin Forte\|Sulfasalazine embrel Panadeine forte\|prednisolone Panadeine forte | 29 |
| Tramadol | (?i)tram\|traum | Tramadol\|Tramadol\|Tramadol 50mg\|TRAMAL LYRICA\|DUROTRAM 300 MGS\|Tramal\|Tramal S R\|Tramal SR\|S R Tramal\|Tramal\|SR Tramal\|SR Tramal\|SR Tramal\|Zaldair - Tramadol/Paracetamol\|Zaldair - Tramadol/Paracetamol\|Zaldiar- Tramadol/Paracetamol\|Tramal\|Tramadol as needed\|DUROTRAM\|DUROTRAM\|DUROTRAM\|Panadol Osteo Tramadol SR\|Tramadol\|zaldiar (paracetamol and tramadol combined)\|zaldiar (paracetamol and tramadol combined)\|zaldiar (paracetamol and tramadol combined)\|Zaldiar (Tramadol and paracetamol)\|Zaldiar (Tramadol and paracetamol)\|Clonazepam Zaldiar (Tramadol and paracetamol)\|Clonazepam Zaldiar (Tramadol/Paracetamol combination)\|Clonazepam Zaldiar (Tramadol/Paracetamol combination)\|Panadol Tramdol\|Traumel Curcurmin Osteo Panadol\|Traumel\|Traumeel | 26 |

# Supplementary Table S3. Difference in proportions of false self-reports by various characteristics

| **Medication name** | **Group** | **Strata** | **TP** | **FP** | **FN** | **TN** | **NA** | **Total True Self-reports^*^** | **Total False Self-reports^†^** | **Proportion False Self-reports (%)** |
| --- | --- | --- | --- | --- | --- | --- | --- | --- | --- | --- |
| Abatacept - infusion | Current smoker | yes | 28 | 6 | 3 | 1,663 | 1 | 1,691 | 9 | 0.53 |
|  |  | no | 337 | 85 | 30 | 21,497 | 28 | 21,834 | 115 | 0.53 |
|  | Current depression or anxiety | yes | 140 | 46 | 22 | 8,994 | 28 | 9,134 | 68 | 0.74 |
|  |  | no | 225 | 45 | 11 | 14,166 | 1 | 14,391 | 56 | 0.39 |
|  | Questionnaire type | online | 211 | 66 | 27 | 16,054 | 21 | 16,265 | 93 | 0.57 |
|  |  | paper | 154 | 25 | 6 | 7,106 | 8 | 7,260 | 31 | 0.43 |
|  | Gender | male | 84 | 16 | 4 | 7,762 | 0 | 7,846 | 20 | 0.25 |
|  |  | female | 281 | 75 | 29 | 15,398 | 29 | 15,679 | 104 | 0.66 |
|  | Currently married | yes | 263 | 67 | 24 | 16,129 | 14 | 16,392 | 91 | 0.56 |
|  |  | no | 102 | 24 | 9 | 7,031 | 15 | 7,133 | 33 | 0.46 |
|  | Current pain | yes | 313 | 80 | 31 | 19,704 | 29 | 20,017 | 111 | 0.55 |
|  |  | no | 52 | 11 | 2 | 3,456 | 0 | 3,508 | 13 | 0.37 |
|  | Tertiary qualification | yes | 148 | 42 | 13 | 12,108 | 20 | 12,256 | 55 | 0.45 |
|  |  | no | 217 | 49 | 20 | 11,052 | 9 | 11,269 | 69 | 0.61 |
| Abatacept - injection | Current smoker | yes | 69 | 2 | 12 | 1,618 | 0 | 1,687 | 14 | 0.83 |
|  |  | no | 634 | 77 | 142 | 21,122 | 2 | 21,756 | 219 | 1.01 |
|  | Current depression or anxiety | yes | 309 | 32 | 71 | 8,816 | 2 | 9,125 | 103 | 1.13 |
|  |  | no | 394 | 47 | 83 | 13,924 | 0 | 14,318 | 130 | 0.91 |
|  | Questionnaire type | online | 418 | 56 | 113 | 15,791 | 1 | 16,209 | 169 | 1.04 |
|  |  | paper | 285 | 23 | 41 | 6,949 | 1 | 7,234 | 64 | 0.88 |
|  | Gender | male | 167 | 26 | 29 | 7,644 | 0 | 7,811 | 55 | 0.70 |
|  |  | female | 536 | 53 | 125 | 15,096 | 2 | 15,632 | 178 | 1.14 |
|  | Currently married | yes | 480 | 51 | 99 | 15,867 | 0 | 16,347 | 150 | 0.92 |
|  |  | no | 223 | 28 | 55 | 6,873 | 2 | 7,096 | 83 | 1.17 |
|  | Current pain | yes | 655 | 74 | 142 | 19,284 | 2 | 19,939 | 216 | 1.08 |
|  |  | no | 48 | 5 | 12 | 3,456 | 0 | 3,504 | 17 | 0.49 |
|  | Tertiary qualification | yes | 308 | 44 | 69 | 11,910 | 0 | 12,218 | 113 | 0.92 |
|  |  | no | 395 | 35 | 85 | 10,830 | 2 | 11,225 | 120 | 1.07 |
| Adalimumab | Current smoker | yes | 373 | 14 | 57 | 1,250 | 7 | 1,623 | 71 | 4.37 |
|  |  | no | 4,694 | 253 | 353 | 16,611 | 66 | 21,305 | 606 | 2.84 |
|  | Current depression or anxiety | yes | 1,809 | 114 | 153 | 7,130 | 24 | 8,939 | 267 | 2.99 |
|  |  | no | 3,258 | 153 | 257 | 10,731 | 49 | 13,989 | 410 | 2.93 |
|  | Questionnaire type | online | 3,408 | 209 | 364 | 12,366 | 32 | 15,774 | 573 | 3.63 |
|  |  | paper | 1,659 | 58 | 46 | 5,495 | 41 | 7,154 | 104 | 1.45 |
|  | Gender | male | 2,081 | 131 | 139 | 5,486 | 29 | 7,567 | 270 | 3.57 |
|  |  | female | 2,986 | 136 | 271 | 12,375 | 44 | 15,361 | 407 | 2.65 |
|  | Currently married | yes | 3,545 | 180 | 306 | 12,428 | 38 | 15,973 | 486 | 3.04 |
|  |  | no | 1,522 | 87 | 104 | 5,433 | 35 | 6,955 | 191 | 2.75 |
|  | Current pain | yes | 4,093 | 223 | 352 | 15,419 | 70 | 19,512 | 575 | 2.95 |
|  |  | no | 974 | 44 | 58 | 2,442 | 3 | 3,416 | 102 | 2.99 |
|  | Tertiary qualification | yes | 2,702 | 150 | 244 | 9,214 | 21 | 11,916 | 394 | 3.31 |
|  |  | no | 2,365 | 117 | 166 | 8,647 | 52 | 11,012 | 283 | 2.57 |
| Azathioprine | Current smoker | yes | 0 | 0 | 1 | 1,671 | 29 | 1,671 | 1 | 0.06 |
|  |  | no | 129 | 43 | 17 | 21,574 | 214 | 21,703 | 60 | 0.28 |
|  | Current depression or anxiety | yes | 50 | 27 | 13 | 9,023 | 117 | 9,073 | 40 | 0.44 |
|  |  | no | 79 | 16 | 5 | 14,222 | 126 | 14,301 | 21 | 0.15 |
|  | Questionnaire type | online | 103 | 23 | 16 | 16,086 | 151 | 16,189 | 39 | 0.24 |
|  |  | paper | 26 | 20 | 2 | 7,159 | 92 | 7,185 | 22 | 0.31 |
|  | Gender | male | 20 | 14 | 5 | 7,710 | 117 | 7,730 | 19 | 0.25 |
|  |  | female | 109 | 29 | 13 | 15,535 | 126 | 15,644 | 42 | 0.27 |
|  | Currently married | yes | 87 | 31 | 12 | 16,234 | 133 | 16,321 | 43 | 0.26 |
|  |  | no | 42 | 12 | 6 | 7,011 | 110 | 7,053 | 18 | 0.26 |
|  | Current pain | yes | 113 | 38 | 17 | 19,775 | 214 | 19,888 | 55 | 0.28 |
|  |  | no | 16 | 5 | 1 | 3,470 | 29 | 3,486 | 6 | 0.17 |
|  | Tertiary qualification | yes | 88 | 20 | 13 | 12,094 | 116 | 12,182 | 33 | 0.27 |
|  |  | no | 41 | 23 | 5 | 11,151 | 127 | 11,192 | 28 | 0.25 |
| Celecoxib | Current smoker | yes | 134 | 56 | 20 | 1,491 | 0 | 1,625 | 76 | 4.68 |
|  |  | no | 1,399 | 589 | 264 | 19,725 | 0 | 21,124 | 853 | 4.04 |
|  | Current depression or anxiety | yes | 655 | 285 | 121 | 8,169 | 0 | 8,824 | 406 | 4.60 |
|  |  | no | 878 | 360 | 163 | 13,047 | 0 | 13,925 | 523 | 3.76 |
|  | Questionnaire type | online | 1,120 | 469 | 189 | 14,601 | 0 | 15,721 | 658 | 4.19 |
|  |  | paper | 413 | 176 | 95 | 6,615 | 0 | 7,028 | 271 | 3.86 |
|  | Gender | male | 436 | 188 | 94 | 7,148 | 0 | 7,584 | 282 | 3.72 |
|  |  | female | 1,097 | 457 | 190 | 14,068 | 0 | 15,165 | 647 | 4.27 |
|  | Currently married | yes | 1,098 | 461 | 201 | 14,737 | 0 | 15,835 | 662 | 4.18 |
|  |  | no | 435 | 184 | 83 | 6,479 | 0 | 6,914 | 267 | 3.86 |
|  | Current pain | yes | 1,449 | 593 | 252 | 17,863 | 0 | 19,312 | 845 | 4.38 |
|  |  | no | 84 | 52 | 32 | 3,353 | 0 | 3,437 | 84 | 2.44 |
|  | Tertiary qualification | yes | 834 | 395 | 124 | 10,978 | 0 | 11,812 | 519 | 4.39 |
|  |  | no | 699 | 250 | 160 | 10,238 | 0 | 10,937 | 410 | 3.75 |
| Certolizumab pegol | Current smoker | yes | 22 | 2 | 10 | 1,667 | 0 | 1,689 | 12 | 0.71 |
|  |  | no | 517 | 43 | 68 | 21,342 | 7 | 21,859 | 111 | 0.51 |
|  | Current depression or anxiety | yes | 214 | 15 | 36 | 8,962 | 3 | 9,176 | 51 | 0.56 |
|  |  | no | 325 | 30 | 42 | 14,047 | 4 | 14,372 | 72 | 0.50 |
|  | Questionnaire type | online | 358 | 41 | 67 | 15,907 | 6 | 16,265 | 108 | 0.66 |
|  |  | paper | 181 | 4 | 11 | 7,102 | 1 | 7,283 | 15 | 0.21 |
|  | Gender | male | 172 | 13 | 18 | 7,663 | 0 | 7,835 | 31 | 0.40 |
|  |  | female | 367 | 32 | 60 | 15,346 | 7 | 15,713 | 92 | 0.59 |
|  | Currently married | yes | 432 | 36 | 53 | 15,971 | 5 | 16,403 | 89 | 0.54 |
|  |  | no | 107 | 9 | 25 | 7,038 | 2 | 7,145 | 34 | 0.48 |
|  | Current pain | yes | 484 | 39 | 77 | 19,550 | 7 | 20,034 | 116 | 0.58 |
|  |  | no | 55 | 6 | 1 | 3,459 | 0 | 3,514 | 7 | 0.20 |
|  | Tertiary qualification | yes | 283 | 27 | 43 | 11,973 | 5 | 12,256 | 70 | 0.57 |
|  |  | no | 256 | 18 | 35 | 11,036 | 2 | 11,292 | 53 | 0.47 |
| Cyclosporin | Current smoker | yes | 7 | 0 | 0 | 1,680 | 14 | 1,687 | 0 | 0.00 |
|  |  | no | 66 | 15 | 13 | 21,656 | 227 | 21,722 | 28 | 0.13 |
|  | Current depression or anxiety | yes | 36 | 6 | 6 | 9,051 | 131 | 9,087 | 12 | 0.13 |
|  |  | no | 37 | 9 | 7 | 14,285 | 110 | 14,322 | 16 | 0.11 |
|  | Questionnaire type | online | 24 | 0 | 9 | 16,174 | 172 | 16,198 | 9 | 0.06 |
|  |  | paper | 49 | 15 | 4 | 7,162 | 69 | 7,211 | 19 | 0.26 |
|  | Gender | male | 8 | 1 | 4 | 7,733 | 120 | 7,741 | 5 | 0.06 |
|  |  | female | 65 | 14 | 9 | 15,603 | 121 | 15,668 | 23 | 0.15 |
|  | Currently married | yes | 70 | 11 | 8 | 16,246 | 162 | 16,316 | 19 | 0.12 |
|  |  | no | 3 | 4 | 5 | 7,090 | 79 | 7,093 | 9 | 0.13 |
|  | Current pain | yes | 72 | 15 | 12 | 19,835 | 223 | 19,907 | 27 | 0.14 |
|  |  | no | 1 | 0 | 1 | 3,501 | 18 | 3,502 | 1 | 0.03 |
|  | Tertiary qualification | yes | 10 | 0 | 6 | 12,184 | 131 | 12,194 | 6 | 0.05 |
|  |  | no | 63 | 15 | 7 | 11,152 | 110 | 11,215 | 22 | 0.20 |
| Etanercept | Current smoker | yes | 358 | 24 | 22 | 1,296 | 1 | 1,654 | 46 | 2.78 |
|  |  | no | 4,450 | 194 | 327 | 16,973 | 33 | 21,423 | 521 | 2.43 |
|  | Current depression or anxiety | yes | 1,786 | 84 | 111 | 7,228 | 21 | 9,014 | 195 | 2.16 |
|  |  | no | 3,022 | 134 | 238 | 11,041 | 13 | 14,063 | 372 | 2.65 |
|  | Questionnaire type | online | 3,222 | 171 | 288 | 12,671 | 27 | 15,893 | 459 | 2.89 |
|  |  | paper | 1,586 | 47 | 61 | 5,598 | 7 | 7,184 | 108 | 1.50 |
|  | Gender | male | 1,644 | 69 | 96 | 6,048 | 9 | 7,692 | 165 | 2.15 |
|  |  | female | 3,164 | 149 | 253 | 12,221 | 25 | 15,385 | 402 | 2.61 |
|  | Currently married | yes | 3,375 | 140 | 262 | 12,692 | 28 | 16,067 | 402 | 2.50 |
|  |  | no | 1,433 | 78 | 87 | 5,577 | 6 | 7,010 | 165 | 2.35 |
|  | Current pain | yes | 3,977 | 186 | 288 | 15,673 | 33 | 19,650 | 474 | 2.41 |
|  |  | no | 831 | 32 | 61 | 2,596 | 1 | 3,427 | 93 | 2.71 |
|  | Tertiary qualification | yes | 2,421 | 125 | 194 | 9,575 | 16 | 11,996 | 319 | 2.66 |
|  |  | no | 2,387 | 93 | 155 | 8,694 | 18 | 11,081 | 248 | 2.24 |
| Gold - intramuscular | Current smoker | yes | 0 | 0 | 1 | 1,682 | 18 | 1,682 | 1 | 0.06 |
|  |  | no | 25 | 14 | 10 | 21,806 | 122 | 21,831 | 24 | 0.11 |
|  | Current depression or anxiety | yes | 9 | 8 | 4 | 9,141 | 68 | 9,150 | 12 | 0.13 |
|  |  | no | 16 | 6 | 7 | 14,347 | 72 | 14,363 | 13 | 0.09 |
|  | Questionnaire type | online | 13 | 11 | 6 | 16,285 | 64 | 16,298 | 17 | 0.10 |
|  |  | paper | 12 | 3 | 5 | 7,203 | 76 | 7,215 | 8 | 0.11 |
|  | Gender | male | 5 | 0 | 2 | 7,798 | 61 | 7,803 | 2 | 0.03 |
|  |  | female | 20 | 14 | 9 | 15,690 | 79 | 15,710 | 23 | 0.15 |
|  | Currently married | yes | 20 | 13 | 7 | 16,372 | 85 | 16,392 | 20 | 0.12 |
|  |  | no | 5 | 1 | 4 | 7,116 | 55 | 7,121 | 5 | 0.07 |
|  | Current pain | yes | 24 | 14 | 11 | 19,986 | 122 | 20,010 | 25 | 0.12 |
|  |  | no | 1 | 0 | 0 | 3,502 | 18 | 3,503 | 0 | 0.00 |
|  | Tertiary qualification | yes | 10 | 9 | 7 | 12,282 | 23 | 12,292 | 16 | 0.13 |
|  |  | no | 15 | 5 | 4 | 11,206 | 117 | 11,221 | 9 | 0.08 |
| Golimumab | Current smoker | yes | 100 | 6 | 12 | 1,583 | 0 | 1,683 | 18 | 1.07 |
|  |  | no | 1,297 | 99 | 133 | 20,444 | 4 | 21,741 | 232 | 1.07 |
|  | Current depression or anxiety | yes | 492 | 43 | 54 | 8,638 | 3 | 9,130 | 97 | 1.06 |
|  |  | no | 905 | 62 | 91 | 13,389 | 1 | 14,294 | 153 | 1.07 |
|  | Questionnaire type | online | 983 | 96 | 118 | 15,179 | 3 | 16,162 | 214 | 1.32 |
|  |  | paper | 414 | 9 | 27 | 6,848 | 1 | 7,262 | 36 | 0.50 |
|  | Gender | male | 561 | 53 | 41 | 7,211 | 0 | 7,772 | 94 | 1.21 |
|  |  | female | 836 | 52 | 104 | 14,816 | 4 | 15,652 | 156 | 1.00 |
|  | Currently married | yes | 1,017 | 79 | 99 | 15,302 | 0 | 16,319 | 178 | 1.09 |
|  |  | no | 380 | 26 | 46 | 6,725 | 4 | 7,105 | 72 | 1.01 |
|  | Current pain | yes | 1,137 | 83 | 127 | 18,806 | 4 | 19,943 | 210 | 1.05 |
|  |  | no | 260 | 22 | 18 | 3,221 | 0 | 3,481 | 40 | 1.15 |
|  | Tertiary qualification | yes | 740 | 58 | 74 | 11,457 | 2 | 12,197 | 132 | 1.08 |
|  |  | no | 657 | 47 | 71 | 10,570 | 2 | 11,227 | 118 | 1.05 |
| Hydroxychloroquine | Current smoker | yes | 165 | 44 | 17 | 1,440 | 35 | 1,605 | 61 | 3.80 |
|  |  | no | 2,717 | 366 | 366 | 18,352 | 176 | 21,069 | 732 | 3.47 |
|  | Current depression or anxiety | yes | 1,076 | 198 | 177 | 7,689 | 90 | 8,765 | 375 | 4.28 |
|  |  | no | 1,806 | 212 | 206 | 12,103 | 121 | 13,909 | 418 | 3.01 |
|  | Questionnaire type | online | 1,958 | 311 | 283 | 13,674 | 153 | 15,632 | 594 | 3.80 |
|  |  | paper | 924 | 99 | 100 | 6,118 | 58 | 7,042 | 199 | 2.83 |
|  | Gender | male | 512 | 114 | 66 | 7,059 | 115 | 7,571 | 180 | 2.38 |
|  |  | female | 2,370 | 296 | 317 | 12,733 | 96 | 15,103 | 613 | 4.06 |
|  | Currently married | yes | 2,044 | 285 | 265 | 13,741 | 162 | 15,785 | 550 | 3.48 |
|  |  | no | 838 | 125 | 118 | 6,051 | 49 | 6,889 | 243 | 3.53 |
|  | Current pain | yes | 2,552 | 379 | 347 | 16,701 | 178 | 19,253 | 726 | 3.77 |
|  |  | no | 330 | 31 | 36 | 3,091 | 33 | 3,421 | 67 | 1.96 |
|  | Tertiary qualification | yes | 1,419 | 197 | 209 | 10,396 | 110 | 11,815 | 406 | 3.44 |
|  |  | no | 1,463 | 213 | 174 | 9,396 | 101 | 10,859 | 387 | 3.56 |
| Indometacin | Current smoker | yes | 7 | 8 | 6 | 1,680 | 0 | 1,687 | 14 | 0.83 |
|  |  | no | 177 | 74 | 92 | 21,634 | 0 | 21,811 | 166 | 0.76 |
|  | Current depression or anxiety | yes | 70 | 40 | 46 | 9,074 | 0 | 9,144 | 86 | 0.94 |
|  |  | no | 114 | 42 | 52 | 14,240 | 0 | 14,354 | 94 | 0.65 |
|  | Questionnaire type | online | 122 | 56 | 52 | 16,149 | 0 | 16,271 | 108 | 0.66 |
|  |  | paper | 62 | 26 | 46 | 7,165 | 0 | 7,227 | 72 | 1.00 |
|  | Gender | male | 61 | 25 | 42 | 7,738 | 0 | 7,799 | 67 | 0.86 |
|  |  | female | 123 | 57 | 56 | 15,576 | 0 | 15,699 | 113 | 0.72 |
|  | Currently married | yes | 91 | 60 | 67 | 16,279 | 0 | 16,370 | 127 | 0.78 |
|  |  | no | 93 | 22 | 31 | 7,035 | 0 | 7,128 | 53 | 0.74 |
|  | Current pain | yes | 168 | 79 | 88 | 19,822 | 0 | 19,990 | 167 | 0.84 |
|  |  | no | 16 | 3 | 10 | 3,492 | 0 | 3,508 | 13 | 0.37 |
|  | Tertiary qualification | yes | 87 | 35 | 56 | 12,153 | 0 | 12,240 | 91 | 0.74 |
|  |  | no | 97 | 47 | 42 | 11,161 | 0 | 11,258 | 89 | 0.79 |
| Infliximab | Current smoker | yes | 55 | 2 | 4 | 1,629 | 11 | 1,684 | 6 | 0.36 |
|  |  | no | 645 | 35 | 27 | 21,173 | 97 | 21,818 | 62 | 0.28 |
|  | Current depression or anxiety | yes | 299 | 9 | 16 | 8,859 | 47 | 9,158 | 25 | 0.27 |
|  |  | no | 401 | 28 | 15 | 13,943 | 61 | 14,344 | 43 | 0.30 |
|  | Questionnaire type | online | 494 | 25 | 26 | 15,765 | 69 | 16,259 | 51 | 0.31 |
|  |  | paper | 206 | 12 | 5 | 7,037 | 39 | 7,243 | 17 | 0.23 |
|  | Gender | male | 366 | 17 | 10 | 7,427 | 46 | 7,793 | 27 | 0.35 |
|  |  | female | 334 | 20 | 21 | 15,375 | 62 | 15,709 | 41 | 0.26 |
|  | Currently married | yes | 508 | 22 | 25 | 15,881 | 61 | 16,389 | 47 | 0.29 |
|  |  | no | 192 | 15 | 6 | 6,921 | 47 | 7,113 | 21 | 0.30 |
|  | Current pain | yes | 542 | 26 | 31 | 19,456 | 102 | 19,998 | 57 | 0.29 |
|  |  | no | 158 | 11 | 0 | 3,346 | 6 | 3,504 | 11 | 0.31 |
|  | Tertiary qualification | yes | 383 | 17 | 22 | 11,850 | 59 | 12,233 | 39 | 0.32 |
|  |  | no | 317 | 20 | 9 | 10,952 | 49 | 11,269 | 29 | 0.26 |
| Ketoprofen | Current smoker | yes | 23 | 2 | 1 | 1,675 | 0 | 1,698 | 3 | 0.18 |
|  |  | no | 183 | 71 | 28 | 21,695 | 0 | 21,878 | 99 | 0.45 |
|  | Current depression or anxiety | yes | 78 | 24 | 14 | 9,114 | 0 | 9,192 | 38 | 0.41 |
|  |  | no | 128 | 49 | 15 | 14,256 | 0 | 14,384 | 64 | 0.44 |
|  | Questionnaire type | online | 162 | 50 | 19 | 16,148 | 0 | 16,310 | 69 | 0.42 |
|  |  | paper | 44 | 23 | 10 | 7,222 | 0 | 7,266 | 33 | 0.45 |
|  | Gender | male | 49 | 16 | 7 | 7,794 | 0 | 7,843 | 23 | 0.29 |
|  |  | female | 157 | 57 | 22 | 15,576 | 0 | 15,733 | 79 | 0.50 |
|  | Currently married | yes | 142 | 53 | 21 | 16,281 | 0 | 16,423 | 74 | 0.45 |
|  |  | no | 64 | 20 | 8 | 7,089 | 0 | 7,153 | 28 | 0.39 |
|  | Current pain | yes | 198 | 71 | 25 | 19,863 | 0 | 20,061 | 96 | 0.48 |
|  |  | no | 8 | 2 | 4 | 3,507 | 0 | 3,515 | 6 | 0.17 |
|  | Tertiary qualification | yes | 110 | 44 | 13 | 12,164 | 0 | 12,274 | 57 | 0.46 |
|  |  | no | 96 | 29 | 16 | 11,206 | 0 | 11,302 | 45 | 0.40 |
| Leflunomide | Current smoker | yes | 188 | 67 | 23 | 1,423 | 0 | 1,611 | 90 | 5.59 |
|  |  | no | 2,119 | 333 | 284 | 19,032 | 209 | 21,151 | 617 | 2.92 |
|  | Current depression or anxiety | yes | 937 | 181 | 168 | 7,848 | 96 | 8,785 | 349 | 3.97 |
|  |  | no | 1,370 | 219 | 139 | 12,607 | 113 | 13,977 | 358 | 2.56 |
|  | Questionnaire type | online | 1,477 | 246 | 208 | 14,276 | 172 | 15,753 | 454 | 2.88 |
|  |  | paper | 830 | 154 | 99 | 6,179 | 37 | 7,009 | 253 | 3.61 |
|  | Gender | male | 558 | 146 | 83 | 6,971 | 108 | 7,529 | 229 | 3.04 |
|  |  | female | 1,749 | 254 | 224 | 13,484 | 101 | 15,233 | 478 | 3.14 |
|  | Currently married | yes | 1,518 | 223 | 210 | 14,382 | 164 | 15,900 | 433 | 2.72 |
|  |  | no | 789 | 177 | 97 | 6,073 | 45 | 6,862 | 274 | 3.99 |
|  | Current pain | yes | 2,090 | 365 | 268 | 17,272 | 162 | 19,362 | 633 | 3.27 |
|  |  | no | 217 | 35 | 39 | 3,183 | 47 | 3,400 | 74 | 2.18 |
|  | Tertiary qualification | yes | 1,133 | 185 | 150 | 10,742 | 121 | 11,875 | 335 | 2.82 |
|  |  | no | 1,174 | 215 | 157 | 9,713 | 88 | 10,887 | 372 | 3.42 |
| Meloxicam | Current smoker | yes | 74 | 60 | 18 | 1,549 | 0 | 1,623 | 78 | 4.81 |
|  |  | no | 1,375 | 658 | 323 | 19,621 | 0 | 20,996 | 981 | 4.67 |
|  | Current depression or anxiety | yes | 598 | 331 | 160 | 8,141 | 0 | 8,739 | 491 | 5.62 |
|  |  | no | 851 | 387 | 181 | 13,029 | 0 | 13,880 | 568 | 4.09 |
|  | Questionnaire type | online | 1,039 | 544 | 196 | 14,600 | 0 | 15,639 | 740 | 4.73 |
|  |  | paper | 410 | 174 | 145 | 6,570 | 0 | 6,980 | 319 | 4.57 |
|  | Gender | male | 345 | 188 | 91 | 7,242 | 0 | 7,587 | 279 | 3.68 |
|  |  | female | 1,104 | 530 | 250 | 13,928 | 0 | 15,032 | 780 | 5.19 |
|  | Currently married | yes | 1,048 | 496 | 234 | 14,719 | 0 | 15,767 | 730 | 4.63 |
|  |  | no | 401 | 222 | 107 | 6,451 | 0 | 6,852 | 329 | 4.80 |
|  | Current pain | yes | 1,363 | 654 | 291 | 17,849 | 0 | 19,212 | 945 | 4.92 |
|  |  | no | 86 | 64 | 50 | 3,321 | 0 | 3,407 | 114 | 3.35 |
|  | Tertiary qualification | yes | 826 | 381 | 158 | 10,966 | 0 | 11,792 | 539 | 4.57 |
|  |  | no | 623 | 337 | 183 | 10,204 | 0 | 10,827 | 520 | 4.80 |
| Methotrexate - injection | Current smoker | yes | 71 | 15 | 6 | 1,602 | 7 | 1,673 | 21 | 1.26 |
|  |  | no | 735 | 217 | 158 | 20,703 | 164 | 21,438 | 375 | 1.75 |
|  | Current depression or anxiety | yes | 352 | 125 | 87 | 8,598 | 68 | 8,950 | 212 | 2.37 |
|  |  | no | 454 | 107 | 77 | 13,707 | 103 | 14,161 | 184 | 1.30 |
|  | Questionnaire type | online | 561 | 181 | 132 | 15,401 | 104 | 15,962 | 313 | 1.96 |
|  |  | paper | 245 | 51 | 32 | 6,904 | 67 | 7,149 | 83 | 1.16 |
|  | Gender | male | 239 | 90 | 36 | 7,432 | 69 | 7,671 | 126 | 1.64 |
|  |  | female | 567 | 142 | 128 | 14,873 | 102 | 15,440 | 270 | 1.75 |
|  | Currently married | yes | 514 | 154 | 110 | 15,592 | 127 | 16,106 | 264 | 1.64 |
|  |  | no | 292 | 78 | 54 | 6,713 | 44 | 7,005 | 132 | 1.88 |
|  | Current pain | yes | 741 | 192 | 155 | 18,935 | 134 | 19,676 | 347 | 1.76 |
|  |  | no | 65 | 40 | 9 | 3,370 | 37 | 3,435 | 49 | 1.43 |
|  | Tertiary qualification | yes | 393 | 139 | 84 | 11,634 | 81 | 12,027 | 223 | 1.85 |
|  |  | no | 413 | 93 | 80 | 10,671 | 90 | 11,084 | 173 | 1.56 |
| Methotrexate - oral | Current smoker | yes | 790 | 123 | 76 | 706 | 6 | 1,496 | 199 | 13.30 |
|  |  | no | 9,868 | 1,197 | 1,077 | 9,740 | 95 | 19,608 | 2,274 | 11.60 |
|  | Current depression or anxiety | yes | 3,991 | 521 | 533 | 4,161 | 24 | 8,152 | 1,054 | 12.93 |
|  |  | no | 6,667 | 799 | 620 | 6,285 | 77 | 12,952 | 1,419 | 10.96 |
|  | Questionnaire type | online | 6,917 | 906 | 839 | 7,639 | 78 | 14,556 | 1,745 | 11.99 |
|  |  | paper | 3,741 | 414 | 314 | 2,807 | 23 | 6,548 | 728 | 11.12 |
|  | Gender | male | 2,954 | 379 | 309 | 4,175 | 49 | 7,129 | 688 | 9.65 |
|  |  | female | 7,704 | 941 | 844 | 6,271 | 52 | 13,975 | 1,785 | 12.77 |
|  | Currently married | yes | 7,489 | 878 | 750 | 7,321 | 59 | 14,810 | 1,628 | 10.99 |
|  |  | no | 3,169 | 442 | 403 | 3,125 | 42 | 6,294 | 845 | 13.43 |
|  | Current pain | yes | 9,290 | 1,128 | 1,040 | 8,614 | 85 | 17,904 | 2,168 | 12.11 |
|  |  | no | 1,368 | 192 | 113 | 1,832 | 16 | 3,200 | 305 | 9.53 |
|  | Tertiary qualification | yes | 5,060 | 637 | 635 | 5,951 | 48 | 11,011 | 1,272 | 11.55 |
|  |  | no | 5,598 | 683 | 518 | 4,495 | 53 | 10,093 | 1,201 | 11.90 |
| Morphine | Current smoker | yes | 12 | 15 | 7 | 1,667 | 0 | 1,679 | 22 | 1.31 |
|  |  | no | 91 | 156 | 41 | 21,689 | 0 | 21,780 | 197 | 0.90 |
|  | Current depression or anxiety | yes | 48 | 98 | 28 | 9,056 | 0 | 9,104 | 126 | 1.38 |
|  |  | no | 55 | 73 | 20 | 14,300 | 0 | 14,355 | 93 | 0.65 |
|  | Questionnaire type | online | 63 | 89 | 25 | 16,202 | 0 | 16,265 | 114 | 0.70 |
|  |  | paper | 40 | 82 | 23 | 7,154 | 0 | 7,194 | 105 | 1.46 |
|  | Gender | male | 32 | 39 | 15 | 7,780 | 0 | 7,812 | 54 | 0.69 |
|  |  | female | 71 | 132 | 33 | 15,576 | 0 | 15,647 | 165 | 1.05 |
|  | Currently married | yes | 50 | 111 | 24 | 16,312 | 0 | 16,362 | 135 | 0.83 |
|  |  | no | 53 | 60 | 24 | 7,044 | 0 | 7,097 | 84 | 1.18 |
|  | Current pain | yes | 103 | 171 | 47 | 19,836 | 0 | 19,939 | 218 | 1.09 |
|  |  | no | 0 | 0 | 1 | 3,520 | 0 | 3,520 | 1 | 0.03 |
|  | Tertiary qualification | yes | 32 | 77 | 23 | 12,199 | 0 | 12,231 | 100 | 0.82 |
|  |  | no | 71 | 94 | 25 | 11,157 | 0 | 11,228 | 119 | 1.06 |
| Oxycodone | Current smoker | yes | 84 | 54 | 46 | 1,517 | 0 | 1,601 | 100 | 6.25 |
|  |  | no | 765 | 450 | 599 | 20,163 | 0 | 20,928 | 1,049 | 5.01 |
|  | Current depression or anxiety | yes | 485 | 291 | 285 | 8,169 | 0 | 8,654 | 576 | 6.66 |
|  |  | no | 364 | 213 | 360 | 13,511 | 0 | 13,875 | 573 | 4.13 |
|  | Questionnaire type | online | 477 | 292 | 408 | 15,202 | 0 | 15,679 | 700 | 4.46 |
|  |  | paper | 372 | 212 | 237 | 6,478 | 0 | 6,850 | 449 | 6.55 |
|  | Gender | male | 224 | 159 | 197 | 7,286 | 0 | 7,510 | 356 | 4.74 |
|  |  | female | 625 | 345 | 448 | 14,394 | 0 | 15,019 | 793 | 5.28 |
|  | Currently married | yes | 481 | 339 | 432 | 15,245 | 0 | 15,726 | 771 | 4.90 |
|  |  | no | 368 | 165 | 213 | 6,435 | 0 | 6,803 | 378 | 5.56 |
|  | Current pain | yes | 844 | 501 | 599 | 18,213 | 0 | 19,057 | 1,100 | 5.77 |
|  |  | no | 5 | 3 | 46 | 3,467 | 0 | 3,472 | 49 | 1.41 |
|  | Tertiary qualification | yes | 369 | 237 | 301 | 11,424 | 0 | 11,793 | 538 | 4.56 |
|  |  | no | 480 | 267 | 344 | 10,256 | 0 | 10,736 | 611 | 5.69 |
| Penicillamine | Current smoker | yes | 0 | 0 | 0 | 1,668 | 33 | 1,668 | 0 | 0.00 |
|  |  | no | 17 | 7 | 0 | 21,699 | 254 | 21,716 | 7 | 0.03 |
|  | Current depression or anxiety | yes | 8 | 1 | 0 | 9,074 | 147 | 9,082 | 1 | 0.01 |
|  |  | no | 9 | 6 | 0 | 14,293 | 140 | 14,302 | 6 | 0.04 |
|  | Questionnaire type | online | 4 | 1 | 0 | 16,219 | 155 | 16,223 | 1 | 0.01 |
|  |  | paper | 13 | 6 | 0 | 7,148 | 132 | 7,161 | 6 | 0.08 |
|  | Gender | male | 0 | 6 | 0 | 7,725 | 135 | 7,725 | 6 | 0.08 |
|  |  | female | 17 | 1 | 0 | 15,642 | 152 | 15,659 | 1 | 0.01 |
|  | Currently married | yes | 3 | 6 | 0 | 16,310 | 178 | 16,313 | 6 | 0.04 |
|  |  | no | 14 | 1 | 0 | 7,057 | 109 | 7,071 | 1 | 0.01 |
|  | Current pain | yes | 17 | 7 | 0 | 19,872 | 261 | 19,889 | 7 | 0.04 |
|  |  | no | 0 | 0 | 0 | 3,495 | 26 | 3,495 | 0 | 0.00 |
|  | Tertiary qualification | yes | 0 | 6 | 0 | 12,220 | 105 | 12,220 | 6 | 0.05 |
|  |  | no | 17 | 1 | 0 | 11,147 | 182 | 11,164 | 1 | 0.01 |
| Piroxicam | Current smoker | yes | 4 | 3 | 6 | 1,688 | 0 | 1,692 | 9 | 0.53 |
|  |  | no | 147 | 74 | 38 | 21,718 | 0 | 21,865 | 112 | 0.51 |
|  | Current depression or anxiety | yes | 63 | 34 | 14 | 9,119 | 0 | 9,182 | 48 | 0.52 |
|  |  | no | 88 | 43 | 30 | 14,287 | 0 | 14,375 | 73 | 0.51 |
|  | Questionnaire type | online | 112 | 50 | 24 | 16,193 | 0 | 16,305 | 74 | 0.45 |
|  |  | paper | 39 | 27 | 20 | 7,213 | 0 | 7,252 | 47 | 0.65 |
|  | Gender | male | 42 | 35 | 15 | 7,774 | 0 | 7,816 | 50 | 0.64 |
|  |  | female | 109 | 42 | 29 | 15,632 | 0 | 15,741 | 71 | 0.45 |
|  | Currently married | yes | 104 | 52 | 27 | 16,314 | 0 | 16,418 | 79 | 0.48 |
|  |  | no | 47 | 25 | 17 | 7,092 | 0 | 7,139 | 42 | 0.59 |
|  | Current pain | yes | 141 | 70 | 43 | 19,903 | 0 | 20,044 | 113 | 0.56 |
|  |  | no | 10 | 7 | 1 | 3,503 | 0 | 3,513 | 8 | 0.23 |
|  | Tertiary qualification | yes | 83 | 44 | 20 | 12,184 | 0 | 12,267 | 64 | 0.52 |
|  |  | no | 68 | 33 | 24 | 11,222 | 0 | 11,290 | 57 | 0.50 |
| Prednisolone/Prednisone | Current smoker | yes | 378 | 132 | 111 | 1,079 | 1 | 1,457 | 243 | 16.68 |
|  |  | no | 4,439 | 1,296 | 1,140 | 14,965 | 137 | 19,404 | 2,436 | 12.55 |
|  | Current depression or anxiety | yes | 2,102 | 630 | 527 | 5,933 | 38 | 8,035 | 1,157 | 14.40 |
|  |  | no | 2,715 | 798 | 724 | 10,111 | 100 | 12,826 | 1,522 | 11.87 |
|  | Questionnaire type | online | 2,894 | 914 | 863 | 11,593 | 115 | 14,487 | 1,777 | 12.27 |
|  |  | paper | 1,923 | 514 | 388 | 4,451 | 23 | 6,374 | 902 | 14.15 |
|  | Gender | male | 1,418 | 442 | 345 | 5,603 | 58 | 7,021 | 787 | 11.21 |
|  |  | female | 3,399 | 986 | 906 | 10,441 | 80 | 13,840 | 1,892 | 13.67 |
|  | Currently married | yes | 3,202 | 985 | 868 | 11,325 | 117 | 14,527 | 1,853 | 12.76 |
|  |  | no | 1,615 | 443 | 383 | 4,719 | 21 | 6,334 | 826 | 13.04 |
|  | Current pain | yes | 4,531 | 1,323 | 1,127 | 13,077 | 99 | 17,608 | 2,450 | 13.91 |
|  |  | no | 286 | 105 | 124 | 2,967 | 39 | 3,253 | 229 | 7.04 |
|  | Tertiary qualification | yes | 2,234 | 727 | 621 | 8,669 | 80 | 10,903 | 1,348 | 12.36 |
|  |  | no | 2,583 | 701 | 630 | 7,375 | 58 | 9,958 | 1,331 | 13.37 |
| Rituximab | Current smoker | yes | 12 | 34 | 5 | 1,642 | 8 | 1,654 | 39 | 2.36 |
|  |  | no | 342 | 293 | 62 | 21,217 | 63 | 21,559 | 355 | 1.65 |
|  | Current depression or anxiety | yes | 163 | 137 | 37 | 8,847 | 46 | 9,010 | 174 | 1.93 |
|  |  | no | 191 | 190 | 30 | 14,012 | 25 | 14,203 | 220 | 1.55 |
|  | Questionnaire type | online | 253 | 201 | 48 | 15,839 | 38 | 16,092 | 249 | 1.55 |
|  |  | paper | 101 | 126 | 19 | 7,020 | 33 | 7,121 | 145 | 2.04 |
|  | Gender | male | 85 | 77 | 20 | 7,670 | 14 | 7,755 | 97 | 1.25 |
|  |  | female | 269 | 250 | 47 | 15,189 | 57 | 15,458 | 297 | 1.92 |
|  | Currently married | yes | 219 | 221 | 42 | 15,972 | 43 | 16,191 | 263 | 1.62 |
|  |  | no | 135 | 106 | 25 | 6,887 | 28 | 7,022 | 131 | 1.87 |
|  | Current pain | yes | 318 | 294 | 61 | 19,416 | 68 | 19,734 | 355 | 1.80 |
|  |  | no | 36 | 33 | 6 | 3,443 | 3 | 3,479 | 39 | 1.12 |
|  | Tertiary qualification | yes | 169 | 131 | 30 | 11,977 | 24 | 12,146 | 161 | 1.33 |
|  |  | no | 185 | 196 | 37 | 10,882 | 47 | 11,067 | 233 | 2.11 |
| Sulfasalazine | Current smoker | yes | 114 | 22 | 22 | 1,533 | 10 | 1,647 | 44 | 2.67 |
|  |  | no | 1,782 | 260 | 301 | 19,410 | 224 | 21,192 | 561 | 2.65 |
|  | Current depression or anxiety | yes | 725 | 123 | 165 | 8,124 | 93 | 8,849 | 288 | 3.25 |
|  |  | no | 1,171 | 159 | 158 | 12,819 | 141 | 13,990 | 317 | 2.27 |
|  | Questionnaire type | online | 1,235 | 199 | 225 | 14,565 | 155 | 15,800 | 424 | 2.68 |
|  |  | paper | 661 | 83 | 98 | 6,378 | 79 | 7,039 | 181 | 2.57 |
|  | Gender | male | 559 | 112 | 92 | 7,016 | 87 | 7,575 | 204 | 2.69 |
|  |  | female | 1,337 | 170 | 231 | 13,927 | 147 | 15,264 | 401 | 2.63 |
|  | Currently married | yes | 1,355 | 205 | 243 | 14,530 | 164 | 15,885 | 448 | 2.82 |
|  |  | no | 541 | 77 | 80 | 6,413 | 70 | 6,954 | 157 | 2.26 |
|  | Current pain | yes | 1,725 | 259 | 279 | 17,680 | 214 | 19,405 | 538 | 2.77 |
|  |  | no | 171 | 23 | 44 | 3,263 | 20 | 3,434 | 67 | 1.95 |
|  | Tertiary qualification | yes | 1,018 | 128 | 178 | 10,906 | 101 | 11,924 | 306 | 2.57 |
|  |  | no | 878 | 154 | 145 | 10,037 | 133 | 10,915 | 299 | 2.74 |
| Tocilizumab | Current smoker | yes | 101 | 17 | 18 | 1,564 | 1 | 1,665 | 35 | 2.10 |
|  |  | no | 1,061 | 82 | 132 | 20,689 | 13 | 21,750 | 214 | 0.98 |
|  | Current depression or anxiety | yes | 566 | 51 | 64 | 8,538 | 11 | 9,104 | 115 | 1.26 |
|  |  | no | 596 | 48 | 86 | 13,715 | 3 | 14,311 | 134 | 0.94 |
|  | Questionnaire type | online | 750 | 82 | 124 | 15,417 | 6 | 16,167 | 206 | 1.27 |
|  |  | paper | 412 | 17 | 26 | 6,836 | 8 | 7,248 | 43 | 0.59 |
|  | Gender | male | 218 | 21 | 32 | 7,593 | 2 | 7,811 | 53 | 0.68 |
|  |  | female | 944 | 78 | 118 | 14,660 | 12 | 15,604 | 196 | 1.26 |
|  | Currently married | yes | 710 | 60 | 108 | 15,607 | 12 | 16,317 | 168 | 1.03 |
|  |  | no | 452 | 39 | 42 | 6,646 | 2 | 7,098 | 81 | 1.14 |
|  | Current pain | yes | 1,072 | 94 | 139 | 18,838 | 14 | 19,910 | 233 | 1.17 |
|  |  | no | 90 | 5 | 11 | 3,415 | 0 | 3,505 | 16 | 0.46 |
|  | Tertiary qualification | yes | 559 | 54 | 93 | 11,623 | 2 | 12,182 | 147 | 1.21 |
|  |  | no | 603 | 45 | 57 | 10,630 | 12 | 11,233 | 102 | 0.91 |
| Tofacitinib citrate | Current smoker | yes | 20 | 4 | 4 | 1,673 | 0 | 1,693 | 8 | 0.47 |
|  |  | no | 554 | 71 | 118 | 21,226 | 8 | 21,780 | 189 | 0.87 |
|  | Current depression or anxiety | yes | 226 | 32 | 49 | 8,921 | 2 | 9,147 | 81 | 0.89 |
|  |  | no | 348 | 43 | 73 | 13,978 | 6 | 14,326 | 116 | 0.81 |
|  | Questionnaire type | online | 432 | 61 | 113 | 15,766 | 7 | 16,198 | 174 | 1.07 |
|  |  | paper | 142 | 14 | 9 | 7,133 | 1 | 7,275 | 23 | 0.32 |
|  | Gender | male | 116 | 16 | 19 | 7,713 | 2 | 7,829 | 35 | 0.45 |
|  |  | female | 458 | 59 | 103 | 15,186 | 6 | 15,644 | 162 | 1.04 |
|  | Currently married | yes | 438 | 60 | 86 | 15,907 | 6 | 16,345 | 146 | 0.89 |
|  |  | no | 136 | 15 | 36 | 6,992 | 2 | 7,128 | 51 | 0.72 |
|  | Current pain | yes | 495 | 69 | 107 | 19,481 | 5 | 19,976 | 176 | 0.88 |
|  |  | no | 79 | 6 | 15 | 3,418 | 3 | 3,497 | 21 | 0.60 |
|  | Tertiary qualification | yes | 334 | 47 | 55 | 11,891 | 4 | 12,225 | 102 | 0.83 |
|  |  | no | 240 | 28 | 67 | 11,008 | 4 | 11,248 | 95 | 0.84 |
| Tramadol | Current smoker | yes | 62 | 39 | 25 | 1,575 | 0 | 1,637 | 64 | 3.91 |
|  |  | no | 750 | 327 | 268 | 20,632 | 0 | 21,382 | 595 | 2.78 |
|  | Current depression or anxiety | yes | 382 | 174 | 127 | 8,547 | 0 | 8,929 | 301 | 3.37 |
|  |  | no | 430 | 192 | 166 | 13,660 | 0 | 14,090 | 358 | 2.54 |
|  | Questionnaire type | online | 497 | 231 | 174 | 15,477 | 0 | 15,974 | 405 | 2.54 |
|  |  | paper | 315 | 135 | 119 | 6,730 | 0 | 7,045 | 254 | 3.61 |
|  | Gender | male | 195 | 76 | 82 | 7,513 | 0 | 7,708 | 158 | 2.05 |
|  |  | female | 617 | 290 | 211 | 14,694 | 0 | 15,311 | 501 | 3.27 |
|  | Currently married | yes | 488 | 225 | 210 | 15,574 | 0 | 16,062 | 435 | 2.71 |
|  |  | no | 324 | 141 | 83 | 6,633 | 0 | 6,957 | 224 | 3.22 |
|  | Current pain | yes | 804 | 363 | 280 | 18,710 | 0 | 19,514 | 643 | 3.30 |
|  |  | no | 8 | 3 | 13 | 3,497 | 0 | 3,505 | 16 | 0.46 |
|  | Tertiary qualification | yes | 387 | 198 | 120 | 11,626 | 0 | 12,013 | 318 | 2.65 |
|  |  | no | 425 | 168 | 173 | 10,581 | 0 | 11,006 | 341 | 3.10 |
| Ustekinumab | Current smoker | yes | 0 | 0 | 1 | 1,700 | 0 | 1,700 | 1 | 0.06 |
|  |  | no | 63 | 15 | 8 | 21,886 | 5 | 21,949 | 23 | 0.10 |
|  | Current depression or anxiety | yes | 38 | 10 | 5 | 9,172 | 5 | 9,210 | 15 | 0.16 |
|  |  | no | 25 | 5 | 4 | 14,414 | 0 | 14,439 | 9 | 0.06 |
|  | Questionnaire type | online | 55 | 14 | 9 | 16,297 | 4 | 16,352 | 23 | 0.14 |
|  |  | paper | 8 | 1 | 0 | 7,289 | 1 | 7,297 | 1 | 0.01 |
|  | Gender | male | 19 | 3 | 3 | 7,841 | 0 | 7,860 | 6 | 0.08 |
|  |  | female | 44 | 12 | 6 | 15,745 | 5 | 15,789 | 18 | 0.11 |
|  | Currently married | yes | 47 | 8 | 6 | 16,433 | 3 | 16,480 | 14 | 0.08 |
|  |  | no | 16 | 7 | 3 | 7,153 | 2 | 7,169 | 10 | 0.14 |
|  | Current pain | yes | 59 | 15 | 7 | 20,071 | 5 | 20,130 | 22 | 0.11 |
|  |  | no | 4 | 0 | 2 | 3,515 | 0 | 3,519 | 2 | 0.06 |
|  | Tertiary qualification | yes | 41 | 11 | 6 | 12,270 | 3 | 12,311 | 17 | 0.14 |
|  |  | no | 22 | 4 | 3 | 11,316 | 2 | 11,338 | 7 | 0.06 |
| ^*^TP+TN; ^†^FP+FN; | | | | | | | | | | |
| Abbreviations: FN: False Negative; FP: False Positive; IRSAD SA: Index of Relative Socio-economic Advantage and Disadvantage Statistical Area; N/A: ‘Don’t know’ response in non-binary question; TN: True Negative; TP: True Positive. | | | | | | | | | | |

# Supplementary Table S4. Frequency, agreement, sensitivity, positive predictive value and negative predictive value of rheumatology-related medication self-reports compared with PBS prescription pharmaceutical claims data (Australian reference standard) using an exposure window of 1 month (30 days) prior to questionnaire submission date for the Australian Rheumatology Association Database (ARAD) cohort (questionnaire period: 2012-2023; PBS supply period: 2011 – 2023)

| **Medication name** | **Class** | **Prescription-only** | **TN** | **FN** | **FP** | **TP** | **N/A** | **Kappa (95% CI)** | **Sensitivity (95% CI)** | **PPV (95% CI)** | **NPV (95% CI)** |
| --- | --- | --- | --- | --- | --- | --- | --- | --- | --- | --- | --- |
| Prednisolone/Prednisone | Glucocorticoid | Yes | 17,029 | 524 | 3,182 | 3,119 | 139 | 0.54 (0.52-0.55) | 0.86 (0.84-0.87) | 0.5 (0.48-0.51) | 0.97 (0.97-0.97) |
| Aspirin | Non-opioid analgesic | No | 21,626 | 116 | 2,138 | 113 | 0 | 0.08 (0.04-0.11) | 0.49 (0.43-0.56) | 0.05 (0.04-0.06) | 0.99 (0.99-1) |
| Celecoxib | Non-opioid analgesic | Yes | 21,688 | 117 | 1,135 | 1,053 | 0 | 0.6 (0.58-0.62) | 0.9 (0.88-0.92) | 0.48 (0.46-0.5) | 0.99 (0.99-1) |
| Diclofenac | Non-opioid analgesic | No | 22,991 | 48 | 620 | 334 | 0 | 0.49 (0.45-0.53) | 0.87 (0.84-0.91) | 0.35 (0.32-0.38) | 1 (1-1) |
| Ibuprofen | Non-opioid analgesic | No | 21,490 | 23 | 2,350 | 130 | 0 | 0.09 (0.05-0.12) | 0.85 (0.78-0.9) | 0.05 (0.04-0.06) | 1 (1-1) |
| Indometacin | Non-opioid analgesic | Yes | 23,693 | 25 | 177 | 98 | 0 | 0.49 (0.42-0.56) | 0.8 (0.71-0.86) | 0.36 (0.3-0.42) | 1 (1-1) |
| Ketoprofen | Non-opioid analgesic | Yes | 23,702 | 11 | 113 | 167 | 0 | 0.73 (0.68-0.77) | 0.94 (0.89-0.97) | 0.6 (0.54-0.65) | 1 (1-1) |
| Meloxicam | Non-opioid analgesic | Yes | 21,676 | 138 | 1,172 | 1,007 | 0 | 0.58 (0.56-0.6) | 0.88 (0.86-0.9) | 0.46 (0.44-0.48) | 0.99 (0.99-0.99) |
| Naproxen | Non-opioid analgesic | No | 22,572 | 49 | 757 | 615 | 0 | 0.59 (0.56-0.62) | 0.93 (0.9-0.94) | 0.45 (0.42-0.48) | 1 (1-1) |
| Paracetamol | Non-opioid analgesic | No | 13,196 | 330 | 9,591 | 876 | 0 | 0.07 (0.05-0.08) | 0.73 (0.7-0.75) | 0.08 (0.08-0.09) | 0.98 (0.97-0.98) |
| Piroxicam | Non-opioid analgesic | Yes | 23,729 | 14 | 154 | 96 | 0 | 0.53 (0.46-0.6) | 0.87 (0.8-0.93) | 0.38 (0.32-0.45) | 1 (1-1) |
| Morphine | Opioid analgesic | Yes | 23,693 | 25 | 186 | 89 | 0 | 0.45 (0.38-0.53) | 0.78 (0.69-0.85) | 0.32 (0.27-0.38) | 1 (1-1) |
| Oxycodone | Opioid analgesic | Yes | 22,378 | 259 | 747 | 609 | 0 | 0.53 (0.5-0.56) | 0.7 (0.67-0.73) | 0.45 (0.42-0.48) | 0.99 (0.99-0.99) |
| Paracetamol & codeine | Opioid analgesic | No | 19,970 | 238 | 2,942 | 843 | 0 | 0.3 (0.27-0.32) | 0.78 (0.75-0.8) | 0.22 (0.21-0.24) | 0.99 (0.99-0.99) |
| Tramadol | Opioid analgesic | Yes | 22,693 | 117 | 581 | 602 | 0 | 0.62 (0.59-0.65) | 0.84 (0.81-0.86) | 0.51 (0.48-0.54) | 0.99 (0.99-1) |
| Abatacept - infusion | b/tsDMARD | Yes | 23,483 | 22 | 137 | 321 | 30 | 0.8 (0.77-0.83) | 0.94 (0.9-0.96) | 0.7 (0.66-0.74) | 1 (1-1) |
| Abatacept - injection | b/tsDMARD | Yes | 23,078 | 128 | 157 | 628 | 2 | 0.81 (0.79-0.83) | 0.83 (0.8-0.86) | 0.8 (0.77-0.83) | 0.99 (0.99-1) |
| Adalimumab | b/tsDMARD | Yes | 18,182 | 341 | 957 | 4,439 | 74 | 0.84 (0.83-0.85) | 0.93 (0.92-0.94) | 0.82 (0.81-0.83) | 0.98 (0.98-0.98) |
| Certolizumab pegol | b/tsDMARD | Yes | 23,346 | 54 | 103 | 483 | 7 | 0.86 (0.83-0.88) | 0.9 (0.87-0.92) | 0.82 (0.79-0.85) | 1 (1-1) |
| Etanercept | b/tsDMARD | Yes | 18,548 | 299 | 843 | 4,268 | 35 | 0.85 (0.84-0.86) | 0.93 (0.93-0.94) | 0.84 (0.82-0.85) | 0.98 (0.98-0.99) |
| Golimumab | b/tsDMARD | Yes | 22,376 | 111 | 262 | 1,240 | 4 | 0.86 (0.85-0.88) | 0.92 (0.9-0.93) | 0.83 (0.81-0.84) | 1 (0.99-1) |
| Infliximab | b/tsDMARD | Yes | 23,136 | 7 | 331 | 410 | 109 | 0.7 (0.67-0.73) | 0.98 (0.97-0.99) | 0.55 (0.52-0.59) | 1 (1-1) |
| Rituximab | b/tsDMARD | Yes | 23,231 | 9 | 606 | 75 | 72 | 0.19 (0.13-0.25) | 0.89 (0.81-0.95) | 0.11 (0.09-0.14) | 1 (1-1) |
| Tocilizumab | b/tsDMARD | Yes | 22,585 | 112 | 239 | 1,042 | 15 | 0.85 (0.83-0.86) | 0.9 (0.88-0.92) | 0.81 (0.79-0.83) | 1 (0.99-1) |
| Tofacitinib citrate | b/tsDMARD | Yes | 23,255 | 80 | 176 | 474 | 8 | 0.78 (0.76-0.81) | 0.86 (0.82-0.88) | 0.73 (0.69-0.76) | 1 (1-1) |
| Ustekinumab | b/tsDMARD | Yes | 23,906 | 4 | 54 | 24 | 5 | 0.45 (0.31-0.59) | 0.86 (0.67-0.96) | 0.31 (0.21-0.42) | 1 (1-1) |
| Azathioprine | csDMARD | Yes | 23,569 | 9 | 94 | 78 | 243 | 0.6 (0.52-0.68) | 0.9 (0.81-0.95) | 0.45 (0.38-0.53) | 1 (1-1) |
| Cyclosporin | csDMARD | Yes | 23,659 | 5 | 36 | 52 | 241 | 0.72 (0.63-0.8) | 0.91 (0.81-0.97) | 0.59 (0.48-0.69) | 1 (1-1) |
| Gold - intramuscular | csDMARD | Yes | 23,812 | 1 | 32 | 7 | 141 | 0.3 (0.06-0.54) | 0.88 (0.47-1) | 0.18 (0.08-0.34) | 1 (1-1) |
| Hydroxychloroquine | csDMARD | Yes | 20,404 | 78 | 2,113 | 1,187 | 211 | 0.48 (0.46-0.5) | 0.94 (0.92-0.95) | 0.36 (0.34-0.38) | 1 (1-1) |
| Leflunomide | csDMARD | Yes | 20,913 | 132 | 892 | 1,840 | 216 | 0.76 (0.74-0.77) | 0.93 (0.92-0.94) | 0.67 (0.66-0.69) | 0.99 (0.99-0.99) |
| Methotrexate - injection | csDMARD | Yes | 22,715 | 51 | 580 | 476 | 171 | 0.59 (0.56-0.62) | 0.9 (0.87-0.93) | 0.45 (0.42-0.48) | 1 (1-1) |
| Methotrexate - oral | csDMARD | Yes | 11,658 | 131 | 9,905 | 2,197 | 102 | 0.17 (0.16-0.18) | 0.94 (0.93-0.95) | 0.18 (0.17-0.19) | 0.99 (0.99-0.99) |
| Penicillamine | csDMARD | Yes | 23,681 | 0 | 16 | 8 | 288 | 0.5 (0.25-0.74) | 1 (0.63-1) | 0.33 (0.16-0.55) | 1 (1-1) |
| Sulfasalazine | csDMARD | Yes | 21,499 | 62 | 1,258 | 940 | 234 | 0.56 (0.54-0.59) | 0.94 (0.92-0.95) | 0.43 (0.41-0.45) | 1 (1-1) |
| Abbreviations: b/tsDMARD: biological or targeted synthetic disease-modifying antirheumatic drug; CI: Confidence Interval; csDMARD: conventional synthetic disease-modifying antirheumatic drug; FN: False Negative; FP: False Positive; N/A: ‘Don’t know’ response in non-binary question; NPV: Negative Predictive Value; PPV: Positive Predictive Value; TN: True Negative; TP: True Positive | | | | | | | | | | | |

# Supplementary Table S5. Frequency, agreement, sensitivity, positive predictive value and negative predictive value of rheumatology-related medication self-reports compared with PBS prescription pharmaceutical claims data (Australian reference standard) using an exposure window of 3 months (90 days) prior to questionnaire submission date for the Australian Rheumatology Association Database (ARAD) cohort (questionnaire period: 2012-2023; PBS supply period: 2011 – 2023)

| **Medication name** | **Class** | **Prescription-only** | **TN** | **FN** | **FP** | **TP** | **N/A** | **Kappa (95% CI)** | **Sensitivity (95% CI)** | **PPV (95% CI)** | **NPV (95% CI)** |
| --- | --- | --- | --- | --- | --- | --- | --- | --- | --- | --- | --- |
| Prednisolone/Prednisone | Glucocorticoid | Yes | 16,288 | 1,265 | 1,445 | 4,856 | 139 | 0.71 (0.69-0.72) | 0.79 (0.78-0.8) | 0.77 (0.76-0.78) | 0.93 (0.92-0.93) |
| Aspirin | Non-opioid analgesic | No | 21,526 | 216 | 2,020 | 231 | 0 | 0.14 (0.11-0.18) | 0.52 (0.47-0.56) | 0.1 (0.09-0.12) | 0.99 (0.99-0.99) |
| Celecoxib | Non-opioid analgesic | Yes | 21,520 | 285 | 647 | 1,541 | 0 | 0.75 (0.73-0.76) | 0.84 (0.83-0.86) | 0.7 (0.68-0.72) | 0.99 (0.99-0.99) |
| Diclofenac | Non-opioid analgesic | No | 22,910 | 129 | 440 | 514 | 0 | 0.63 (0.6-0.66) | 0.8 (0.77-0.83) | 0.54 (0.51-0.57) | 0.99 (0.99-1) |
| Ibuprofen | Non-opioid analgesic | No | 21,452 | 61 | 2,262 | 218 | 0 | 0.14 (0.11-0.17) | 0.78 (0.73-0.83) | 0.09 (0.08-0.1) | 1 (1-1) |
| Indometacin | Non-opioid analgesic | Yes | 23,658 | 60 | 115 | 160 | 0 | 0.64 (0.59-0.7) | 0.73 (0.66-0.78) | 0.58 (0.52-0.64) | 1 (1-1) |
| Ketoprofen | Non-opioid analgesic | Yes | 23,684 | 29 | 74 | 206 | 0 | 0.8 (0.76-0.84) | 0.88 (0.83-0.92) | 0.74 (0.68-0.79) | 1 (1-1) |
| Meloxicam | Non-opioid analgesic | Yes | 21,471 | 343 | 719 | 1,460 | 0 | 0.71 (0.69-0.73) | 0.81 (0.79-0.83) | 0.67 (0.65-0.69) | 0.98 (0.98-0.99) |
| Naproxen | Non-opioid analgesic | No | 22,471 | 150 | 478 | 894 | 0 | 0.73 (0.71-0.75) | 0.86 (0.83-0.88) | 0.65 (0.63-0.68) | 0.99 (0.99-0.99) |
| Paracetamol | Non-opioid analgesic | No | 12,902 | 624 | 8,969 | 1,498 | 0 | 0.11 (0.09-0.12) | 0.71 (0.69-0.73) | 0.14 (0.14-0.15) | 0.95 (0.95-0.96) |
| Piroxicam | Non-opioid analgesic | Yes | 23,714 | 29 | 105 | 145 | 0 | 0.68 (0.63-0.74) | 0.83 (0.77-0.89) | 0.58 (0.52-0.64) | 1 (1-1) |
| Morphine | Opioid analgesic | Yes | 23,684 | 34 | 177 | 98 | 0 | 0.48 (0.41-0.55) | 0.74 (0.66-0.81) | 0.36 (0.3-0.42) | 1 (1-1) |
| Oxycodone | Opioid analgesic | Yes | 21,987 | 650 | 507 | 849 | 0 | 0.57 (0.54-0.59) | 0.57 (0.54-0.59) | 0.63 (0.6-0.65) | 0.97 (0.97-0.97) |
| Paracetamol & codeine | Opioid analgesic | No | 19,586 | 622 | 2,462 | 1,323 | 0 | 0.4 (0.38-0.42) | 0.68 (0.66-0.7) | 0.35 (0.33-0.36) | 0.97 (0.97-0.97) |
| Tramadol | Opioid analgesic | Yes | 22,517 | 293 | 368 | 815 | 0 | 0.7 (0.67-0.72) | 0.74 (0.71-0.76) | 0.69 (0.66-0.72) | 0.99 (0.99-0.99) |
| Abatacept - infusion | b/tsDMARD | Yes | 23,472 | 33 | 91 | 367 | 30 | 0.85 (0.83-0.88) | 0.92 (0.89-0.94) | 0.8 (0.76-0.84) | 1 (1-1) |
| Abatacept - injection | b/tsDMARD | Yes | 23,052 | 154 | 79 | 706 | 2 | 0.85 (0.83-0.87) | 0.82 (0.79-0.85) | 0.9 (0.88-0.92) | 0.99 (0.99-0.99) |
| Adalimumab | b/tsDMARD | Yes | 18,100 | 423 | 277 | 5,119 | 74 | 0.92 (0.91-0.92) | 0.92 (0.92-0.93) | 0.95 (0.94-0.95) | 0.98 (0.97-0.98) |
| Certolizumab pegol | b/tsDMARD | Yes | 23,322 | 78 | 45 | 541 | 7 | 0.9 (0.88-0.91) | 0.87 (0.85-0.9) | 0.92 (0.9-0.94) | 1 (1-1) |
| Etanercept | b/tsDMARD | Yes | 18,482 | 365 | 221 | 4,890 | 35 | 0.93 (0.92-0.93) | 0.93 (0.92-0.94) | 0.96 (0.95-0.96) | 0.98 (0.98-0.98) |
| Golimumab | b/tsDMARD | Yes | 22,342 | 145 | 105 | 1,397 | 4 | 0.91 (0.9-0.92) | 0.91 (0.89-0.92) | 0.93 (0.92-0.94) | 0.99 (0.99-0.99) |
| Infliximab | b/tsDMARD | Yes | 23,117 | 26 | 58 | 683 | 109 | 0.94 (0.93-0.95) | 0.96 (0.95-0.98) | 0.92 (0.9-0.94) | 1 (1-1) |
| Rituximab | b/tsDMARD | Yes | 23,224 | 16 | 510 | 171 | 72 | 0.39 (0.33-0.44) | 0.91 (0.86-0.95) | 0.25 (0.22-0.29) | 1 (1-1) |
| Tocilizumab | b/tsDMARD | Yes | 22,544 | 153 | 106 | 1,175 | 15 | 0.9 (0.88-0.91) | 0.88 (0.87-0.9) | 0.92 (0.9-0.93) | 0.99 (0.99-0.99) |
| Tofacitinib citrate | b/tsDMARD | Yes | 23,232 | 103 | 88 | 562 | 8 | 0.85 (0.83-0.87) | 0.85 (0.82-0.87) | 0.86 (0.84-0.89) | 1 (0.99-1) |
| Ustekinumab | b/tsDMARD | Yes | 23,900 | 10 | 21 | 57 | 5 | 0.79 (0.71-0.86) | 0.85 (0.74-0.93) | 0.73 (0.62-0.82) | 1 (1-1) |
| Azathioprine | csDMARD | Yes | 23,560 | 18 | 43 | 129 | 243 | 0.81 (0.76-0.86) | 0.88 (0.81-0.93) | 0.75 (0.68-0.81) | 1 (1-1) |
| Cyclosporin | csDMARD | Yes | 23,651 | 13 | 15 | 73 | 241 | 0.84 (0.78-0.9) | 0.85 (0.76-0.92) | 0.83 (0.73-0.9) | 1 (1-1) |
| Gold - intramuscular | csDMARD | Yes | 23,810 | 3 | 19 | 20 | 141 | 0.64 (0.5-0.79) | 0.87 (0.66-0.97) | 0.51 (0.35-0.68) | 1 (1-1) |
| Hydroxychloroquine | csDMARD | Yes | 20,286 | 196 | 831 | 2,469 | 211 | 0.8 (0.79-0.82) | 0.93 (0.92-0.94) | 0.75 (0.73-0.76) | 0.99 (0.99-0.99) |
| Leflunomide | csDMARD | Yes | 20,734 | 311 | 402 | 2,330 | 216 | 0.85 (0.84-0.86) | 0.88 (0.87-0.89) | 0.85 (0.84-0.87) | 0.99 (0.98-0.99) |
| Methotrexate - injection | csDMARD | Yes | 22,667 | 99 | 332 | 724 | 171 | 0.76 (0.74-0.78) | 0.88 (0.86-0.9) | 0.69 (0.66-0.71) | 1 (0.99-1) |
| Methotrexate - oral | csDMARD | Yes | 11,459 | 330 | 6,628 | 5,474 | 102 | 0.42 (0.41-0.43) | 0.94 (0.94-0.95) | 0.45 (0.44-0.46) | 0.97 (0.97-0.97) |
| Penicillamine | csDMARD | Yes | 23,681 | 0 | 11 | 13 | 288 | 0.7 (0.53-0.88) | 1 (0.75-1) | 0.54 (0.33-0.74) | 1 (1-1) |
| Sulfasalazine | csDMARD | Yes | 21,382 | 179 | 502 | 1,696 | 234 | 0.82 (0.8-0.83) | 0.9 (0.89-0.92) | 0.77 (0.75-0.79) | 0.99 (0.99-0.99) |
| Abbreviations: b/tsDMARD: biological or targeted synthetic disease-modifying antirheumatic drug; CI: Confidence Interval; csDMARD: conventional synthetic disease-modifying antirheumatic drug; FN: False Negative; FP: False Positive; N/A: ‘Don’t know’ response in non-binary question; NPV: Negative Predictive Value; PPV: Positive Predictive Value; TN: True Negative; TP: True Positive | | | | | | | | | | | |

# Supplementary Table S6. Frequency, agreement, sensitivity, positive predictive value and negative predictive value of rheumatology-related medication self-reports compared with PBS prescription pharmaceutical claims data (Australian reference standard) using an exposure window of 6 months (182 days) prior to questionnaire submission date for the Australian Rheumatology Association Database (ARAD) cohort (questionnaire period: 2012-2023; PBS supply period: 2011 – 2023).

| **Medication name** | **Class** | **Prescription-only** | **TN** | **FN** | **FP** | **TP** | **N/A** | **Kappa (95% CI)** | **Sensitivity (95% CI)** | **PPV (95% CI)** | **NPV (95% CI)** |
| --- | --- | --- | --- | --- | --- | --- | --- | --- | --- | --- | --- |
| Prednisolone/Prednisone | Glucocorticoid | Yes | 15,408 | 2,145 | 990 | 5,311 | 139 | 0.68 (0.67-0.69) | 0.71 (0.7-0.72) | 0.84 (0.83-0.85) | 0.88 (0.87-0.88) |
| Aspirin | Non-opioid analgesic | No | 21,459 | 283 | 1,926 | 325 | 0 | 0.2 (0.16-0.23) | 0.53 (0.49-0.57) | 0.14 (0.13-0.16) | 0.99 (0.99-0.99) |
| Celecoxib | Non-opioid analgesic | Yes | 21,259 | 546 | 480 | 1,708 | 0 | 0.75 (0.73-0.76) | 0.76 (0.74-0.78) | 0.78 (0.76-0.8) | 0.97 (0.97-0.98) |
| Diclofenac | Non-opioid analgesic | No | 22,819 | 220 | 354 | 600 | 0 | 0.66 (0.64-0.69) | 0.73 (0.7-0.76) | 0.63 (0.6-0.66) | 0.99 (0.99-0.99) |
| Ibuprofen | Non-opioid analgesic | No | 21,379 | 134 | 2,186 | 294 | 0 | 0.18 (0.15-0.21) | 0.69 (0.64-0.73) | 0.12 (0.11-0.13) | 0.99 (0.99-0.99) |
| Indometacin | Non-opioid analgesic | Yes | 23,617 | 101 | 88 | 187 | 0 | 0.66 (0.61-0.71) | 0.65 (0.59-0.7) | 0.68 (0.62-0.73) | 1 (0.99-1) |
| Ketoprofen | Non-opioid analgesic | Yes | 23,661 | 52 | 61 | 219 | 0 | 0.79 (0.75-0.83) | 0.81 (0.76-0.85) | 0.78 (0.73-0.83) | 1 (1-1) |
| Meloxicam | Non-opioid analgesic | Yes | 21,186 | 628 | 507 | 1,672 | 0 | 0.72 (0.7-0.74) | 0.73 (0.71-0.75) | 0.77 (0.75-0.78) | 0.97 (0.97-0.97) |
| Naproxen | Non-opioid analgesic | No | 22,312 | 309 | 340 | 1,032 | 0 | 0.75 (0.73-0.77) | 0.77 (0.75-0.79) | 0.75 (0.73-0.77) | 0.99 (0.98-0.99) |
| Paracetamol | Non-opioid analgesic | No | 12,696 | 830 | 8,599 | 1,868 | 0 | 0.13 (0.11-0.14) | 0.69 (0.67-0.71) | 0.18 (0.17-0.19) | 0.94 (0.93-0.94) |
| Piroxicam | Non-opioid analgesic | Yes | 23,699 | 44 | 79 | 171 | 0 | 0.73 (0.69-0.78) | 0.8 (0.74-0.85) | 0.68 (0.62-0.74) | 1 (1-1) |
| Morphine | Opioid analgesic | Yes | 23,670 | 48 | 172 | 103 | 0 | 0.48 (0.41-0.55) | 0.68 (0.6-0.76) | 0.37 (0.32-0.43) | 1 (1-1) |
| Oxycodone | Opioid analgesic | Yes | 21,468 | 1,169 | 386 | 970 | 0 | 0.52 (0.5-0.54) | 0.45 (0.43-0.47) | 0.72 (0.69-0.74) | 0.95 (0.95-0.95) |
| Paracetamol & codeine | Opioid analgesic | No | 19,049 | 1,159 | 2,134 | 1,651 | 0 | 0.42 (0.4-0.44) | 0.59 (0.57-0.61) | 0.44 (0.42-0.45) | 0.94 (0.94-0.95) |
| Tramadol | Opioid analgesic | Yes | 22,276 | 534 | 266 | 917 | 0 | 0.68 (0.66-0.7) | 0.63 (0.61-0.66) | 0.78 (0.75-0.8) | 0.98 (0.97-0.98) |
| Abatacept - infusion | b/tsDMARD | Yes | 23,458 | 47 | 82 | 376 | 30 | 0.85 (0.83-0.88) | 0.89 (0.85-0.92) | 0.82 (0.78-0.85) | 1 (1-1) |
| Abatacept - injection | b/tsDMARD | Yes | 23,017 | 189 | 67 | 718 | 2 | 0.84 (0.82-0.86) | 0.79 (0.76-0.82) | 0.91 (0.89-0.93) | 0.99 (0.99-0.99) |
| Adalimumab | b/tsDMARD | Yes | 18,008 | 515 | 211 | 5,185 | 74 | 0.91 (0.91-0.92) | 0.91 (0.9-0.92) | 0.96 (0.96-0.97) | 0.97 (0.97-0.97) |
| Certolizumab pegol | b/tsDMARD | Yes | 23,304 | 96 | 37 | 549 | 7 | 0.89 (0.87-0.91) | 0.85 (0.82-0.88) | 0.94 (0.91-0.96) | 1 (0.99-1) |
| Etanercept | b/tsDMARD | Yes | 18,412 | 435 | 162 | 4,949 | 35 | 0.93 (0.92-0.93) | 0.92 (0.91-0.93) | 0.97 (0.96-0.97) | 0.98 (0.97-0.98) |
| Golimumab | b/tsDMARD | Yes | 22,320 | 167 | 82 | 1,420 | 4 | 0.91 (0.9-0.92) | 0.89 (0.88-0.91) | 0.95 (0.93-0.96) | 0.99 (0.99-0.99) |
| Infliximab | b/tsDMARD | Yes | 23,112 | 31 | 41 | 700 | 109 | 0.95 (0.94-0.96) | 0.96 (0.94-0.97) | 0.94 (0.93-0.96) | 1 (1-1) |
| Rituximab | b/tsDMARD | Yes | 23,204 | 36 | 386 | 295 | 72 | 0.58 (0.53-0.62) | 0.89 (0.85-0.92) | 0.43 (0.4-0.47) | 1 (1-1) |
| Tocilizumab | b/tsDMARD | Yes | 22,509 | 188 | 96 | 1,185 | 15 | 0.89 (0.87-0.9) | 0.86 (0.84-0.88) | 0.93 (0.91-0.94) | 0.99 (0.99-0.99) |
| Tofacitinib citrate | b/tsDMARD | Yes | 23,213 | 122 | 76 | 574 | 8 | 0.85 (0.83-0.87) | 0.82 (0.79-0.85) | 0.88 (0.86-0.91) | 0.99 (0.99-1) |
| Ustekinumab | b/tsDMARD | Yes | 23,900 | 10 | 15 | 63 | 5 | 0.83 (0.77-0.9) | 0.86 (0.76-0.93) | 0.81 (0.7-0.89) | 1 (1-1) |
| Azathioprine | csDMARD | Yes | 23,548 | 30 | 33 | 139 | 243 | 0.81 (0.77-0.86) | 0.82 (0.76-0.88) | 0.81 (0.74-0.86) | 1 (1-1) |
| Cyclosporin | csDMARD | Yes | 23,643 | 21 | 12 | 76 | 241 | 0.82 (0.76-0.88) | 0.78 (0.69-0.86) | 0.86 (0.77-0.93) | 1 (1-1) |
| Gold - intramuscular | csDMARD | Yes | 23,802 | 11 | 14 | 25 | 141 | 0.67 (0.54-0.8) | 0.69 (0.52-0.84) | 0.64 (0.47-0.79) | 1 (1-1) |
| Hydroxychloroquine | csDMARD | Yes | 20,097 | 385 | 412 | 2,888 | 211 | 0.86 (0.85-0.87) | 0.88 (0.87-0.89) | 0.88 (0.86-0.89) | 0.98 (0.98-0.98) |
| Leflunomide | csDMARD | Yes | 20,527 | 518 | 291 | 2,441 | 216 | 0.84 (0.83-0.85) | 0.82 (0.81-0.84) | 0.89 (0.88-0.9) | 0.98 (0.97-0.98) |
| Methotrexate - injection | csDMARD | Yes | 22,600 | 166 | 241 | 815 | 171 | 0.79 (0.77-0.81) | 0.83 (0.81-0.85) | 0.77 (0.75-0.8) | 0.99 (0.99-0.99) |
| Methotrexate - oral | csDMARD | Yes | 11,153 | 636 | 3,568 | 8,534 | 102 | 0.65 (0.64-0.66) | 0.93 (0.93-0.94) | 0.71 (0.7-0.71) | 0.95 (0.94-0.95) |
| Penicillamine | csDMARD | Yes | 23,681 | 0 | 7 | 17 | 288 | 0.83 (0.7-0.96) | 1 (0.8-1) | 0.71 (0.49-0.87) | 1 (1-1) |
| Sulfasalazine | csDMARD | Yes | 21,233 | 328 | 292 | 1,906 | 234 | 0.85 (0.83-0.86) | 0.85 (0.84-0.87) | 0.87 (0.85-0.88) | 0.98 (0.98-0.99) |
| Abbreviations: b/tsDMARD: biological or targeted synthetic disease-modifying antirheumatic drug; CI: Confidence Interval; csDMARD: conventional synthetic disease-modifying antirheumatic drug; FN: False Negative; FP: False Positive; N/A: ‘Don’t know’ response in non-binary question; NPV: Negative Predictive Value; PPV: Positive Predictive Value; TN: True Negative; TP: True Positive | | | | | | | | | | | |

# Supplementary Table S7. Frequency, agreement, sensitivity, positive predictive value and negative predictive value of rheumatology-related medication self-reports compared with PBS prescription pharmaceutical claims data (Australian reference standard) using an exposure window of 12 months (365 days) prior to questionnaire submission date for the Australian Rheumatology Association Database (ARAD) cohort (questionnaire period: 2012-2023; PBS supply period: 2011 – 2023)

| **Medication name** | **Class** | **Prescription-only** | **TN** | **FN** | **FP** | **TP** | **N/A** | **Kappa (95% CI)** | **Sensitivity (95% CI)** | **PPV (95% CI)** | **NPV (95% CI)** |
| --- | --- | --- | --- | --- | --- | --- | --- | --- | --- | --- | --- |
| Prednisolone/Prednisone | Glucocorticoid | Yes | 14,028 | 3,525 | 705 | 5,596 | 139 | 0.6 (0.59-0.61) | 0.61 (0.6-0.62) | 0.89 (0.88-0.9) | 0.8 (0.79-0.81) |
| Aspirin | Non-opioid analgesic | No | 21,350 | 392 | 1,843 | 408 | 0 | 0.23 (0.2-0.26) | 0.51 (0.47-0.55) | 0.18 (0.17-0.2) | 0.98 (0.98-0.98) |
| Celecoxib | Non-opioid analgesic | Yes | 20,817 | 988 | 338 | 1,850 | 0 | 0.71 (0.69-0.72) | 0.65 (0.63-0.67) | 0.85 (0.83-0.86) | 0.95 (0.95-0.96) |
| Diclofenac | Non-opioid analgesic | No | 22,628 | 411 | 294 | 660 | 0 | 0.64 (0.61-0.66) | 0.62 (0.59-0.65) | 0.69 (0.66-0.72) | 0.98 (0.98-0.98) |
| Ibuprofen | Non-opioid analgesic | No | 21,259 | 254 | 2,123 | 357 | 0 | 0.2 (0.17-0.23) | 0.58 (0.54-0.62) | 0.14 (0.13-0.16) | 0.99 (0.99-0.99) |
| Indometacin | Non-opioid analgesic | Yes | 23,550 | 168 | 74 | 201 | 0 | 0.62 (0.57-0.67) | 0.54 (0.49-0.6) | 0.73 (0.67-0.78) | 0.99 (0.99-0.99) |
| Ketoprofen | Non-opioid analgesic | Yes | 23,636 | 77 | 47 | 233 | 0 | 0.79 (0.75-0.82) | 0.75 (0.7-0.8) | 0.83 (0.78-0.87) | 1 (1-1) |
| Meloxicam | Non-opioid analgesic | Yes | 20,740 | 1,074 | 338 | 1,841 | 0 | 0.69 (0.68-0.71) | 0.63 (0.61-0.65) | 0.84 (0.83-0.86) | 0.95 (0.95-0.95) |
| Naproxen | Non-opioid analgesic | No | 22,035 | 586 | 236 | 1,136 | 0 | 0.72 (0.7-0.74) | 0.66 (0.64-0.68) | 0.83 (0.81-0.85) | 0.97 (0.97-0.98) |
| Paracetamol | Non-opioid analgesic | No | 12,382 | 1,144 | 8,178 | 2,289 | 0 | 0.15 (0.13-0.16) | 0.67 (0.65-0.68) | 0.22 (0.21-0.23) | 0.92 (0.91-0.92) |
| Piroxicam | Non-opioid analgesic | Yes | 23,665 | 78 | 58 | 192 | 0 | 0.74 (0.69-0.78) | 0.71 (0.65-0.76) | 0.77 (0.71-0.82) | 1 (1-1) |
| Morphine | Opioid analgesic | Yes | 23,636 | 82 | 167 | 108 | 0 | 0.46 (0.39-0.53) | 0.57 (0.49-0.64) | 0.39 (0.33-0.45) | 1 (1-1) |
| Oxycodone | Opioid analgesic | Yes | 20,558 | 2,079 | 287 | 1,069 | 0 | 0.43 (0.41-0.45) | 0.34 (0.32-0.36) | 0.79 (0.77-0.81) | 0.91 (0.9-0.91) |
| Paracetamol & codeine | Opioid analgesic | No | 18,171 | 2,037 | 1,819 | 1,966 | 0 | 0.41 (0.39-0.43) | 0.49 (0.48-0.51) | 0.52 (0.5-0.54) | 0.9 (0.89-0.9) |
| Tramadol | Opioid analgesic | Yes | 21,871 | 939 | 192 | 991 | 0 | 0.61 (0.59-0.64) | 0.51 (0.49-0.54) | 0.84 (0.82-0.86) | 0.96 (0.96-0.96) |
| Abatacept - infusion | b/tsDMARD | Yes | 23,437 | 68 | 75 | 383 | 30 | 0.84 (0.81-0.87) | 0.85 (0.81-0.88) | 0.84 (0.8-0.87) | 1 (1-1) |
| Abatacept - injection | b/tsDMARD | Yes | 22,968 | 238 | 56 | 729 | 2 | 0.83 (0.81-0.85) | 0.75 (0.73-0.78) | 0.93 (0.91-0.95) | 0.99 (0.99-0.99) |
| Adalimumab | b/tsDMARD | Yes | 17,862 | 661 | 171 | 5,225 | 74 | 0.9 (0.9-0.91) | 0.89 (0.88-0.9) | 0.97 (0.96-0.97) | 0.96 (0.96-0.97) |
| Certolizumab pegol | b/tsDMARD | Yes | 23,271 | 129 | 30 | 556 | 7 | 0.87 (0.85-0.89) | 0.81 (0.78-0.84) | 0.95 (0.93-0.97) | 0.99 (0.99-1) |
| Etanercept | b/tsDMARD | Yes | 18,305 | 542 | 129 | 4,982 | 35 | 0.92 (0.91-0.92) | 0.9 (0.89-0.91) | 0.97 (0.97-0.98) | 0.97 (0.97-0.97) |
| Golimumab | b/tsDMARD | Yes | 22,258 | 229 | 67 | 1,435 | 4 | 0.9 (0.89-0.91) | 0.86 (0.84-0.88) | 0.96 (0.94-0.97) | 0.99 (0.99-0.99) |
| Infliximab | b/tsDMARD | Yes | 23,098 | 45 | 39 | 702 | 109 | 0.94 (0.93-0.95) | 0.94 (0.92-0.96) | 0.95 (0.93-0.96) | 1 (1-1) |
| Rituximab | b/tsDMARD | Yes | 23,172 | 68 | 327 | 354 | 72 | 0.63 (0.6-0.67) | 0.84 (0.8-0.87) | 0.52 (0.48-0.56) | 1 (1-1) |
| Tocilizumab | b/tsDMARD | Yes | 22,466 | 231 | 80 | 1,201 | 15 | 0.88 (0.87-0.89) | 0.84 (0.82-0.86) | 0.94 (0.92-0.95) | 0.99 (0.99-0.99) |
| Tofacitinib citrate | b/tsDMARD | Yes | 23,180 | 155 | 62 | 588 | 8 | 0.84 (0.82-0.86) | 0.79 (0.76-0.82) | 0.9 (0.88-0.93) | 0.99 (0.99-0.99) |
| Ustekinumab | b/tsDMARD | Yes | 23,894 | 16 | 11 | 67 | 5 | 0.83 (0.77-0.9) | 0.81 (0.71-0.89) | 0.86 (0.76-0.93) | 1 (1-1) |
| Azathioprine | csDMARD | Yes | 23,526 | 52 | 23 | 149 | 243 | 0.8 (0.75-0.84) | 0.74 (0.67-0.8) | 0.87 (0.81-0.91) | 1 (1-1) |
| Cyclosporin | csDMARD | Yes | 23,629 | 35 | 10 | 78 | 241 | 0.78 (0.71-0.84) | 0.69 (0.6-0.77) | 0.89 (0.8-0.94) | 1 (1-1) |
| Gold - intramuscular | csDMARD | Yes | 23,793 | 20 | 12 | 27 | 141 | 0.63 (0.5-0.76) | 0.57 (0.42-0.72) | 0.69 (0.52-0.83) | 1 (1-1) |
| Hydroxychloroquine | csDMARD | Yes | 19,743 | 739 | 271 | 3,029 | 211 | 0.83 (0.82-0.84) | 0.8 (0.79-0.82) | 0.92 (0.91-0.93) | 0.96 (0.96-0.97) |
| Leflunomide | csDMARD | Yes | 20,090 | 955 | 206 | 2,526 | 216 | 0.79 (0.77-0.8) | 0.73 (0.71-0.74) | 0.92 (0.91-0.93) | 0.95 (0.95-0.96) |
| Methotrexate - injection | csDMARD | Yes | 22,492 | 274 | 183 | 873 | 171 | 0.78 (0.76-0.8) | 0.76 (0.74-0.79) | 0.83 (0.8-0.85) | 0.99 (0.99-0.99) |
| Methotrexate - oral | csDMARD | Yes | 10,615 | 1,174 | 1,343 | 10,759 | 102 | 0.79 (0.78-0.8) | 0.9 (0.9-0.91) | 0.89 (0.88-0.89) | 0.9 (0.89-0.91) |
| Penicillamine | csDMARD | Yes | 23,680 | 1 | 6 | 18 | 288 | 0.84 (0.72-0.96) | 0.95 (0.74-1) | 0.75 (0.53-0.9) | 1 (1-1) |
| Sulfasalazine | csDMARD | Yes | 20,928 | 633 | 198 | 2,000 | 234 | 0.81 (0.8-0.82) | 0.76 (0.74-0.78) | 0.91 (0.9-0.92) | 0.97 (0.97-0.97) |
| Abbreviations: b/tsDMARD: biological or targeted synthetic disease-modifying antirheumatic drug; CI: Confidence Interval; csDMARD: conventional synthetic disease-modifying antirheumatic drug; FN: False Negative; FP: False Positive; N/A: ‘Don’t know’ response in non-binary question; NPV: Negative Predictive Value; PPV: Positive Predictive Value; TN: True Negative; TP: True Positive | | | | | | | | | | | |

# Supplementary Table S8. Univariate odds ratios (95% CI) for factors associated with discordant self-reported prescription medication use (false positive or false negative) in ARAD questionnaires using Australian PBS prescription claims data as reference standard

| **Drug** | | **Questionnaire variable** | | | | | | | | | | | |
| --- | --- | --- | --- | --- | --- | --- | --- | --- | --- | --- | --- | --- | --- |
| **Class** | **Medication name** | **Married^#^** | **Age^†^** | **Higher education^‡^** | **Disease duration^§^** | **Current smoker^**^** | **Socioeconomic status^##^** | **Female sex^††^** | **Online questionnaire^‡‡^** | **Currently depressed or anxious^§§^** | **Current pain^^^** | **Self-rated health^+^** | **HAQ score^^^^** |
| csDMARD | Hydroxychloroquine | 0.95 (0.81-1.12) | 1 (0.99-1.01) | 0.97 (0.83-1.13) | 0.98 (0.98-0.99)* | 1.09 (0.81-1.44) | 1 (1-1) | 1.75 (1.47-2.11)* | 1.27 (1.07-1.51)* | 1.45 (1.24-1.68)* | 1.99 (1.53-2.64)* | 0.99 (0.98-0.99)* | 1.38 (1.25-1.53)* |
|  | Leflunomide | 0.7 (0.6-0.83)* | 1.01 (1-1.01) | 0.82 (0.7-0.97)* | 0.98 (0.97-0.99)* | 1.8 (1.4-2.3)* | 1 (1-1) | 1.12 (0.94-1.33) | 0.76 (0.64-0.89)* | 1.6 (1.36-1.87)* | 1.48 (1.16-1.93)* | 0.98 (0.98-0.99)* | 1.44 (1.3-1.6)* |
|  | Methotrexate - injection | 0.83 (0.66-1.04) | 0.99 (0.98-1) | 1.26 (1.01-1.57)* | 0.98 (0.97-0.99)* | 0.79 (0.48-1.22) | 1 (1-1.01) | 0.98 (0.79-1.24) | 1.62 (1.26-2.11)* | 1.91 (1.54-2.38)* | 1.32 (0.96-1.87) | 0.98 (0.98-0.99)* | 1.47 (1.27-1.69)* |
|  | Methotrexate - oral | 0.82 (0.75-0.9)* | 1 (1-1) | 0.97 (0.89-1.06) | 0.99 (0.99-1)* | 1.18 (1-1.38)* | 1 (1-1) | 1.35 (1.23-1.49)* | 1.04 (0.94-1.14) | 1.2 (1.1-1.31)* | 1.24 (1.09-1.42)* | 0.99 (0.99-1)* | 1.19 (1.12-1.26)* |
|  | Sulfasalazine | 1.26 (1.04-1.53)* | 1 (0.99-1) | 0.9 (0.76-1.07) | 0.98 (0.97-0.99)* | 1.05 (0.74-1.43) | 1 (0.99-1) | 0.97 (0.81-1.16) | 0.96 (0.8-1.15) | 1.37 (1.15-1.63)* | 1.57 (1.2-2.1)* | 0.99 (0.99-0.99)* | 1.4 (1.25-1.57)* |
| b/tsDMARD | Abatacept - injection | 0.79 (0.6-1.07) | 1.02 (1.01-1.03)* | 0.87 (0.66-1.15) | 1 (0.99-1.01) | 0.83 (0.44-1.43) | 0.99 (0.99-1)* | 1.95 (1.4-2.79)* | 1.18 (0.87-1.61) | 1.34 (1.01-1.77)* | 2.6 (1.55-4.81)* | 0.98 (0.97-0.98)* | 1.63 (1.35-1.95)* |
|  | Adalimumab | 1.06 (0.89-1.28) | 0.98 (0.98-0.99)* | 1.25 (1.06-1.48)* | 0.99 (0.98-1)* | 1.66 (1.26-2.14)* | 1 (1-1.01)* | 0.75 (0.64-0.89)* | 2.23 (1.8-2.79)* | 1.02 (0.86-1.21) | 1.08 (0.86-1.39) | 0.99 (0.99-1)* | 0.82 (0.72-0.92)* |
|  | Etanercept | 0.97 (0.8-1.18) | 0.99 (0.98-1)* | 1.2 (1-1.43)* | 0.97 (0.96-0.98)* | 1.35 (0.98-1.82) | 1 (1-1.01) | 1.2 (0.99-1.46) | 1.85 (1.49-2.31)* | 0.87 (0.72-1.05) | 0.99 (0.78-1.28) | 1 (1-1.01) | 1.02 (0.9-1.15) |
|  | Golimumab | 1.08 (0.81-1.48) | 0.99 (0.98-1) | 1.01 (0.77-1.34) | 1 (0.99-1.01) | 0.88 (0.48-1.49) | 1 (0.99-1) | 0.75 (0.57-0.99)* | 2.46 (1.72-3.63)* | 1.11 (0.84-1.47) | 0.97 (0.68-1.45) | 0.99 (0.99-1) | 1.01 (0.83-1.23) |
|  | Rituximab | 0.83 (0.67-1.04) | 1.01 (1-1.02) | 0.66 (0.54-0.82)* | 1.01 (1-1.02) | 1.21 (0.81-1.73) | 0.99 (0.99-1)* | 1.53 (1.21-1.97)* | 0.84 (0.68-1.05) | 1.31 (1.06-1.62)* | 1.66 (1.19-2.4)* | 1 (0.99-1) | 1.54 (1.34-1.76)* |
|  | Tocilizumab | 0.85 (0.64-1.13) | 0.99 (0.98-1) | 1.27 (0.98-1.67) | 0.98 (0.97-0.99)* | 2.45 (1.66-3.49)* | 1 (0.99-1) | 1.99 (1.44-2.8)* | 1.91 (1.38-2.71)* | 1.49 (1.14-1.94)* | 3.11 (1.82-5.89)* | 0.98 (0.97-0.98)* | 1.78 (1.49-2.11)* |
| Opioid analgesic | Oxycodone | 0.88 (0.77-1.01) | 1.01 (1-1.01)* | 0.82 (0.72-0.93)* | 1.01 (1-1.01)* | 1.13 (0.89-1.42) | 1 (0.99-1)* | 1.21 (1.06-1.39)* | 0.7 (0.62-0.8)* | 1.6 (1.41-1.82)* | 4.21 (3.15-5.8)* | 0.98 (0.97-0.98)* | 2.43 (2.24-2.63)* |
|  | Tramadol | 0.81 (0.68-0.96)* | 1 (1-1.01) | 0.9 (0.77-1.07) | 1.01 (1-1.01) | 1.29 (0.95-1.7) | 0.99 (0.99-1)* | 1.73 (1.43-2.11)* | 0.72 (0.61-0.86)* | 1.32 (1.11-1.55)* | 7 (4.35-12.25)* | 0.98 (0.98-0.98)* | 2.06 (1.85-2.29)* |
| Non-opioid analgesic | Celecoxib | 1.09 (0.94-1.28) | 1 (0.99-1) | 1.2 (1.05-1.39)* | 0.98 (0.98-0.99)* | 1.18 (0.91-1.51) | 1 (1-1.01)* | 1.21 (1.04-1.41)* | 1.08 (0.93-1.25) | 1.19 (1.03-1.37)* | 1.78 (1.42-2.28)* | 1 (0.99-1)* | 1.07 (0.97-1.18) |
|  | Indometacin | 1.11 (0.79-1.59) | 1 (0.99-1.02) | 0.94 (0.69-1.28) | 1.02 (1-1.03)* | 1.07 (0.56-1.84) | 1 (0.99-1) | 0.81 (0.59-1.11) | 0.68 (0.49-0.93)* | 1.55 (1.13-2.11)* | 2.22 (1.28-4.22)* | 1 (0.99-1.01) | 1.3 (1.05-1.6)* |
|  | Meloxicam | 0.97 (0.84-1.11) | 1 (0.99-1) | 0.85 (0.75-0.97)* | 0.99 (0.98-1)* | 1.1 (0.85-1.39) | 1 (1-1) | 1.43 (1.23-1.65)* | 1 (0.87-1.14) | 1.34 (1.18-1.53)* | 1.54 (1.25-1.91)* | 0.99 (0.99-1)* | 1.08 (0.99-1.18) |
| Glucocorticoid | Prednisolone/Prednisone | 1.01 (0.92-1.11) | 1 (1-1) | 0.96 (0.88-1.04) | 1 (0.99-1) | 1.33 (1.14-1.55)* | 1 (1-1)* | 1.26 (1.14-1.38)* | 0.88 (0.8-0.96)* | 1.21 (1.11-1.32)* | 1.97 (1.7-2.29)* | 0.99 (0.99-0.99)* | 1.4 (1.32-1.48)* |
| ^#^Married vs not married (control); ^†^Age (years, continuous); ^‡^Tertiary qualified vs no tertiary qualification (control); ^§^Disease duration (years, continuous); ^**^Current smoker vs non-smoker (control); ^##^IRSAD SEIFA 2011 SA1 percentile (continuous); ^††^Female vs male sex (control); ^‡‡^Questionnaire completed online vs on paper (control); ^§§^Current depression or anxiety (EQ-5D-3L level 2 or 3) vs none (EQ-5D-3L level 1; control); ^^^Current Pain (EQ-5D-3L level 2 or 3) vs none (EQ-5D-3L level 1; control); ^+^Self-rated health (EQ-5D-3L VAS Health State Scale [0, Worst imaginable health state - 100, Best imaginable health state]; continuous); ^^^^HAQ Disability Index score (0, mild disability - 3, very severe disability; continuous; | | | | | | | | | | | | | |
| *p value <0.05 | | | | | | | | | | | | | |
